# Occurrence data on beetles (Coleoptera) collected in Dutch coastal dunes between 1953 and 1960

**DOI:** 10.3897/BDJ.10.e90103

**Published:** 2022-10-27

**Authors:** G.M. Gerrits, L. Hemerik

**Affiliations:** 1 Netherlands Institute of Ecology (NIOO-KNAW), Department of Terrestrial Ecology, Wageningen, Netherlands Netherlands Institute of Ecology (NIOO-KNAW), Department of Terrestrial Ecology Wageningen Netherlands; 2 Wageningen University and Research, Biometris Mathematical and Statistical Methods Group, Wageningen, Netherlands Wageningen University and Research, Biometris Mathematical and Statistical Methods Group Wageningen Netherlands

**Keywords:** ground-dwelling beetles, historical field data, pitfalls, Meijendel, GBIF, carabids, Meijendel Research Project

## Abstract

**Background:**

Historical field data in ecology are exceedingly rare and, therefore, their preservation and publication is of high importance, especially as these data can function as a point of reference for present day biodiversity research. Therefore, we digitised a 65-year-old dataset on ground-dwelling beetles caught with pitfall traps in the coastal dune area "Meijendel", situated in the western part of the Netherlands.

**New information:**

The data presented in this paper has never been published in a systematic way before and has had a long journey from moment of capture to the current digitisation. From 1953 through to 1960, 100 pitfalls were active and catches were collected once a week. A total of 36,400 samples were aggregated with approximately 90,000 occurrences recorded. All captures were identified up to species level and counted and sex determined where possible. The database has been registered in the Global Biodiversity Information Facility (GBIF) and can be found under: https://www.gbif.org/dataset/9d02b439-aa5c-4c22-b1d9-d27fbde9e3ee.

## Introduction

Historical field data, collected using standardised protocols, are rare in ecology. Therefore, conserving and publishing such data by means of digitisation are of high importance (Tingley and Beissinger 2009; Heberling et al. 2021), even more so because some of these datasets are documented only on paper or have been stored in professional archives and are not necessarily publicly available. Moreover, with biodiversity increasingly under pressure due to anthropogenic influences (Hallmann et al. 2017, IPBES, Intergovernmental Science-Policy Platform on Biodiversity and Ecosystem Services 2019), historical datasets are potentially of much value as they can serve as past baseline for present-day biodiversity research. Therefore, when the opportunity presented itself, we decided to manually digitise a historical dataset and its associated metadata, that was collected in the coastal dune area of "Meijendel" in the Netherlands in the period 1953-1960, in order to make it available for future study.

Around 200 archival folders with specially printed paper sheets containing the data have survived several decades of departmental shuffles and relocations and finally ended up in the attic of one of the original researchers, who then passed them on to us. The species folders contain written records of all beetle species (Coleoptera) that were caught using pitfall traps from March 1953 through to March 1960 in disparate habitats of the dune ecosystem. The folders will be transferred to Naturalis Biodiversity Centre in Leiden, The Netherlands, where already a part of the specimens, upon which this dataset is based, are stored. The use of pitfalls in this study was one of the first examples of this now much used technique and was partly inspired on beetles found trapped in manholes dug by soldiers in the war (de Bruyn, personal communication). A total of 100 of these "catch cans" (vangblikken in Dutch) were specially made from metal sheets and were dug in flush with the soil surface. The dataset has only partly been analysed before (den Boer 1956, den Boer 1958, Bouman and van Hinsberg 1991a, Bouman and van Hinsberg 1991b). Year totals from this dataset were available and have been used for ecological classifications of ground beetles by Turin (Turin et al. 1991).

The pitfalls were installed as part of a large-scale study to assess the effect of water infiltration and extraction for human consumption on the fauna and flora of the area, which itself was part of a larger project that was set up before the Second World War as one of the first standardised ecological research studies designed to collect as much long-term ecological data from one specific area as possible (Schierbeek 1923, Bakker 1974). This early example of the then burgeoning science of ecology, was set up in order to find answers to basic ecological questions regarding community composition and species' interactions with their environment (Schierbeek 1923, den Boer 1956).

The Meijendel dune area, where the pitfall study was installed, is a highly heterogeneous, species-rich landscape with alternating wet dune valleys, wind-swept dune tops above desert-like barren south-facing slopes and moist, shaded north-facing slopes. The landscape forms a finely grained mosaic-like structure with sharply defined gradients. Habitats consist of open, dry, moss- and lichen-dominated vegetations, alternated by dune grasslands with *Calamagrostisepigejos*, Festuca spp. and *Carexarenaria* and several kinds of woods, groves and shrub-dominated vegetations (Boerboom 1960, Schaminee 1995).

## Project description

### Title

Occurrence data on beetles (Coleoptera) collected in Dutch coastal dunes between 1953 and 1960

### Study area description

**Figure 1.**
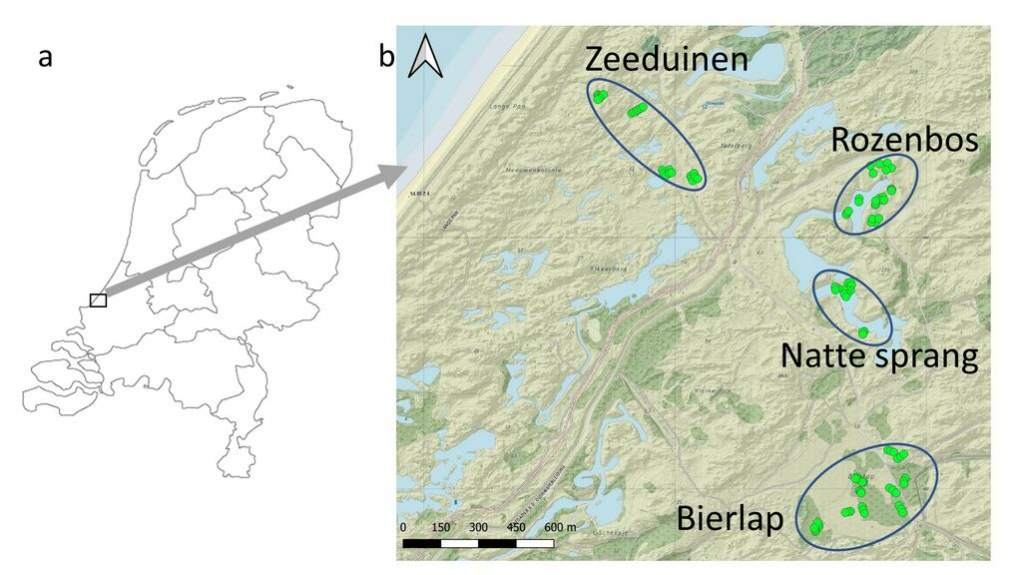
(a) Geographic location of the study area Meijendel in the Netherlands, (b) Location of the 100 pitfalls (green dots) in the Meijendel area situated within four sub-areas: "Zeeduinen" containing pitfalls 1-24; "Bierlap" with pitfalls 25-60; "Natte sprang" with pitfalls 61-75 and "Rozenbos" with pitfalls 76-100. Note that up to 1955, all of the water extraction lakes (blue areas) were absent. The lakes in the Rozenbos and Natte sprang areas were created only after the pitfall research programme was ended in 1960. For a more detailed map, see end of Suppl. material 2.

The study area "Meijendel" is a 20 km^2^ coastal dune complex running 3 kilometres inland and 7 kilometres along the coastline and is lodged between the city of The Hague in the south, the North Sea in the west, the city of Wassenaar in the northeast and adjacent dunes in the north. The water company Dunea Duin & Water manages Meijendel as a water catchment area. It is also a nature reserve enjoying protection as part of the European Natura2000 network. The area is made up of three geomorphological zones running parallel to the coastline; (1) the fore-dune complex with *Ammophilaarenaria* dominated young sand dunes, (2) a zone dominated by parabolic dunes and (3) old dune valleys with a 19^th^ century agricultural history (see Fig. 1). Pitfalls were placed in zone 2 and 3 in different vegetation types.

### Funding

This project was made possible with grants from:

1. NLBIF Netherlands Biodiversity Information Facility (grant number nlbif2020.005)
2. Drinking water company Dunea Duin & Water.

## Sampling methods

### Study extent

The geographic extent of the digitised dataset (52.14018N to 52.1557N; 4.34517E to 4.36339E) corresponds with a 2 km^2^ area in the the north-western part of the Meijendel area.

### Sampling description

Pitfalls were placed in groups of three in four subareas, namely Zeeduinen, Rozenbos, Natte sprang and Bierlap (see Suppl. material 2 for pictures of pitfalls and descriptions of sub-areas), stretching along a gradient from the fore-dune complex 400 metres from the sea to shrub and poplar-dominated old dune valleys 2,250 metres inland (see Fig. 1 and Table 1). A total of 100 pitfalls were placed in different vegetation types; (1) bare sand with low vegetation of lichens and/or mosses, (2) grassy plains with *Ammophila*, *Corynephorus*, *Festuca* and/or *Calamagrostis* dominated vegetations, (3) low shrubs such as *Hippophaerhamnoides*, *Ligustrum* or *Salixrepens* and (4) woodland consisting of stands of *Alnus*, *Betula*, *Populus* and/or *Quercus*. A total of 33 groups were placed with three pitfalls per group (Pitfall 100 was placed near a group of three others), within the four sub-areas. Sampling sites in the four sub-areas were chosen in similar vegetation types at different distances from the sea, for instance, bare sand or woody vegetations.

Sampling took place using square metal (galvanised iron) containers measuring 24 cm wide, 24 cm long and 27 cm deep. Pitfalls were dug into the ground, their top flush with the soil surface. A small hole, covered with mesh provided drainage of rain water. After the first year of sampling, protective covers were placed over the pitfalls to avoid rain, sand and debris building up inside the traps. These were made from opaque metal sheets placed a few centimetres above the trap using pins in the sand. No killing preservatives were used.

Captures were collected several times a week and aggregated into 7-day interval samples, with some exceptions where the pitfalls were aggregated either after six or eight days; for details, see the data as published on GBIF. All animals caught were identified up to species level and counted. For a sub-selection of species, sex was established. Specimens were stored in glass tubes filled with ethanol (70%). In total, 36,400 samples (100 pitfalls, 52 sampling events per year and 7 years) were collected and recorded.

Species were identified and recorded by staff from the Department of Animal Ecology of Leiden University, as well as from the Natural History Museum in Leiden, assisted by students and a number of voluntary amateur entomologists (Bouman and van Hinsberg 1991a, Bouman and van Hinsberg 1991b). Amongst them, J.T. Wiebes, W.C. Boelens, K. Bakker, A. Spoek-Haanappel, Th. van Egmond, R.E. Kooi and P.J. den Boer are mentioned by name in the metadata.

### Quality control

Taxonomic nomenclature was updated following the GBIF backbone as well as Nederlands Soortenregister (see Suppl. material 1). These are both dynamic, so this was the current state at the time of checking (spring 2022). Name lists were checked by experts from Naturalis Biodiversity Center and EIS-Kenniscentrum insecten. It should be noted, however, that species have not been re-identified using modern identification keys.

Geographic coordinates were available in the metadata as "Amersfoort coordinates". Although the metadata do not specifically mention how these coordinates were established, we presume that the water company and Leiden University had the necessary skills to measure them with high accuracy. Coordinates were plotted using GIS and transformed into decimal coordinates.

### Step description

Data were manually recorded from species' paper files into excel files. Excel files were set up with pitfalls in columns and three rows per sample date: male, female or unknown. After transferring data from a species, we checked entries in the excel files using row and column totals available in the paper files. Differences were then traced to their individual cells and adjusted when necessary. After finishing all files, we looked over all files once more thereby checking row and column totals. After digitisation, R scripts were built to transfer CSV data files into GBIF-compatible format (R scripts are available upon request). Finally, the data were published on GBIF by Hemerik and Creuwels (2022) at https://www.gbif.org/dataset/9d02b439-aa5c-4c22-b1d9-d27fbde9e3ee .

## Geographic coverage

### Description

Pitfalls were placed inside an area stretching 1,000 metres from west to east and 1,500 metres from north to south.

### Coordinates

52.14018N and 52.1557N Latitude; 4.34517E and 4.36339E Longitude.

## Taxonomic coverage

### Description

Taxonomic coverage of the dataset consists of all captures belonging to the order *Coleoptera*. In total, 267 species were recorded belonging to 110 genera from 18 families. See Table 1 for a list of families and genera caught. See also the table in Suppl. material 1 for a list of all species caught.

As with all historic data, nomenclaturial developments have given rise to alterations in species names. After comparing the historical Dutch Coleoptera catalogue (Brakman 1966) with the most recent edition (Vorst 2010), the following species must be considered.

- *Cantharisobscura* can refer to both *C.obscura* L. 1758 and *Cantharisparadoxa* Hicker, 1960

- *Amaraaulica* might refer to both *A.aulica* (Panzer, 1796) and *Amaragebleri* Dejean, 1831

- *Calathusmollisc*an mean *C.mollis* (Marsham, 1802) and *Calathuscinctus* Motschulsky, 1850

- *Pterostichusnigrita* can refer to both *P.nigrita* (Paykull, 1790) and *Pterostichusrhaeticus* Heer, 1837

- *Phyllobiuspyri* might refer to both *P.pyri* (L., 1758) and *Phyllobiusvespertinus* (F., 1793)

- *Conosomatestaceus* can mean *Sepedophilustestaceus* (F, 1793) and *Sepedophilusmarshami* (Stephens, 1832)

- *Cryptobiumfractocorne* can refer to both *Ochthephilumfracticorne* (Paykull, 1800) and *Ochthephilumcollare* (Reitter, 1884)

- *Tachyporuschrysomelinus* might refer to both *Tachyporuschrysomelinus* (L., 1758) and *Tachyporusdispar* (Paykull, 1789)

In addition, the following historic identifications might be wrongly interpreted and should be considered for adjustment in case these data are used:

- *Tychiusflavicollis* Stephens, 1831 is now considered a synonym of *Tychiusjunceus* (Reich, 1797), but this name was long misused for the species currently known as *Tychiussquamulatus* Gyllenhal, 1835. The latter is hence the correct name for *Tychiusflavicollis*.

- *Heterothopsniger* is now considered a junior synonym of *Heterothopspraevius* Erichson, 1839 (and is treated as such on GBIF).

- *Ilyobatesnigricollis* might, as well, refer to *Ilyobatesbennetti* Donisthorpe, 1914 for which the name *Ilyobatesnigricollis* was long misused.

- *Oxypodalividipennis* should refer to *Oxypodaacuminata* (Stephens, 1832), not *Nehemitropialividipennis* (Mannerheim, 1830). Mannerheim’s name *O.lividipennis* has long been misapplied to *Oxypodaacuminata*.

- The entries *Sciodrepaumbrina* and *Dreposciaumbrina* probably refer to the same specimen, but we cannot be sure.

Moreover, some identifications are interpreted as species not known to occur in the Netherlands: *Dreposciaumbrina* (Erichson, 1837), *Stenussylvester* Erichson, 1839 and *Quedionuchusplagiatus* (Mannerheim, 1843)

### Taxa included

**Table:** 

Rank	Scientific Name	
species	Coleoptera	
kingdom	Animalia	Animals
subkingdom	Bilateria	
phylum	Arthropoda	Arthropods
subphylum	Hexapoda	
class	Insecta	Insects
order	Coleoptera	Beetles
family	Apionidae	
family	Brachyceridae	
family	Byrrhidae	
family	Cantharidae	
family	Carabidae	
family	Chrysomelidae	
family	Curculionidae	
family	Dryophtoridae	
family	Elateridae	
family	Geotrupidae	
family	Histeridae	
family	Leiodidae	
family	Melolonthidae	
family	Rutelidae	
family	Silphidae	
family	Staphylinidae	
family	Tenebrionidae	
family	Zopheridae	
species	*Ceratapioncarduorum*	
species	*Melanapionminimum*	
species	*Holotrichapionononis*	
species	*Ceratapiononopordi*	
species	*Oxystomapomonae*	
species	*Apionrubens*	
species	*Apionrubiginosum*	
species	Notaris acridulus	
species	*Morychusaeneus*	
species	*Cantharisobscura*	
species	*Acupalpusmeridianus*	
species	*Agonummarginatum*	
species	*Agonummuelleri*	
species	*Amaraaenea*	
species	*Amaraapricaria*	
species	*Amaraaulica*	
species	*Amarabifrons*	
species	*Amarabrunnea*	
species	*Amaracommunis*	
species	*Amaraconvexior*	
species	*Amaracurta*	
species	*Amaraeurynota*	
species	*Amarafamelica*	
species	*Amarafamiliaris*	
species	*Amaralucida*	
species	*Amaralunicollis*	
species	*Amaraovata*	
species	*Amaraspreta*	
species	*Badisterbullatus*	
species	*Badisterlacertosus*	
species	*Bembidionassimile*	
species	*Bembidionguttula*	
species	*Bembidionlampros*	
species	*Bembidionobtusum*	
species	*Bradycelluscaucasicus*	
species	*Bradycellusharpalinus*	
species	*Broscuscephalotes*	
species	*Calathusambiguus*	
species	*Calathuserratus*	
species	*Calathusfuscipes*	
species	*Calathusmelanocephalus*	
species	*Calathusmollis*	
species	*Cicindelahybrida*	
species	*Demetriasmonostigma*	
species	*Dicheirotrichusplacidus*	
species	*Dromiusangustus*	
species	*Paradromiuslinearis*	
species	*Philorhizusmelanocephalus*	
species	*Dromiusquadrimaculatus*	
species	*Calodromiusspilotus*	
species	*Dyschiriustharacicus*	
species	*Elaphrusriparius*	
species	*Harpalusanxius*	
species	*Harpalusmelancholicus*	
species	*Harpalusrufipes*	
species	*Harpalusserripes*	
species	*Harpalusservus*	
species	*Harpalussmaragdinus*	
species	*Harpalustardus*	
species	*Harpaluspumilus*	
subspecies	*Harpalusxanthopuswinkleri*	
species	*Leistusferrugineus*	
species	*Leistusrufomarginatus*	
species	*Masoreuswetterhallii*	
species	*Syntomusfoveatus*	
species	*Syntomustruncatellus*	
species	*Nebriabrevicollis*	
species	*Notiophilusaquaticus*	
species	*Notiophilusbiguttatus*	
species	*Notiophilusgerminyi*	
species	*Notiophiluspalustris*	
species	*Notiophilusrufipes*	
species	*Notiophilussubstriatus*	
species	*Ophonuscordatus*	
species	*Ophonusrufibarbis*	
species	*Ophonusrupicola*	
species	*Panagaeusbipustulatus*	
species	*Agonumsexpunctatum*	
species	*Agonumviduum*	
species	*Pterostichusdiligens*	
species	*Pterostichusmelanarius*	
species	*Pterostichusminor*	
species	*Pterostichusniger*	
species	*Pterostichusnigrita*	
species	*Pterostichusoblongopunctatus*	
species	*Pterostichusstrenuus*	
species	*Poecilusversicolor*	
species	*Synuchusvivalis*	
species	*Trechusquadristriatus*	
species	*Galerucatanaceti*	
species	*Sermylassahalensis*	
species	*Anthonomusrubi*	
species	*Mogulonescrucifer*	
species	*Ceutorhynchushirtulus*	
species	*Nedyusquadrimaculatus*	
species	*Cleonispigra*	
species	*Cossonuslinearis*	
species	*Dorytomusdejeani*	
species	*Dorytomushirtipennis*	
species	*Dorytomuslongimanus*	
species	*Dorytomustortrix*	
species	*Dorytomusictor*	
species	*Rhinusacollina*	
species	*Rhinusalinariae*	
species	*Brachyperadauci*	
species	*Hyperanigrirostris*	
species	*Hyperaplantaginis*	
species	*Hyperapostica*	
species	*Limobiusborealis*	
species	*Limobiusmixtus*	
species	*Orchestesfagi*	
species	*Orthochaetessetiger*	
species	*Otiorhynchusovatus*	
species	*Philopedonplagiatum*	
species	*Phyllobiusargentatus*	
species	*Phyllobiuspyri*	
species	*Polydrususcervinus*	
species	*Charagmusgriseus*	
species	*Strophosomamelanogrammum*	
species	*Strophosomacapitatum*	
species	*Tychiusflavicollis*	
species	*Tychiusquinquepunctatus*	
species	*Sitophilusgranarius*	
species	*Ectinusaterrimus*	
species	*Agrioteslineatus*	
species	*Agriotesobscurus*	
species	*Agrypnusmurinus*	
species	*Cardiophorusasellus*	
species	*Dalopiusmarginatus*	
species	*Cidnopusaeruginosus*	
species	*Melanotuspunctolineatus*	
species	*Melanotusvillosus*	
species	*Prosternontessellatum*	
species	*Selatosomusaeneus*	
species	*Trypocoprisvernalis*	
species	*Saprinusaeneus*	
species	*Saprinusimmundus*	
species	*Saprinussemistriatus*	
species	*Catopschrysomeloides*	
species	*Catopscoracinus*	
species	*Catopsmorio*	
species	*Catopsnigricans*	
species	*Catopstristis*	
species	*Cholevajeanneli*	
species	*Cholevaoblonga*	
species	*Cholevapaskoviensis*	
species	*Dreposciaumbrina*	
species	*Sciodrepoidesfumatus*	
species	*Sciodrepoideswatsoni*	
species	*Polyphyllafullo*	
species	*Sericabrunnea*	
species	*Phylloperthahorticola*	
species	*Nicrophorushumator*	
species	*Nicrophorusinvestigator*	
species	*Nicrophorusvespilloides*	
species	*Thanatophilusrugosus*	
species	*Oiceoptomathoracicum*	
species	*Acidotacruentata*	
species	*Aleocharacurtula*	
species	*Aleochararuficornis*	
species	*Aleocharasparsa*	
species	*Amischaanalis*	
species	*Drusillacanaliculata*	
species	*Dinaraeaaequata*	
species	*Dinaraeaangustula*	
species	*Acrotonaaterrima*	
species	*Athetacrassicornis*	
species	*Athetaeuryptera*	
species	*Mocytafungi*	
species	*Athetagagatina*	
species	*Athetaharwoodi*	
species	*Lioglutamicroptera*	
species	*Mocytaorphana*	
species	*Athetasodalis*	
species	*Blediuspygmeus*	
species	*Lordithonthoracicus*	
species	*Bolitobiuscastaneus*	
species	*Sepedophilusimmaculatus*	
species	*Sepedophiluspedicularius*	
species	*Sepedophilustestaceus*	
species	*Creophilusmaxillosus*	
species	*Ochthephilumfracticorne*	
species	*Falagriomathoracica*	
species	*Gyrohypnusangustatus*	
species	*Gyrohypnusatratus*	
species	*Gyrohypnuspunctulatus*	
species	*Heterothopsdissimilis*	
species	*Heterothopsniger*	
species	*Heterothopsquadripunctulus*	
species	*Ilyobatesnigricollis*	
species	*Anthobiumatrocephalum*	
species	*Anthobiumunicolor*	
species	*Lathrobiumfulvipenne*	
species	*Lathrobiumgeminum*	
species	*Lobrathiummultipunctum*	
species	*Suniusmelanocephalus*	
species	*Mycetoporusbaudueri*	
species	*Mycetoporuslepidus*	
species	*Mycetoporusclavicornis*	
species	*Mycetoporusforticornis*	
species	*Mycetoporuspunctus*	
species	*Ischnosomasplendidum*	
species	*Zyrascollaris*	
species	*Zyrasfunestus*	
species	*Zyraslaticollis*	
species	*Zyraslimbatus*	
species	*Pellalugens*	
species	*Ocaleabadia*	
species	*Ocypusaeneocephalus*	
species	*Tasgiusater*	
species	*Ocypusbrunnipes*	
species	*Tasgiusmorcitans*	
species	*Ocypuspicipennis*	
species	*Omaliumcaesum*	
species	*Omaliumitalicum*	
species	*Omaliumrivulare*	
species	*Othiussubuliformis*	
species	*Othiuspunctulatus*	
species	*Ousipaliacaesula*	
species	*Oxypodabrachyptera*	
species	*Oxypodaexoleta*	
species	*Oxypodainduta*	
species	*Nehemitropialividipennis*	
species	*Oxypodaopaca*	
species	*Oxypodaprocerula*	
species	*Oxypodaspectabilis*	
species	*Oxypodatogata*	
species	*Oxypodavittata*	
species	*Oxyteluslaqueatus*	
species	*Anotylusrugosus*	
species	*Paederidusruficollis*	
species	*Metopsiaclypeata*	
species	*Quediuspersimilis*	
species	*Quediusboops*	
species	*Quediuscurtipennis*	
species	*Quediusfuliginosus*	
species	*Quediuslateralis*	
species	*Quediuslongicornis*	
species	*Quediusmolochinus*	
species	*Quediusnigrocaeruleus*	
species	*Quediusnitipennis*	
species	*Quediuspicipes*	
species	*Quedionuchusplagiatus*	
species	*Quediussemiaeneus*	
species	*Quediussemiobscurus*	
species	*Geostibacircellaris*	
species	*Stenusclavicornis*	
species	*Stenusgeniculatus*	
species	*Stenusimpressus*	
species	*Stenussylvester*	
species	*Rugilusrufipes*	
species	*Tachinuscorticinus*	
species	*Tachinusmarginellus*	
species	*Tachyporusatriceps*	
species	*Tachyporuschrysomelinus*	
species	*Tachyporushypnorum*	
species	*Tachyporuspusillus*	
species	*Tachyporusscitulus*	
species	*Tachyporustersus*	
species	*Xantholinuslaevigatus*	
species	*Xantholinuslinearis*	
species	*Xantholinuslongiventris*	
species	*Xantholinuselegans*	
species	*Xantholinustricolor*	
species	*Crypticusquisquilius*	
species	Isomira murina	
species	*Melanimontibialis*	
species	*Cylindrinotuspallidus*	
species	*Phylan gibbus*	
species	*Opatrumsabulosum*	
species	*Orthocerusclavicornis*	

## Temporal coverage

### Notes

The pitfalls collected specimens from 1 March 1953 through to 16 March 1960. Pitfalls 61 – 100 were placed on 1 March 1953. Pitfalls 1 – 60 on the 4^th^ of March. The 30 pitfalls with numbers > 100 (see below) were placed on 9 April 1959.

## Usage licence

### Usage licence

Creative Commons Public Domain Waiver (CC-Zero)

## Data resources

### Data package title

Meijendel research 1953-1960

### Resource link

https://www.gbif.org/dataset/9d02b439-aa5c-4c22-b1d9-d27fbde9e3ee

### Number of data sets

1

### Data set 1.

#### Data set name

Meijendel research 1953-1960

#### Data format

csv

#### Description

The dataset contains occurrence data from 100 pitfalls that were collected weekly from March 1953 – March 1960. The dataset contains 267 beetle species together with a number of mammal species. A description of column headers used is given below.

**Data set 1.** 

Column label	Column description
basisOfRecord	state of the recorded specimen.
class	class name.
coordinateUncertaintyInMetres	The horizontal uncertainty distance (in metres) from the given decimalLatitude and decimalLongitude.
country	The name of the country in which the Location occurs.
countryCode	The standard code for the country in which the Location occurs.
decimalLatitude	The geographic latitude (in decimal degrees, using the spatial reference system given in geodeticDatum) of the geographic centre of a Location.
decimalLongitude	The geographic longitude (in decimal degrees, using the spatial reference system given in geodeticDatum) of the geographic centre of a Location.
eventDate	date of registration of event.
eventID	Unique identifier for each event per date, per pitfall.
eventRemarks	additional information on event status.
eventTime	dates in between which the event is created.
family	family name.
lifestage	lifestage at which specimen was caught.
geodeticDatum	The coordinate system and set of reference points upon which the geographic coordinates are based.
individualCount	number of recorded specimens per occurrenceID.
kingdom	kingdom name.
locality	The specific name of the place of occurrence.
locationID	unique code for each sampling location.
occurrenceID	Unique identifier for each occurrence per species, per date, per pitfall.
occurrenceRemarks	additional information on occurrence status.
occurrenceStatus	present.
order	order name.
original_identified_as	originally identified as species.
ownerInstitutionCode	the institution having custody of the object.
phylum	phylum name.
recordedBy	institution by which specimen is identified and recorded.
samplingEffort	manner in which sampling is performed.
samplingProtocol	type of sampling technique used.
sampleSizeUnit	unit in which samples are assembled.
sampleSizeValue	number of sample units (days) of the eventID.
short.scientific.name	Eightletter abbreviation of Genus and species name of original scientific name.
startDayOfYear	day of the year from start of year.
scientificName	current scientific name.
taxonRank	taxonomic level to which specimen is identified.
type	sex.
verbatimLatitude	latitude as originally recorded.
verbatimLongitude	longitude as originally recorded.
verbatimSRS	The original coordinate system and set of reference points upon which the verbatim latitude and longitude are based.

## Additional information

As with all datasets, there are a number of details, peculiarities and shortcomings in the data that should be taken into account when working with the dataset:

Pitfalls 7-18 were moved to a new location in 1955. This was because the infiltration of river water into the dunes created lakes and these pitfalls would be flooded if not moved. On 20 June 1955, pitfalls 7-12 were moved 8 metres to the north. On 19 August 1955, pitfalls 13, 14 and 15 were moved 50 metres northwest on to the newly-formed peninsula inside the newly-formed infiltration lake. Lastly, pitfalls 16, 17 and 18 were placed on the eastern side of the same peninsula on the same date.

In 1959, an additional 30 pitfalls were placed close to their respective counterparts (number of pitfall +100), presumably to check whether the old traps were still reliable. The galvanised iron pitfalls had an increasingly rough surface due to oxidation and it was suspected that certain groups of animals (for instance spiders, see Noordam 1996) were capable of climbing out of the pitfalls. These extra pitfalls have all been added to the dataset.

In 1954, pitfalls were left unchecked for a month. On dates 26 January 1954, 2 and 9 February 1954, pitfalls were, therefore, not emptied. Presumably this was because of wintery conditions.

Some species folders only have records for 1953/1954. It cannot be guaranteed that these species were caught in later years. In other words, the paper files may not have survived. Therefore, absence of recorded captures in these cases do not constitute "hard zeros". Too many species only have record sheets for the first year or two years, to be realistic. Only a few of these species have explicit remarks on data sheets stating which years no captures were present. All other species with records for the first two years only must be treated with suspicion and restraint for subsequent years.

Part of the dataset also consists of around 5,000 records of moles (*Talpaeuropea*), mice (identified up to order) and shrews (*Sorexspec*.) caught with the pitfalls. Since no killing preservatives were used, these mammals will have eaten part of the invertebrates in that pitfall. Therefore, abundance of invertebrate catches must be viewed as minimum abundance. In case small mammals were recorded for the preceding seven day period, absence in that pitfall cannot be said to be absence in the area where the pitfall is situated and, therefore, should be handled like "soft-zeros". To a lesser degree, these soft-zeros are also the case where no mammals were caught, since no preservation killing fluids were used and carnivorous species will have preyed on other species in that pitfall.

The year 1955 saw the first outbreak of Myxomatosis in rabbits in the Netherlands, which decimated their population in Meijendel soon after. Grazing pressure from rabbits in the dune areas before Myxomatosis was high. In fact, ecologists at the time considered pressure from rabbits harmful to the vegetation and ecosystem as a whole. In the years following 1955, the first clear signs of grass and shrub encroachment were recorded. This will have had a significant influence on ground-dwelling beetle populations (see de Bruyn 1997).

Meijendel in the 1950s already had a long history of exploitation as a drinking water extraction area and provided water to the rapidly growing cities of The Hague and Leiden. As a result, Meijendel was becoming increasingly dry, with ground water levels dropping several decimetres in comparison to 19^th^ century levels. Around 1900, the area contained 600 hectares of wet dune valleys. In 1955, vegetation surveys showed only 6 hectares of this species rich habitat remained (Boerboom 1960).

## Supplementary Material

12345F5D-4ED6-502A-8BB0-76E66B66A831 10.3897/BDJ.10.e90103.suppl1 Supplementary material 1 Overview of all beetle species included in the historical database and entered for publication in GBIF (Global Biodiversity Information Facility) Data type pdf file File: oo_734841.pdf https://binary.pensoft.net/file/734841 G.M. Gerrits and L. Hemerik

CA3CC6FB-7447-5609-9A69-FC29360517C3 10.3897/BDJ.10.e90103.suppl2 Supplementary material 2 Description and pictures of the location of the pitfalls
Data type pdf file Brief description Short descriptions of the direct surroundings as well as photographs of the pitfalls used in the Meijendel Research Project between 1953 and 1960 File: oo_734843.pdf https://binary.pensoft.net/file/734843 G.M. Gerrits and L. Hemerik

## Figures and Tables

**Table 1.** List of the taxonomic coverage of the species in the dataset (based on current GBIF backbone, spring 2022).

Kingdom	Phylum	Class	Order	Family	# genera	# species
Animalia	Arthropoda	Insecta	Coleoptera	Apionidae	1	7
Animalia	Arthropoda	Insecta	Coleoptera	Brachyceridae	1	1
Animalia	Arthropoda	Insecta	Coleoptera	Byrrhidae	1	1
Animalia	Arthropoda	Insecta	Coleoptera	Cantharidae	1	1
Animalia	Arthropoda	Insecta	Coleoptera	Carabidae	26	79
Animalia	Arthropoda	Insecta	Coleoptera	Chrysomelidae	2	2
Animalia	Arthropoda	Insecta	Coleoptera	Colydiidae	1	1
Animalia	Arthropoda	Insecta	Coleoptera	Curculionidae	18	31
Animalia	Arthropoda	Insecta	Coleoptera	Dryophthoridae	1	1
Animalia	Arthropoda	Insecta	Coleoptera	Elateridae	8	11
Animalia	Arthropoda	Insecta	Coleoptera	Geotrupidae	1	1
Animalia	Arthropoda	Insecta	Coleoptera	Histeridae	1	3
Animalia	Arthropoda	Insecta	Coleoptera	Leiodidae	4	11
Animalia	Arthropoda	Insecta	Coleoptera	Melolonthidae	2	2
Animalia	Arthropoda	Insecta	Coleoptera	Rutelidae	1	1
Animalia	Arthropoda	Insecta	Coleoptera	Silphidae	2	5
Animalia	Arthropoda	Insecta	Coleoptera	Staphylinidae	35	101
Animalia	Arthropoda	Insecta	Coleoptera	Tenebrionidae	6	6
Animalia	Arthropoda	Insecta	Coleoptera	Zopheridae	1	1
