# Supplementary material for: Occurrence data on beetles (Coleoptera) collected in Dutch coastal dunes between 1953 and 1960
Source: Biodivers Data J. 2022 Oct 27;10:e90103. doi: 10.3897/BDJ.10.e90103 (PMC9836538; doi:10.3897/BDJ.10.e90103)
Supplement: Supplementary material 1 — Overview of all beetle species included in the historical database and entered for publication in GBIF (Global Biodiversity Information Facility) [file bdj-10-e90103-s001.pdf]

Electronic Supplementary Material 1. Overview of all beetle species included in the historical database and entered for publication in GBIF (Global Biodiversity Information Facility).

Original name is understood to mean the name under which the researchers of the Meijendel research group grouped the beetles when they identified them. Many species do not have a Dutch name and that is why there is nothing in this column for these species. The last column shows the total number of adult beetles of that species caught in 1953-1960.

GBIF Family	Original name	GBIF name	Author GBIF	jaar	Name NL Soortenregister	total
Apionidae	*Apion carduorum*	*Ceratapion carduorum*	Kirby	1808	*Ceratapion carduorum*	3
	Apion minimum	*Melanapion minimum*	Herbst	1797	*Melanapion minimum*	1
	Apion ononis	*Holotrichapion ononis*	Kirby	1808	*Holotrichapion ononis*	10
	Apion onopordi	*Ceratapion onopordi*	Kirby	1808	*Ceratapion onopordi*	2
	Apion pomonae	*Oxystoma pomonae*	Fabricius	1798	*Oxystoma pomonae*	11
	Apion rubens	*Apion rubens*	Stephens	1839	*Apion rubens*	4
	Apion sanguineum	*Apion rubiginosum*	Grill	1893	*Apion rubiginosum*	9
Brachyceridae	*Erirrhinus acridulus*	*Notaris acridulus*	Linnaeus	1758	*Notaris acridula*	2
Byrrhidae	*Pedilophorus aeneus*	*Morychus aeneus*	Fabricius	1775	*Morychus aeneus*	882
Cantharidae	*Cantharis obscura*	*Cantharis obscura*	Linnaeus	1758	*Cantharis obscura*	13
Carabidae	*Acupalpus meridianus*	*Acupalpus meridianus*	Linnaeus	1760	*Acupalpus meridianus*	2
	Agonum marginatum	*Agonum marginatum*	Linnaeus	1758	*Agonum marginatum*	2
	Agonum muelleri	*Agonum muelleri*	Herbst	1784	*Agonum muelleri*	2
	Amara aenea	*Amara aenea*	DeGeer	1774	*Amara aenea*	106
	Amara apricaria	*Amara apricaria*	Paykull	1790	*Amara apricaria*	6
	Amara aulica	*Amara aulica*	Panzer	1796	*Amara aulica*	1
	Amara bifrons	*Amara bifrons*	Gyllenhal	1810	*Amara bifrons*	24
	Amara brunnea	*Amara brunnea*	Gyllenhal	1810	*Amara brunnea*	1
	Amara communis	*Amara communis*	Panzer	1797	*Amara communis*	3084
	Amara convexior	*Amara convexior*	Stephens	1828	*Amara convexior*	2799
	Amara curta	*Amara curta*	Dejean	1828	*Amara curta*	2538
	Amara eurynota	*Amara eurynota*	Panzer	1796	*Amara eurynota*	1
	Amara famelica	*Amara famelica*	Zimmerman	1832	*Amara famelica*	1
	Amara familiaris	*Amara familiaris*	Duftschmid	1812	*Amara familiaris*	66
	Amara lucida	*Amara lucida*	Duftschmid	1812	*Amara lucida*	194
	Amara lunicollis	*Amara lunicollis*	Schiødte	1837	*Amara lunicollis*	248
	Amara ovata	*Amara ovata*	Fabricius	1792	*Amara ovata*	2

Amara spreta	*Amara spreta*	Dejean	1831	*Amara spreta*	69
Badister bipustulatus	*Badister bullatus*	Schrank	1798	*Badister bullatus*	298
Badister lacertosus	*Badister lacertosus*	Sturm	1815	*Badister lacertosus*	198
Bembidion assimile	*Bembidion assimile*	Gyllenhal	1810	*Bembidion assimile*	2
Bembidion guttula	*Bembidion guttula*	Fabricius	1792	*Bembidion guttula*	2
Bembidion lampros	*Bembidion lampros*	Herbst	1784	*Bembidion lampros*	2
Bembidion obtusum	*Bembidion obtusum*	Audinet-Serville	1821	*Bembidion obtusum*	9
Bradycellus collaris	*Bradycellus caucasicus*	Chaudoir	1846	*Bradycellus caucasicus*	381
Bradycellus harpalinus	*Bradycellus harpalinus*	Audinet-Serville	1821	*Bradycellus harpalinus*	30
Broscus cephalotes	*Broscus cephalotes*	Linnaeus	1758	*Broscus cephalotes*	70
Calathus ambiguus	*Calathus ambiguus*	Paykull	1790	*Calathus ambiguus*	3403
Calathus erratus	*Calathus erratus*	Sahlberg	1827	*Calathus erratus*	5658
Calathus fuscipes	*Calathus fuscipes*	Goeze	1777	*Calathus fuscipes*	3659
Calathus melanocephalus	*Calathus melanocephalus*	Linneaus	1758	*Calathus melanocephalus*	2068
Calathus mollis	*Calathus mollis*	Marsham	1802	*Calathus mollis*	158
Cicindela hybrida	*Cicindela hybrida*	Linnaeus	1758	*Cicindela hybrida*	444
Demetrias monostigma	*Demetrias monostigma*	Samouelle	1819	*Demetrias monostigma*	6
Dicheirotrichus placidus	*Dicheirotrichus placidus*	Gyllenhal	1827	*Trichocellus placidus*	71
Dromius angustus	*Dromius angustus*	Brullé	1834	*Dromius angustus*	3
Dromius linearis	*Paradromius linearis*	Olivier	1795	*Paradromius linearis*	147
Dromius melanocephalus	*Philorhizus melanocephalus*	Dejean	1825	*Philorhizus melanocephalus*	50
Dromius quadrimaculatus	*Dromius quadrimaculatus*	Linneaus	1758	*Dromius quadrimaculatus*	17
Dromius quadrinotatus	*Calodromius spilotus*	Illiger	1798	*Calodromius spilotus*	10
Dyschirius thoracicus	*Dyschirius tharacicus*	Dawson	1854	*Dyschirius thoracicus*	3
Elaphrus riparius	*Elaphrus riparius*	Linnaeus	1758	*Elaphrus riparius*	2
Harpalus anxius	*Harpalus anxius*	Duftschmid	1812	*Harpalus anxius*	1
Harpalus melancholicus	*Harpalus melancholicus*	Dejean	1829	*Harpalus melancholicus*	1
Harpalus rufipes	*Harpalus rufipes*	De Geer	1774	*Harpalus rufipes*	6
Harpalus serripes	*Harpalus serripes*	Quensel	1790	*Harpalus serripes*	1
Harpalus servus	*Harpalus servus*	Duftschmid	1812	*Harpalus servus*	4279
Harpalus smaragdinus	*Harpalus smaragdinus*	Duftschmid	1812	*Harpalus smaragdinus*	8
Harpalus tardus	*Harpalus tardus*	Panzer	1796	*Harpalus tardus*	12
Harpalus vernalis	*Harpalus pumilus*	Sturm	1818	*Harpalus pumilus*	315
Harpalus winkleri	*Harpalus xanthopus winkleri*	Schauberger	1923	*Harpalus xanthopus winkleri*	662

	Leistus ferrugineus	*Leistus ferrugineus*	Linneaus	1758	*Leistus ferrugineus*	791
	Leistus rufomarginatus	*Leistus rufomarginatus*	Duftschmid	1812	*Leistus rufomarginatus*	5
	Masoreus wetterhallii	*Masoreus wetterhallii*	Gyllenhal	1813	*Masoreus wetterhallii*	555
	Metabletus foveatus	*Syntomus foveatus*	Geoffroy	1785	*Syntomus foveatus*	524
	Metabletus truncatellus	*Syntomus truncatellus*	Linnaeus	1760	*Syntomus truncatellus*	2880
	Nebria brevicollis	*Nebria brevicollis*	Fabricius	1792	*Nebria brevicollis*	29
	Notiophilus aquaticus	*Notiophilus aquaticus*	Linnaeus	1758	*Notiophilus aquaticus*	436
	Notiophilus biguttatus	*Notiophilus biguttatus*	Fabricius	1779	*Notiophilus biguttatus*	60
	Notiophilus hypocrita	*Notiophilus germinyi*	Fauvel	1863	*Notiophilus germinyi*	829
	Notiophilus palustris	*Notiophilus palustris*	Duftschmid	1812	*Notiophilus palustris*	217
	Notiophilus rufipes	*Notiophilus rufipes*	Curtis	1829	*Notiophilus rufipes*	26
	Notiophilus substriatus	*Notiophilus substriatus*	Waterhouse	1833	*Notiophilus substriatus*	8
	Ophonus cordatus	*Ophonus cordatus*	Duftschmid	1812	*Ophonus cordatus*	48
	Ophonus rufibarbis	*Ophonus rufibarbis*	Fabricius	1792	*Ophonus rufibarbis*	1
	Ophonus rupicola	*Ophonus rupicola*	Sturm	1818	*Ophonus rupicola*	1
	Panagaeus bipustulatus	*Panagaeus bipustulatus*	Fabricius	1775	*Panagaeus bipustulatus*	329
	Platynus sexpunctatum	*Agonum sexpunctatum*	Linnaeus	1758	*Agonum sexpunctatum*	1
	Platynus viduum	*Agonum viduum*	Panzer	1796	*Agonum viduum*	1
	Pterostichus diligens	*Pterostichus diligens*	Sturm	1824	*Pterostichus diligens*	1
	Pterostichus melanarius	*Pterostichus melanarius*	Illliger	1798	*Pterostichus melanarius*	7
	Pterostichus minor	*Pterostichus minor*	Gyllenhal	1827	*Pterostichus minor*	1
	Pterostichus niger	*Pterostichus niger*	Schaller	1783	*Pterostichus niger*	3
	Pterostichus nigrita	*Pterostichus nigrita*	Paykull	1790	*Pterostichus nigrita*	11
	Pterostichus oblongopunctatus	*Pterostichus oblongopunctatus*	Fabricius	1787	*Pterostichus oblongopunctatus*	1
	Pterostichus strenuus	*Pterostichus strenuus*	Panzer	1796	*Pterostichus strenuus*	327
	Pterostichus versicolor	*Poecilus versicolor*	Sturm	1824	*Poecilus versicolor*	6
	Synuchus nivalis	*Synuchus vivalis*	Illiger	1798	*Synuchus vivalis*	31
	Trechus quadristriatus	*Trechus quadristriatus*	Schrank	1781	*Trechus quadristriatus*	1787
Chrysomelidae	*Galeruca tanaceti*	*Galeruca tanaceti*	Linnaeus	1758	*Galeruca tanaceti*	328
	Sermyla halensis	*Sermylassa halensis*	Linnaeus	1767	*Sermylassa halensis*	52
Curculionidae	*Anthonomus rubi*	*Anthonomus rubi*	Bedel	1887	*Anthonomus rubi*	2
	Ceutorhynchus cruciger	*Mogulones crucifer*	Pallas	1771	*Mogulones crucifer*	4
	Ceutorhynchus hirtulus	*Ceutorhynchus hirtulus*	Germar	1824	*Ceutorhynchus hirtulus*	15
	Cidnorrhinus quadrimaculatus	*Nedyus quadrimaculatus*	Linnaeus	1758	*Nedyus quadrimaculatus*	4

	Cleonus piger	*Cleonis pigra*	Scopoli	1763	*Cleonis pigra*	1
	Cossonus linearis	*Cossonus linearis*	Hustache	1931	*Cossonus linearis*	9
	Eteophilus dejeani	*Dorytomus dejeani*	Faust	1882	*Dorytomus dejeani*	20
	Eteophilus hirtipennis	*Dorytomus hirtipennis*	Bedel	1884	*Dorytomus hirtipennis*	19
	Eteophilus longimanus	*Dorytomus longimanus*	Forster	1771	*Dorytomus longimanus*	29
	Eteophilus tortrix	*Dorytomus tortrix*	Linnaeus	1760	*Dorytomus tortrix*	5
	Eteophilus validirostris	*Dorytomus ictor*	Herbst	1795	*Dorytomus ictor*	2
	Gymnetrum collinum	*Rhinusa collina*	Gyllenhal	1813	*Rhinusa collina*	1
	Gymnetrum linariae	*Rhinusa linariae*	Panzer	1795	*Rhinusa linariae*	5
	Hypera fasciculata	*Brachypera dauci*	Olivier	1807	*Brachypera dauci*	66
	Hypera nigrirostris	*Hypera nigrirostris*	Fabricius	1775	*Hypera nigrirostris*	2
	Hypera plantaginis	*Hypera plantaginis*	DeGeer	1775	*Hypera plantaginis*	18
	Hypera variabilis	*Hypera postica*	Gyllenhal	1813	*Hypera postica*	1
	Limobius borealis	*Limobius borealis*	Paykull	1792	*Limobius borealis*	10
	Limobius mixtus	*Limobius mixtus*	Boheman	1834	*Limobius mixtus*	60
	Orchestes fagi	*Orchestes fagi*	Linneaeus	1758	*Orchestes fagi*	2
	Orthochaetes setiger	*Orthochaetes setiger*	Beck	1817	*Orthochaetes setiger*	1
	Otiorhynchus ovatus	*Otiorhynchus ovatus*	Linnaeus	1758	*Otiorhynchus ovatus*	350
	Philopedon plagiatus	*Philopedon plagiatum*	Schaller	1783	*Philopedon plagiatum*	3569
	Phyllobius argentatus	*Phyllobius argentatus*	Linnaeus	1758	*Phyllobius argentatus*	44
	Phyllobius piri	*Phyllobius pyri*	Linnaeus	1758	*Phyllobius pyri*	41
	Polydrosus cervinus	*Polydrusus cervinus*	Linnaeus	1758	*Polydrusus cervinus*	23
	Sitona griseus	*Charagmus griseus*	Fabricius	1775	*Sitona griseus*	177
	Strophosoma melanogrammus	*Strophosoma melanogrammum*	Forster	1771	*Strophosoma melanogrammum*	31
	Strophosoma rufipes	*Strophosoma capitatum*	De Geer	1775	*Strophosoma capitatum*	75
	Tychius flavicollis	*Tychius flavicollis*	Boheman	1843	*Tychius junceus*	2
	Tychius quinquepunctatus	*Tychius quinquepunctatus*	Linnaeus	1758	*Tychius quinquepunctatus*	1
Dryophtoridae	*Sitophilus granarius*	*Sitophilus granarius*	Linnaeus	1758	*Sitophilus granarius*	10
Elateridae	*Agriotes aterrimus*	*Ectinus aterrimus*	Linnaeus	1760	*Ectinus aterrimus*	118
	Agriotes lineatus	*Agriotes lineatus*	Linnaeus	1767	*Agriotes lineatus*	1
	Agriotes obscurus	*Agriotes obscurus*	Linnaeus	1758	*Agriotes obscurus*	88
	Brachylacon murinus	*Agrypnus murinus*	Linnaeus	1758	*Agrypnus murinus*	987
	Cardiophorus asellus	*Cardiophorus asellus*	Erichson	1840	*Cardiophorus asellus*	389
	Dolopius marginatus	*Dalopius marginatus*	Linnaeus	1758	*Dalopius marginatus*	47

	Limonius aeruginosus	*Cidnopus aeruginosus*	Olivier	1790	*Cidnopus aeruginosus*	140
	Melanotus punctolineatus	*Melanotus punctolineatus*	Pelerin	1829	*Melanotus punctolineatus*	44
	Melanotus rufipes	*Melanotus villosus*	Geoffroy	1785	*Melanotus villosus*	3
	Prosternon holosericeus	*Prosternon tessellatum*	Linnaeus	1758	*Prosternon tessellatum*	33
	Selatosomus aeneus	*Selatosomus aeneus*	Linnaeus	1758	*Selatosomus aeneus*	172
Geotrupidae	*Geotrupes vernalis*	*Trypocopris vernalis*	Linnaeus	1758	*Geotrupes vernalis*	11
Histeridae	*Saprinus aeneus*	*Saprinus aeneus*	Fabricius	1775	*Saprinus aeneus*	38
	Saprinus immundus	*Saprinus immundus*	Gyllenhal	1827	*Saprinus immundus*	10
	Saprinus semistriatus	*Saprinus semistriatus*	Scriba	1790	*Saprinus semistriatus*	275
Leiodidae	*Catops chrysomeloides*	*Catops chrysomeloides*	Panzer	1798	*Catops chrysomeloides*	674
	Catops coracinus	*Catops coracinus*	Kellner	1846	*Catops coracinus*	573
	Catops morio	*Catops morio*	Fabricius	1787	*Catops morio*	663
	Catops nigricans	*Catops nigricans*	Spence	1813	*Catops nigricans*	3116
	Catops tristis	*Catops tristis*	Panzer	1793	*Catops tristis*	848
	Choleva jeanneli	*Choleva jeanneli*	Britten	1922	*Choleva jeanneli*	71
	Choleva oblonga	*Choleva oblonga*	Latreille	1806	*Choleva oblonga*	102
	Choleva paskoviensis	*Choleva paskoviensis*	Reitter	1913	*Choleva paskoviensis*	39
	Dreposcia umbrina	*Dreposcia umbrina*	Erichson	1837	*NA*	901
	Sciodrepoides fumatus	*Sciodrepoides fumatus*	Spence	1813	*Sciodrepoides fumatus*	998
	Sciodrepoides watsoni	*Sciodrepoides watsoni*	Spence	1813	*Sciodrepoides watsoni*	1485
Melolonthidae	*Polyphylla fullo*	*Polyphylla fullo*	Linnaeus	1758	*Polyphylla fullo*	11
	Serica brunnea	*Serica brunnea*	Linnaeus	1758	*Serica brunna*	313
?	*Sciodrepa umbrina*					901
Rutelidae	*Phyllopertha horticola*	*Phyllopertha horticola*	Linnaeus	1758	*Phyllopertha horticola*	169
Silphidae	*Necrophorus humatus*	*Nicrophorus humator*	Gleditsch	1767	*Nicrophorus humator*	119
	Necrophorus investigator	*Nicrophorus investigator*	Zetterstedt	1824	*Nicrophorus investigator*	534
	Necrophorus vespilloides	*Nicrophorus vespilloides*	Herbst	1783	*Nicrophorus vespilloides*	187
	Thanatophilus rugosus	*Thanatophilus rugosus*	Linnaeus	1758	*Thanatophilus rugosus*	91
	Thanatophilus thoracicum	*Oiceoptoma thoracicum*	Linnaeus	1758	*Oiceoptoma thoracicum*	368
Staphylinidae	*Acidota cruentata*	*Acidota cruentata*	Mannerheim	1830	*Acidota cruentata*	99
	Aleochara curtula	*Aleochara curtula*	Goeze	1777	*Aleochara curtula*	1
	Aleochara ruficornis	*Aleochara ruficornis*	Gravenhorst	1802	*Aleochara ruficornis*	2
	Aleochara sparsa	*Aleochara sparsa*	Heer	1839	*Aleochara sparsa*	1
	Amischa analis	*Amischa analis*	Gravenhorst	1802	*Amischa analis*	1

Astilbus canaliculatus	*Drusilla canaliculata*	Fabricius	1787	*Drusilla canaliculata*	13842
Atheta aequata	*Dinaraea aequata*	Erichson	1837	*Dinaraea aequata*	6
Atheta angustula	*Dinaraea angustula*	Gyllenhal	1810	*Dinaraea angustula*	2
Atheta aterrima	*Acrotona aterrima*	Gravenhorst	1802	*Acrotona aterrima*	1
Atheta crassicornis	*Atheta crassicornis*	Fabricius	1792	*Atheta crassicornis*	6
Atheta euryptera	*Atheta euryptera*	Stephens	1832	*Atheta euryptera*	1
Atheta fungi	*Mocyta fungi*	Gravenhorst	1806	*Acrotona fungi*	3
Atheta gagatina	*Atheta gagatina*	Baudi	1848	*Atheta gagatina*	6
Atheta harwoodi	*Atheta harwoodi*	Williams	1930	*Atheta harwoodi*	4
Atheta oblongiuscula	*Liogluta microptera*	Thomson	1867	*Liogluta microptera*	3
Atheta orphana	*Mocyta orphana*	Erichson	1837	*Acrotona orphana*	1
Atheta sodalis	*Atheta sodalis*	Erichson	1837	*Atheta sodalis*	7
Bledius pusillus	*Bledius pygmeus*	Erichson	1839	*Bledius pusillus*	7
Bolitobius thoracicus	*Lordithon thoracicus*	Fabricius	1777	*Lordithon thoracicus*	3
Bryocharis analis	*Bolitobius castaneus*	Stephens	1832	*Bolitobius castaneus*	143
Conosoma immaculatus	*Sepedophilus immaculatus*	Stephens	1832	*Sepedophilus immaculatus*	8
Conosoma pedicularius	*Sepedophilus pedicularius*	Gravenhorst	1802	*Sepedophilus pedicularius*	18
Conosoma testaceus	*Sepedophilus testaceus*	Fabricius	1792	*Sepedophilus testaceus*	42
Creophilus maxillosus	*Creophilus maxillosus*	Linnaeus	1758	*Creophilus maxillosus*	1
Cryptobium fractocorne	*Ochthephilum fracticorne*	Paykull	1800	*Ochthephilum fracticorne*	5
Falagria thoracica	*Falagrioma thoracica*	Stephens	1832	*Falagrioma thoracica*	51
Gyrohypnus angustatus	*Gyrohypnus angustatus*	Stephens	1833	*Gyrohypnus angustatus*	268
Gyrohypnus atratus	*Gyrohypnus atratus*	Heer	1839	*Gyrohypnus atratus*	3
Gyrohypnus punctulatus	*Gyrohypnus punctulatus*	Paykull	1789	*Gyrohypnus punctulatus*	2
Heterothops dissimilis	*Heterothops dissimilis*	Gravenhorst	1802	*Heterothops dissimilis*	27
Heterothops niger	*Heterothops niger*	Kraatz	1868	*Heterothops niger*	1
Heterothops quadripunctulus	*Heterothops quadripunctulus*	Gravenhorst	1806	*Heterothops quadripunctulus*	1
Ilyobates nigricollis	*Ilyobates nigricollis*	Paykull	1800	*Ilyobates nigricollis*	5
Lathrimaeum atrocephalum	*Anthobium atrocephalum*	Gyllenhal	1827	*Anthobium atrocephalum*	869
Lathrimaeum unicolor	*Anthobium unicolor*	Marsham	1802	*Anthobium unicolor*	616
Lathrobium fulvipenne	*Lathrobium fulvipenne*	Gravenhorst	1806	*Lathrobium fulvipenne*	1
Lathrobium geminum	*Lathrobium geminum*	Kraatz	1857	*Lathrobium geminum*	45
Lathrobium multipunctatum	*Lobrathium multipunctum*	Gravenhorst	1802	*Lobrathium multipunctum*	1
Medon melanocephalus	*Sunius melanocephalus*	Fabricius	1792	*Sunius melanocephalus*	4

Mycetoporus baudueri	*Mycetoporus baudueri*	Mulsant & Rey	1875	*Mycetoporus baudueri*	93
Mycetoporus brunneus	*Mycetoporus lepidus*	Gravenhorst	1806	*Mycetoporus lepidus*	1
Mycetoporus clavicornis	*Mycetoporus clavicornis*	Stephens	1832	*Mycetoporus clavicornis*	7
Mycetoporus forticornis	*Mycetoporus forticornis*	Fauvel	1875	*Mycetoporus forticornis*	4
Mycetoporus punctus	*Mycetoporus punctus*	Gravenhorst	1806	*Mycetoporus punctus*	6
Mycetoporus splendidus	*Ischnosoma splendidum*	Gravenhorst	1806	*Ischnosoma splendidum*	30
Myrmedonia collaris	*Zyras collaris*	Paykull	1789	*Zyras collaris*	14
Myrmedonia funestus	*Zyras funestus*	Gravenhorst	1806	*Zyras funestus*	960
Myrmedonia laticollis	*Zyras laticollis*	Maerkel	1842	*Zyras laticollis*	109
Myrmedonia limbatus	*Zyras limbatus*	Paykull	1789	*Zyras limbatus*	1
Myrmedonia lugens	*Pella lugens*	Gravenhorst	1802	*Zyras lugens*	59
Ocalea badia	*Ocalea badia*	Erichson	1837	*Ocalea badia*	1
Ocypus aeneocephalus	*Ocypus aeneocephalus*	De Geer	1774	*Ocypus aeneocephalus*	206
Ocypus ater	*Tasgius ater*	Gravenhorst	1802	*Tasgius ater*	100
Ocypus brunnipes	*Ocypus brunnipes*	Fabricius	1781	*Ocypus brunnipes*	1066
Ocypus compressus	*Tasgius morsitans*	Rossi	1790	*Tasgius morsitans*	194
Ocypus picipennis	*Ocypus picipennis*	Fabricius	1792	*Ocypus picipennis*	1507
Omalium caesum	*Omalium caesum*	Gravenhorst	1806	*Omalium caesum*	2
Omalium italicum	*Omalium italicum*	Bernhauer	1902	*Omalium italicum*	4
Omalium rivulare	*Omalium rivulare*	Paykull	1789	*Omalium rivulare*	40
Othius myrmecophilus	*Othius subuliformis*	Stephens	1833	*Othius subuliformis*	112
Othius punctulatus	*Othius punctulatus*	Goeze	1877	*Othius punctulatus*	202
Ousipalia caesula	*Ousipalia caesula*	Erichson	1839	*Ousipalia caesula*	1
Oxypoda brachyptera	*Oxypoda brachyptera*	Stephens	1832	*Oxypoda brachyptera*	5
Oxypoda exoleta	*Oxypoda exoleta*	Erichson	1839	*Oxypoda exoleta*	1
Oxypoda induta	*Oxypoda induta*	Mulsant & Rey	1861	*Oxypoda induta*	1
Oxypoda lividipennis	*Nehemitropia lividipennis*	Mannerheim	1830	*Nehemitropia lividipennis*	202
Oxypoda opaca	*Oxypoda opaca*	Gravenhorst	1802	*Oxypoda opaca*	2
Oxypoda procerula	*Oxypoda procerula*	Mannerheim	1830	*Oxypoda procerula*	1
Oxypoda spectabilis	*Oxypoda spectabilis*	Maerkel	1844	*Oxypoda spectabilis*	1
Oxypoda togata	*Oxypoda togata*	Erichson	1837	*Oxypoda togata*	7
Oxypoda vittata	*Oxypoda vittata*	Maerkel	1842	*Oxypoda vittata*	1
Oxytelus laqueatus	*Oxytelus laqueatus*	Marsham	1802	*Oxytelus laqueatus*	1
Oxytelus rugosus	*Anotylus rugosus*	Fabricius	1775	*Anotylus rugosus*	2

Paederus ruficollis	*Paederidus ruficollis*	Fabricius	1777	*Paederidus ruficollis*	1
Phloeobium clypeatum	*Metopsia clypeata*	Mueller	1821	*Metopsia clypeata*	1
Quedius aridulus	*Quedius persimilis*	Mulsant & Rey	1876	*Quedius persimilis*	2
Quedius boops	*Quedius boops*	Gravenhorst	1802	*Quedius boops*	19
Quedius curtipennis	*Quedius curtipennis*	Bernhauer	1908	*Quedius curtipennis*	7
Quedius fuliginosus	*Quedius fuliginosus*	Gravenhorst	1802	*Quedius fuliginosus*	326
Quedius lateralis	*Quedius lateralis*	Gravenhorst	1802	*Quedius lateralis*	3
Quedius longicornis	*Quedius longicornis*	Kraatz	1857	*Quedius longicornis*	10
Quedius molochinus	*Quedius molochinus*	Gravenhorst	1806	*Quedius molochinus*	541
Quedius nigrocaeruleus	*Quedius nigrocaeruleus*	Fauvel	1876	*Quedius nigrocaeruleus*	4
Quedius nitipennis	*Quedius nitipennis*	Stephens	1833	*Quedius nitipennis*	6
Quedius picipes	*Quedius picipes*	Mannerheim	1830	*Quedius picipes*	115
Quedius plagiatus	*Quedionuchus plagiatus*	Mannerheim	1843	*NA*	1
Quedius semiaeneus	*Quedius semiaeneus*	Stephens	1833	*Quedius semiaeneus*	18
Quedius semiobscurus	*Quedius semiobscurus*	Marsham	1802	*Quedius semiobscurus*	40
Sipalia circellaris	*Geostiba circellaris*	Gravenhorst	1802	*Geostiba circellaris*	13
Stenus clavicornis	*Stenus clavicornis*	Scopoli	1763	*Stenus clavicornis*	1048
Stenus geniculatus	*Stenus geniculatus*	Gravenhorst	1806	*Stenus geniculatus*	3
Stenus impressus	*Stenus impressus*	Germar	1823	*Stenus impressus*	50
Stenus sylvester	*Stenus sylvester*	Erichson	1839	*NA*	3
Stilicus rufipes	*Rugilus rufipes*	Germar	1836	*Rugilus rufipes*	44
Tachinus corticinus	*Tachinus corticinus*	Gravenhorst	1802	*Tachinus corticinus*	72
Tachinus marginellus	*Tachinus marginellus*	Fabricius	1781	*Tachinus marginellus*	8
Tachyporus atriceps	*Tachyporus atriceps*	Stephens	1832	*Tachyporus atriceps*	28
Tachyporus chrysomelinus	*Tachyporus chrysomelinus*	Linnaeus	1758	*Tachyporus chrysomelinus*	2
Tachyporus hypnorum	*Tachyporus hypnorum*	Fabricius	1775	*Tachyporus hypnorum*	1
Tachyporus pusillus	*Tachyporus pusillus*	Gravenhorst	1806	*Tachyporus pusillus*	77
Tachyporus scitulus	*Tachyporus scitulus*	Erichson	1839	*Tachyporus scitulus*	4
Tachyporus tersus	*Tachyporus tersus*	Erichson	1839	*Tachyporus tersus*	1
Xantholinus laevigatus	*Xantholinus laevigatus*	Jacobsen	1849	*Xantholinus laevigatus*	104
Xantholinus linearis	*Xantholinus linearis*	Olivier	1795	*Xantholinus linearis*	2447
Xantholinus longiventris	*Xantholinus longiventris*	Heer	1839	*Xantholinus longiventris*	19
Xantholinus semirufus	*Xantholinus elegans*	Olivier	1795	*Xantholinus elegans*	269
Xantholinus tricolor	*Xantholinus tricolor*	Fabricius	1787	*Xantholinus tricolor*	31

Tenebrionidae	*Crypticus quisquilius*	*Crypticus quisquilius*	Linnaeus	1760	*Crypticus quisquilius*	698
	Isomira murina	*Isomira murina*	Linnaeus	1758	*Isomira murina*	90
	Microzoum tibialis	*Melanimon tibialis*	Fabricius	1781	*Melanimon tibialis*	2249
	Nalassus pallidus	*Cylindrinotus pallidus*	Curtis	1830	*Xanthomus pallidus*	15
	Olocrates gibbus	*Phylan gibbus*	Fabricius	1775	*Phylan gibbus*	3408
	Opatrum sabulosum	*Opatrum sabulosum*	Linnaeus	1761	*Opatrum sabulosum*	70
Zopheridae	*Orthocerus muticus*	*Orthocerus clavicornis*	Linnaeus	1758	*Orthocerus clavicornis*	149
