# Supplementary material for: Occurrence data on beetles (Coleoptera) collected in Dutch coastal dunes between 1953 and 1960
Source: Biodivers Data J. 2022 Oct 27;10:e90103. doi: 10.3897/BDJ.10.e90103 (PMC9836538; doi:10.3897/BDJ.10.e90103)
Supplement: Supplementary material 2 — Description and pictures of the location of the pitfalls [file bdj-10-e90103-s002.pdf]

Electronic Supplementary Material 2:

Meijendel research 1953-1960: Description and pictures of the location of the pitfalls.

Vegetation characteristics and terrain descriptions are provided below, together with photographs (scanned by Aart Noordam) of the 100 pitfalls and their surroundings. The habitats around the pitfalls were discerned into 11 categories by Bouman & van Hinsberg (1991) as shown in Table ESM2.1

Table ESM2.1 habitat descriptions

Habitat	Description of habitat
1	bare sand with little or no vegetation
2	open terrain with light beach grass (*Ammophila arenaria*) vegetation
3	low overgrowth of mosses, lichens, etc.
4	open terrain with dense vegetation of dune reed (*Calamagrostis epigejos*), etc.
5	birch forest
6	forest of aspen (*Populus tremulus*)
7	sea buckthorn (*Hippophae rhamnoides*) thicket
8	forest with a lot of common hop (*Humulus lupulus*)
9	creeping willow (*Salix repens*) bushes
10	forest of black poplar (*Populus nigra*)
11	scattered birches with mainly dune reed (*Calamagrostis epigejos*) as undergrowth

1. **Zeeduinen, pitfalls 1-24**

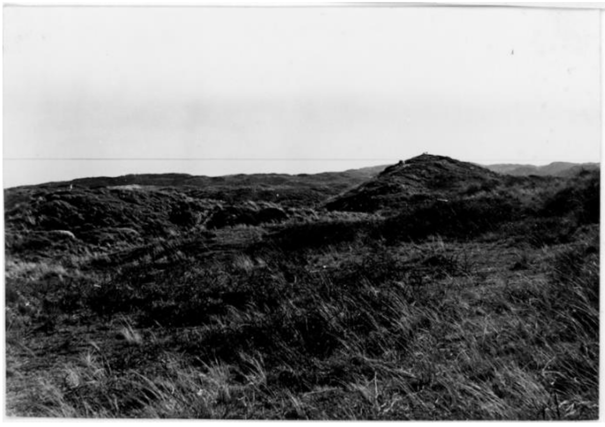

Dunes seen from pitfalls 1-6 in April 1955

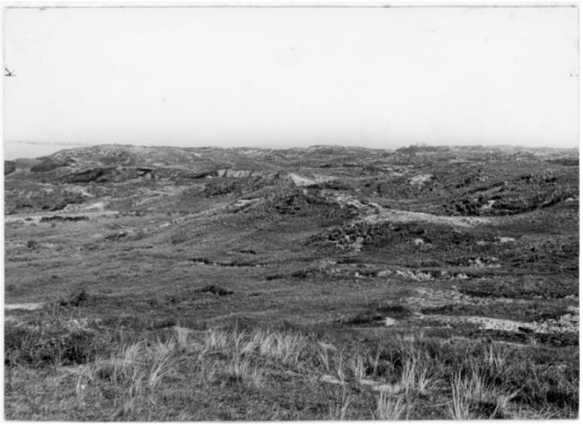

Another view from pitfalls 1-6 in April 1955

pitfalls 1-3	bare sand with little or no vegetation
pitfalls 4-6	open terrain with light beach grass (*Ammophila arenaria*) vegetation
pitfalls 7-9	low overgrowth of mosses, lichens, etc.
pitfalls 10-12	low overgrowth of mosses, lichens, etc.
pitfalls 13-15	birch forest
pitfalls 16-18	bare sand with little or no vegetation
pitfalls 19-21	open terrain with dense vegetation of dune reed (*Calamagrostis epigejos*), etc.
pitfalls 22-24	birch forest

Pitfalls 1-6 are located in the sea dunes, directly behind the Rijnland fence. The numbers 1, 2 and 4 are in open sand with very little vegetation 3, 5 and 6 are situated in parts with beach grass (*Ammophila arenaria*)

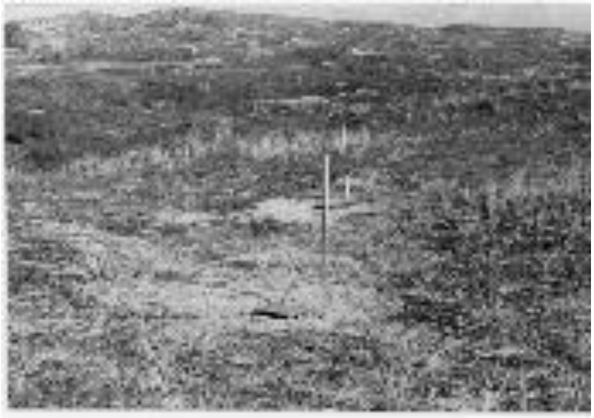

Pitfall 1 in April 1955

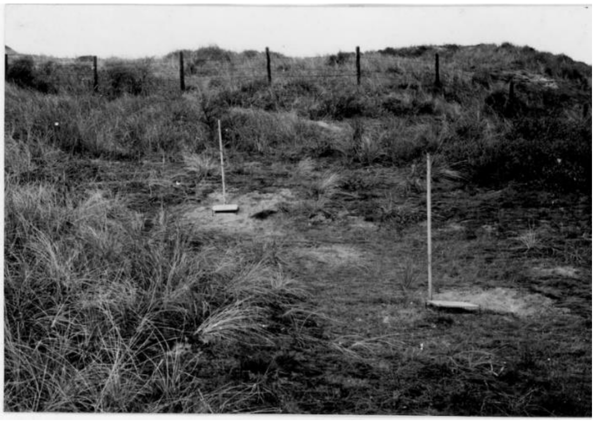

Pitfall 2 in April 1955 with in the background pitfall 1

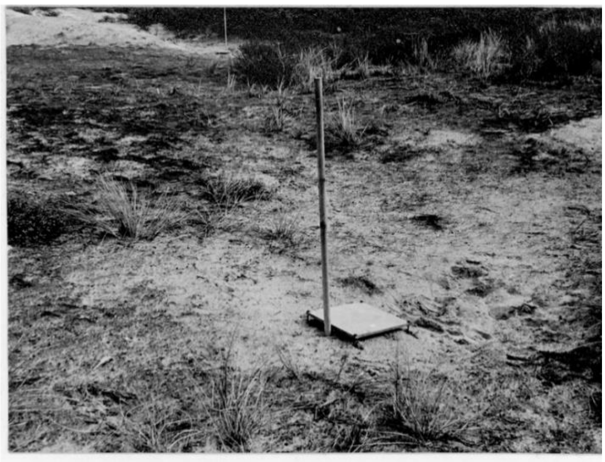

Pitfall 2 in April 1955 with in the background pitfall 4

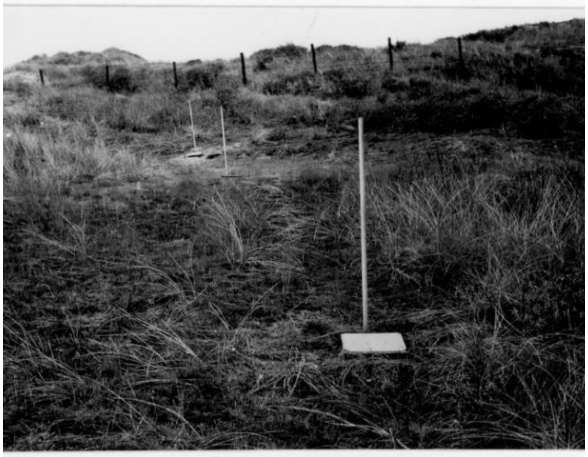

Pitfall 3 in April 1955 with in the background pitfalls 1 and 2

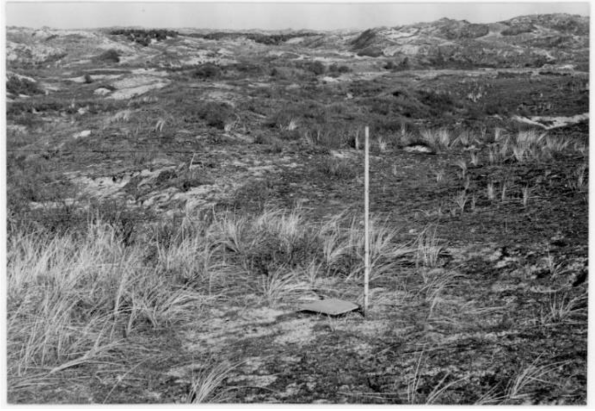

Pitfall 3 in April 1955

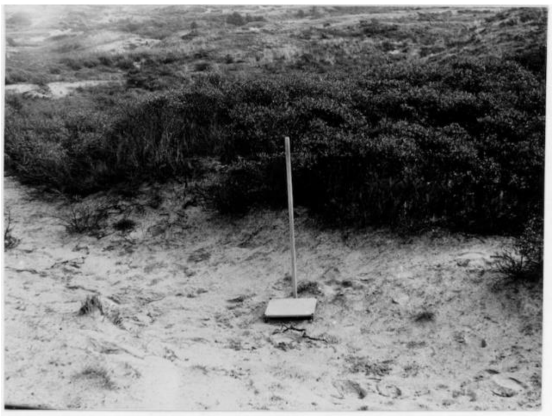

Pitfall 4 in April 1955

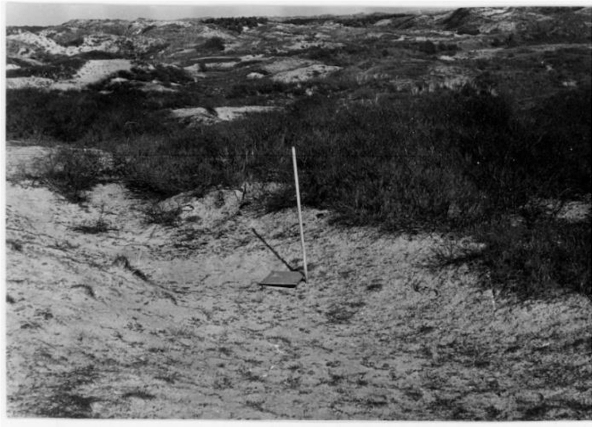

Another view on pitfall 4 in April 1955

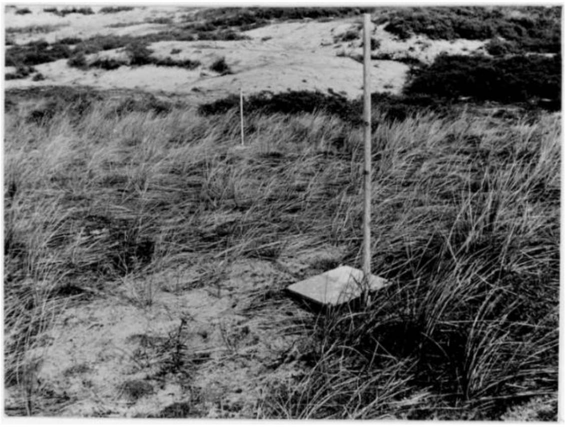

Pitfall 5 in April 1955 with in the background pitfall 3

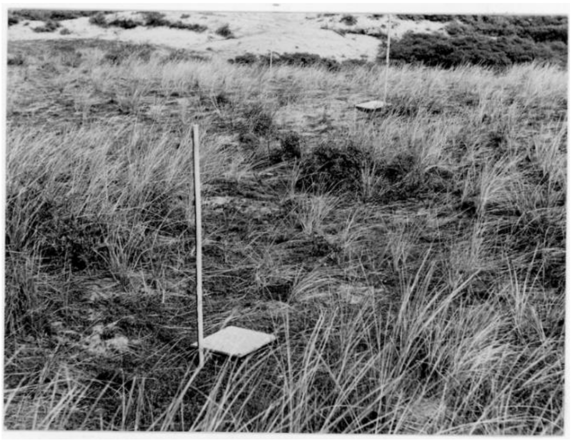

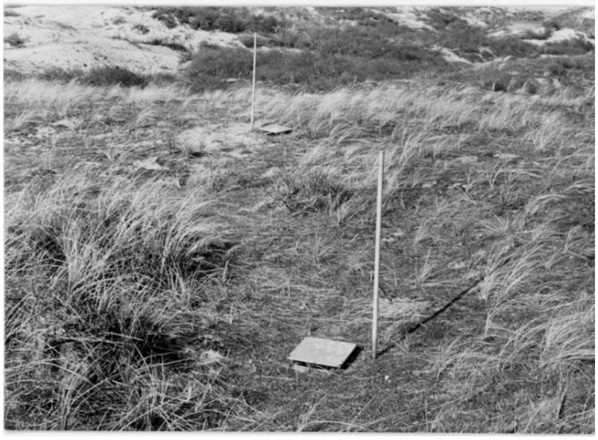

Pitfalls 6 and 5 in April 1955 with in the background pitfall 3

Pitfalls 6 and 5 in April 1955

Pitfalls 7-12 are located in the sea dunes about 150 meters from pitfalls 1-6. They are more inside in elongated East-West flat dune valley with little overgrowth of short moss and the like.

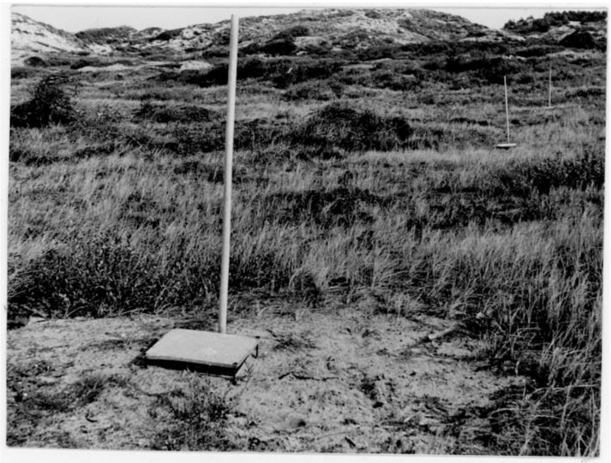

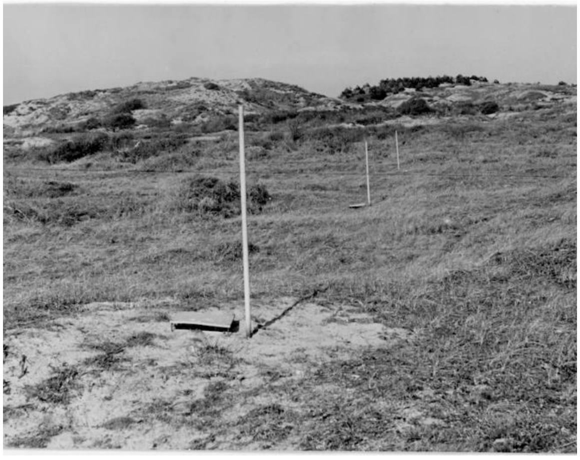

Pitfall 7 in April 1955 with in the background pitfalls 8 and 9

Pitfalls 7, 8 and 9 in April 1955

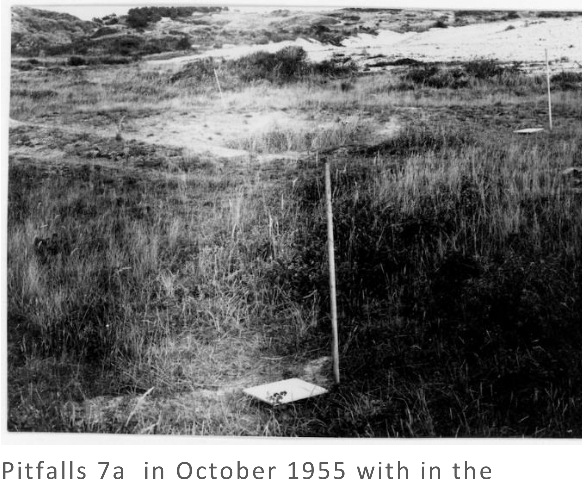

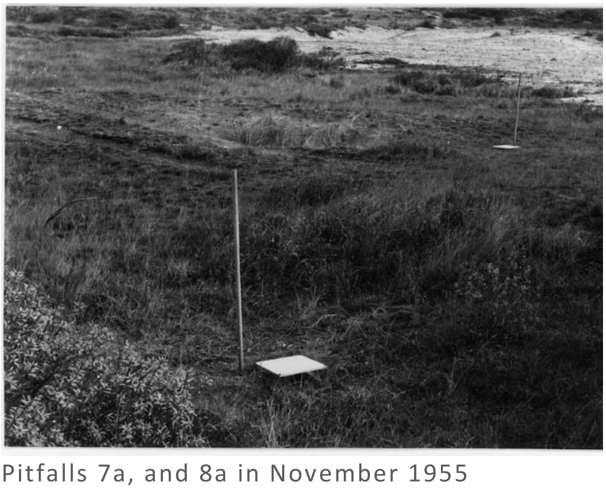

Pitfalls 7a in October 1955 with in the background 8a and 10a

Pitfalls 7a, and 8a in November 1955

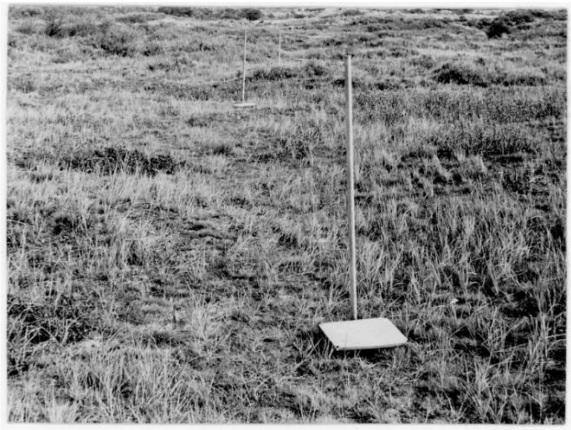

Pitfall 8 in April 1955 with in the background pitfalls 9 and 10

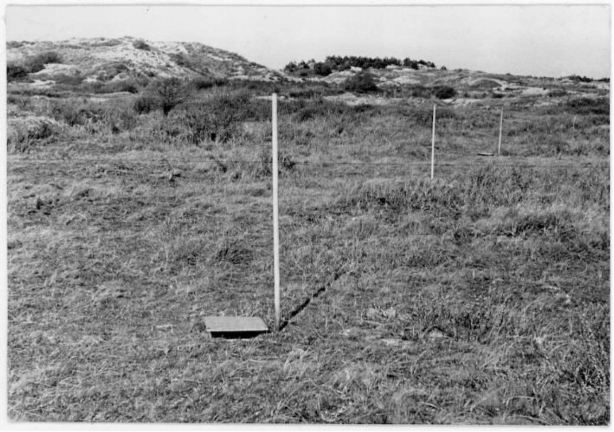

Pitfalls 9,10 and 11 in April 1955

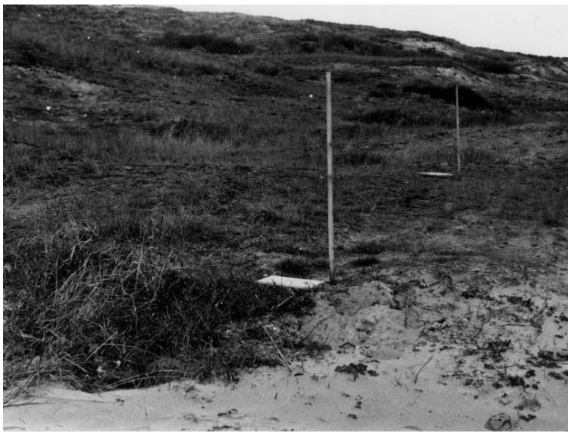

Pitfalls 8a and 9a in November 1955

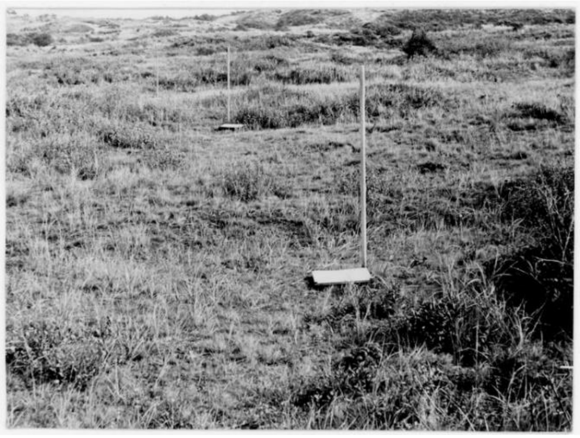

Pitfalls 10,11 and 12 in October 1954

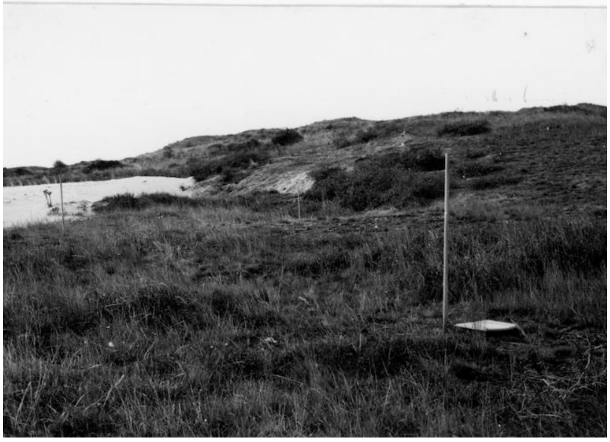

Pitfall 10a in November 1955 with in the background pitfalls 9a and 7a

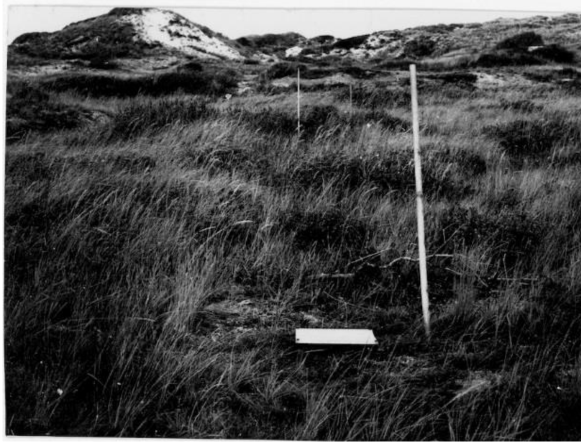

Pitfall 10a in October 1955 with in the background pitfalls 11a and 12a

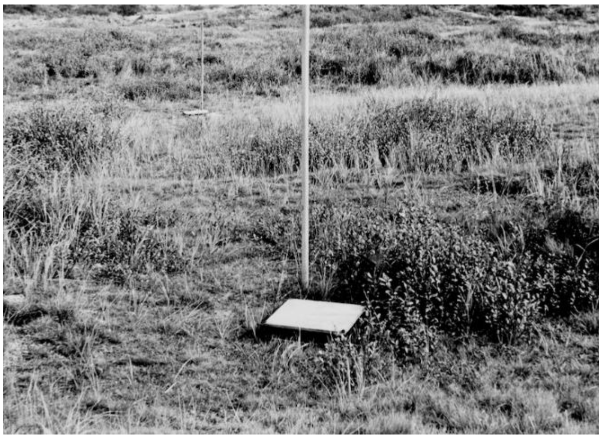

Pitfalls 11 and 12 in October 1954

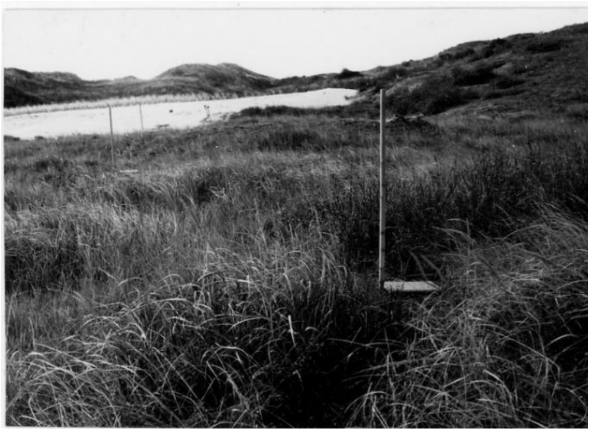

Pitfall 11a in November 1955 with in the background 10a and 9a

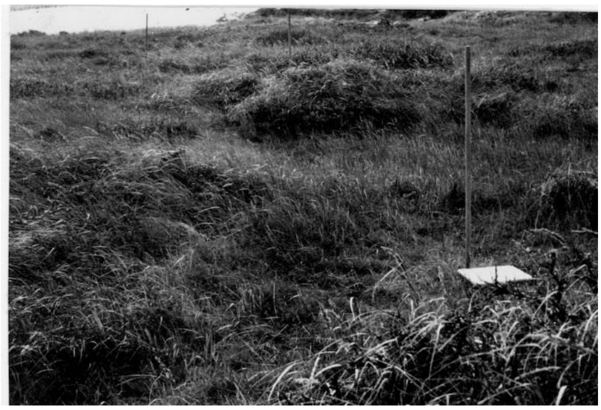

Pitfall 12a in October 1955 with in the background 11a and 10a

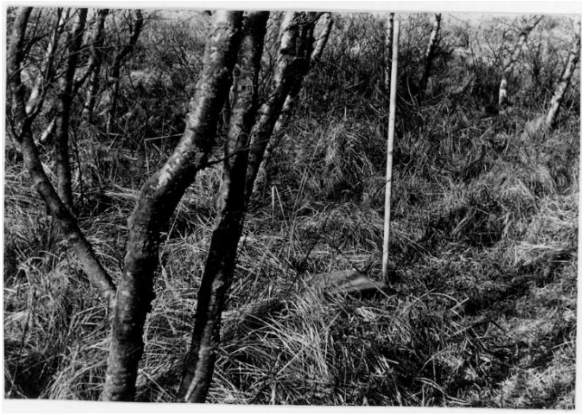

Pitfall 13 in April 1955

Pitfalls 13-15 are in the sea dunes about 250 meter northwest of the “Rijndelpad” in a grove of very low, dense birches.

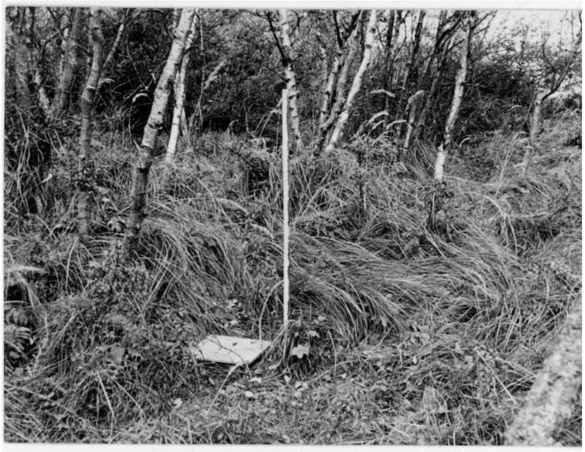

Pitfall 13 in October 1954

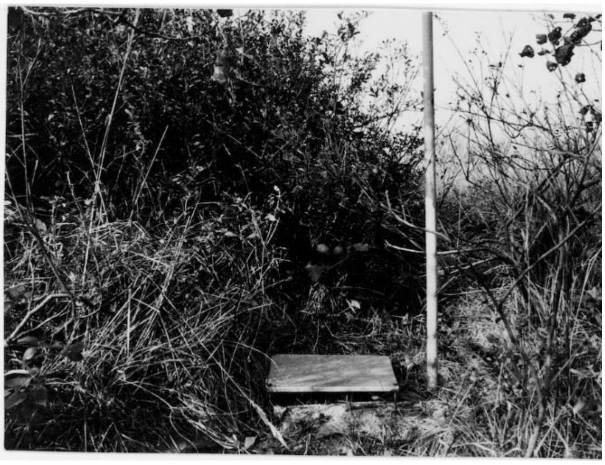

Pitfall 13a in October 1955

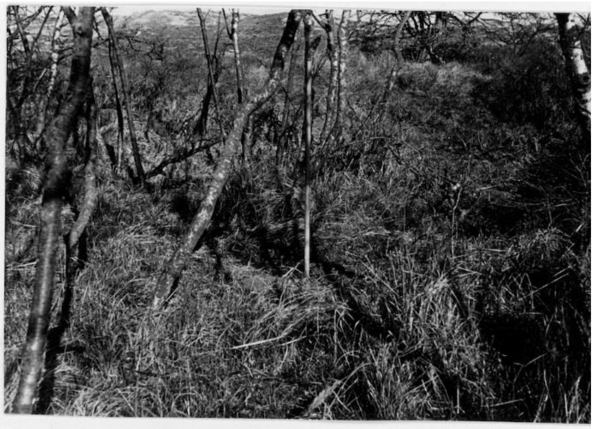

Pitfall 14 in April 1955

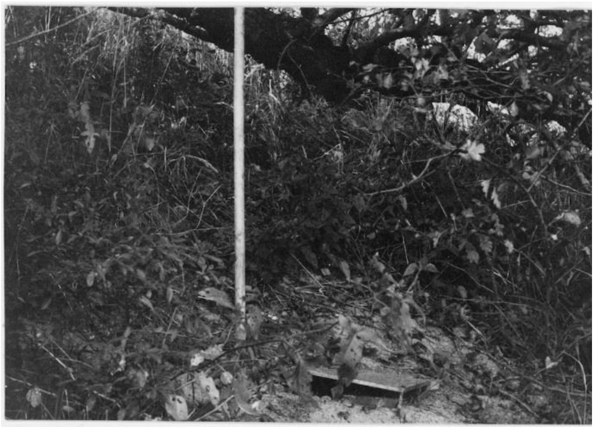

Pitfall 14a in October 1955

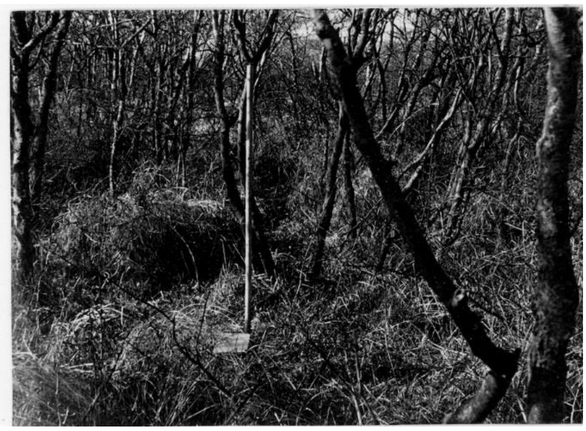

Pitfall 15 in April 1955

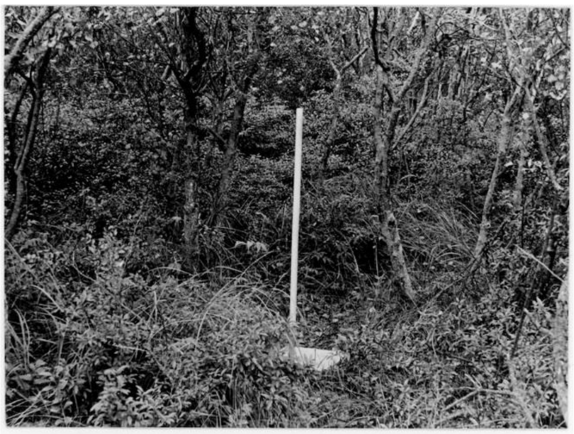

Pitfall 15 in October 1954

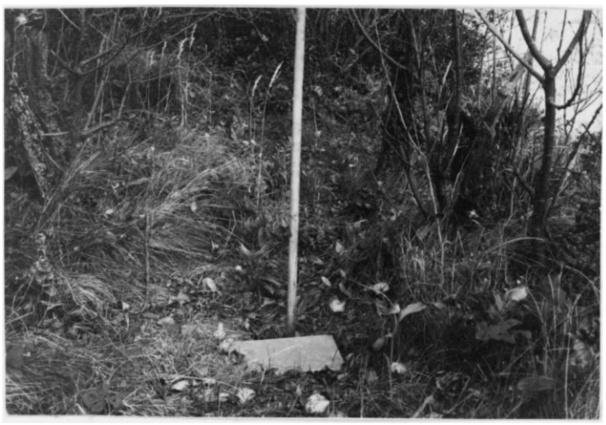

Pitfall 15a in October 1955

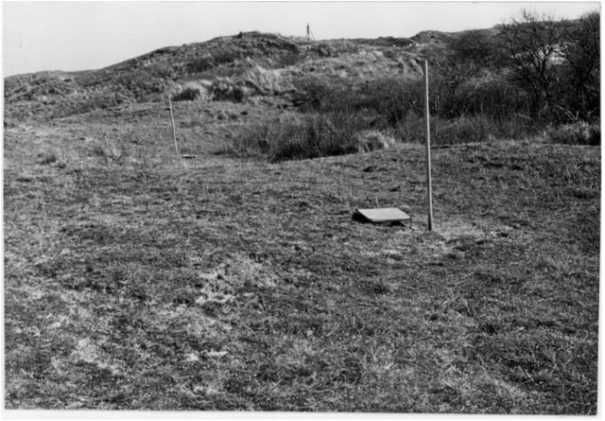

Pitfalls 16 and 17 in April 1955

Pitfalls 16-18 are in the sea dunes about 250 meter northwest of the “Rijndelpad” outside a grove of very low, dense birches.

Pitfalls 16a, 17a and 18a are placed by the slope in the sea dunes to the west of the lowland area.

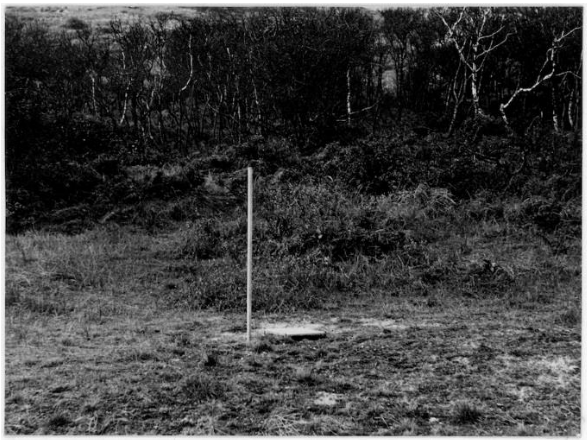

Pitfall 16 in October 1954

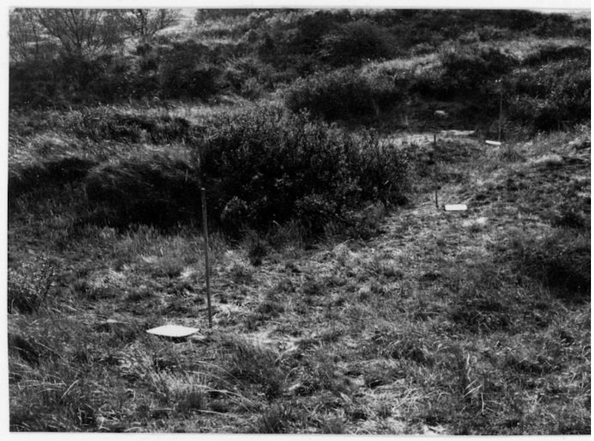

Pitfalls 16a, 17a and 18a in October 1955

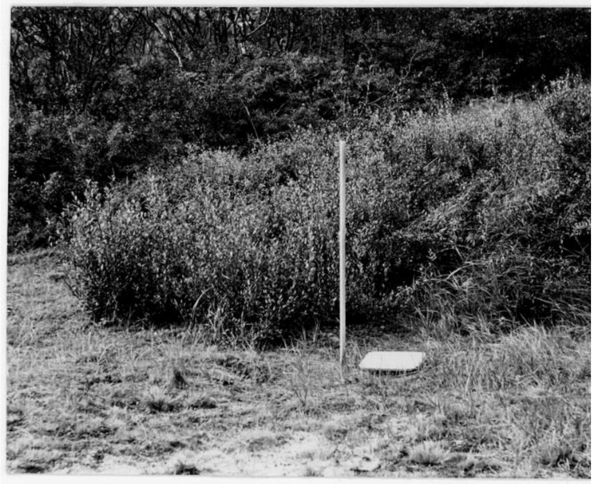

Pitfall 17 in October 1954

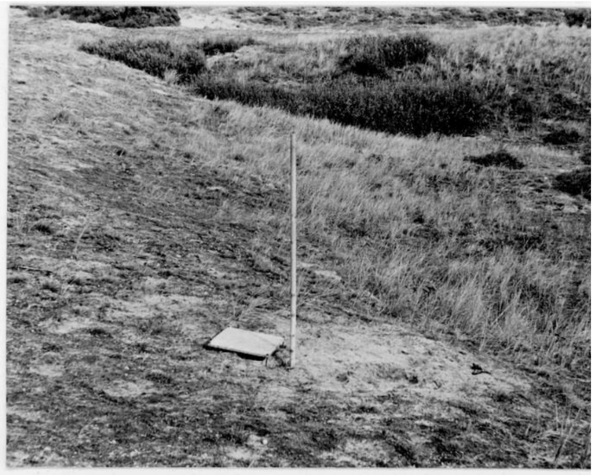

Pitfall 18 in October 1954

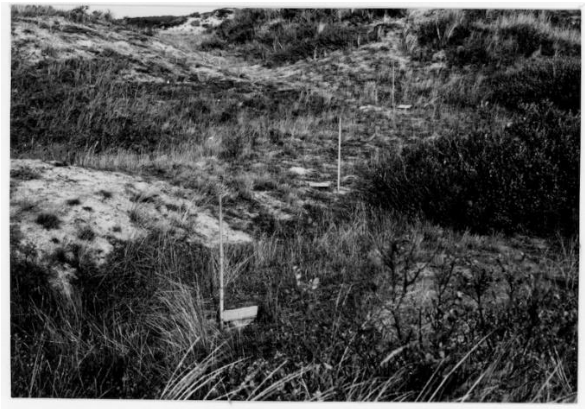

Pitfalls 18a, 17a and 16a in October 1955

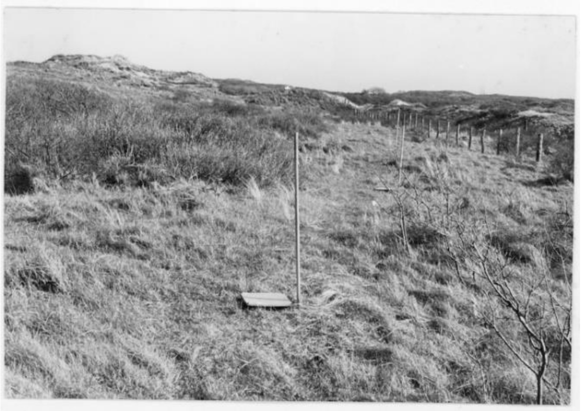

Pitfalls 19, 20 and 21 in April 1955

Pitfalls 19-21 are in the sea dunes about 200 meter from the cycle path (northwest) outside a forest of low birches.

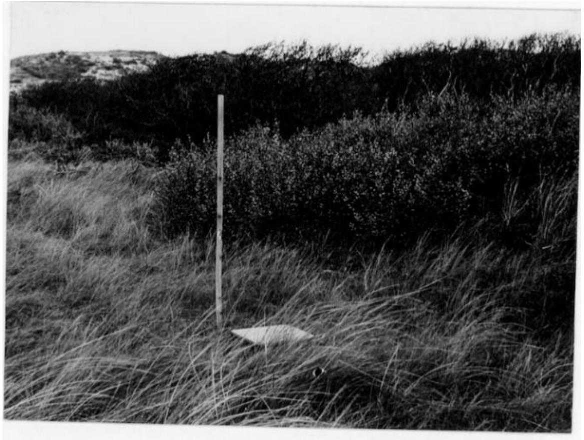

Pitfall 20 in October 1954

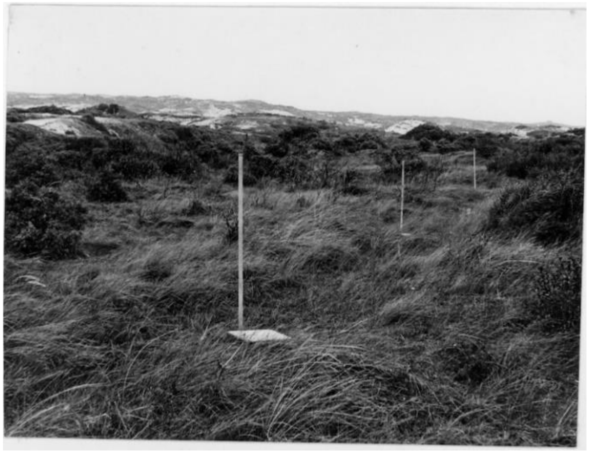

Pitfall 21, 20 and 19 in October 1954

Pitfalls 22-24 are also located in the sea dunes about 200 meter from the cycle path (northwest) in a forest of low birches.

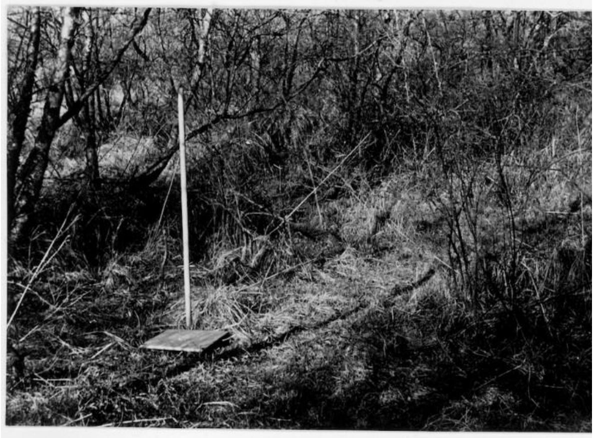

Pitfall 22 in April 1955

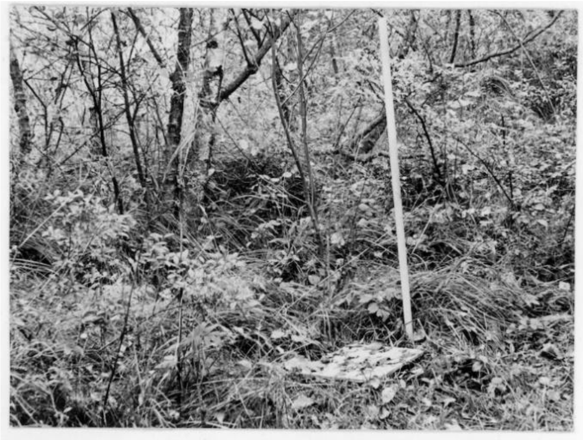

Pitfall 22 in October 1954

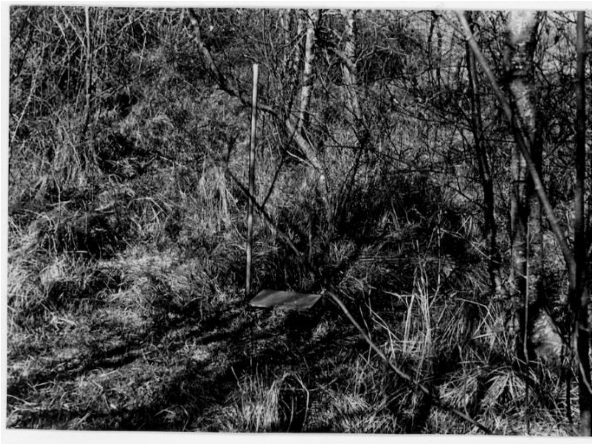

Pitfall 23 in April 1955

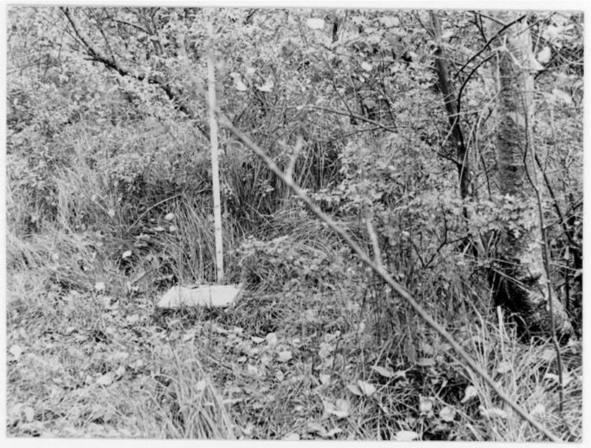

Pitfall 23 in October 1954

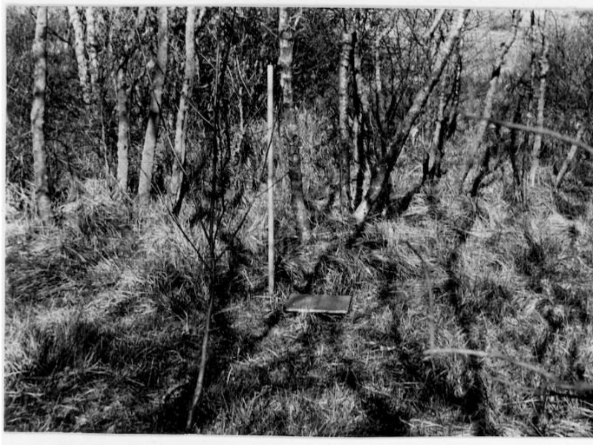

Pitfall 24 in April 1955

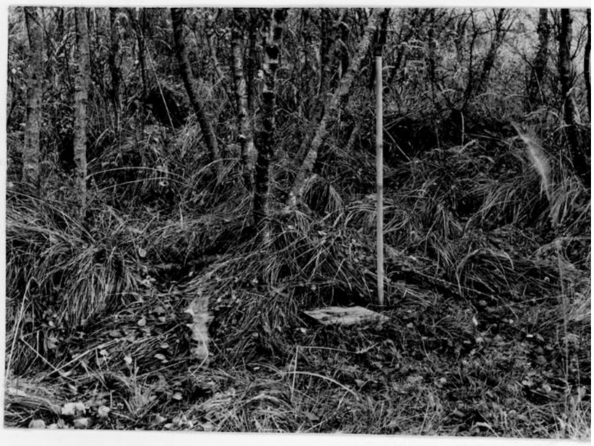

Pitfall 24 in November 1955

2. Rozenbos, pitfalls 76-100

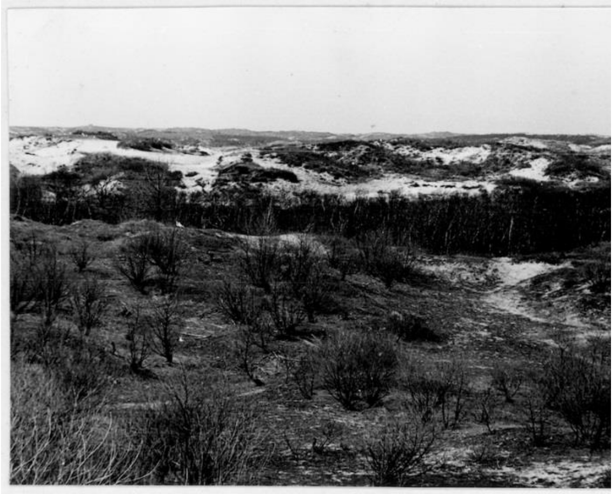
View on Rozenbos April 1955

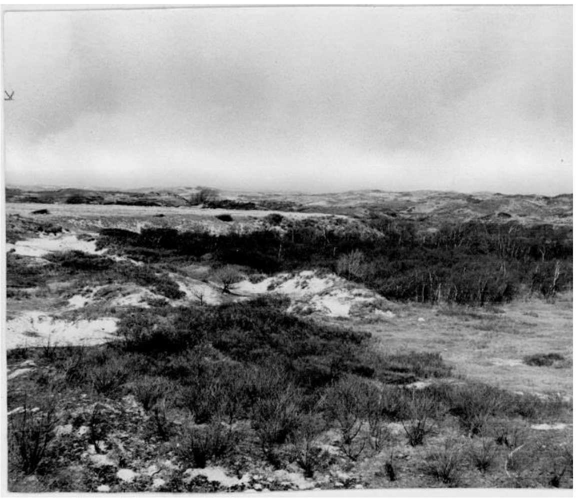
Rozenbos pitfalls 85-93 and 100 October 1955

pitfalls 76-78	open terrain with dense vegetation of dune reed (*Calamagrostis epigejos*), etc.
pitfalls79-81	birch forest
pitfalls 82-84	birch forest
pitfalls 85-87, 100	low overgrowth of mosses, lichens, etc.
pitfalls 88-90	open terrain with dense vegetation of dune reed (*Calamagrostis epigejos*), etc.
pitfalls 91-93	open terrain with dense vegetation of dune reed (*Calamagrostis epigejos*), etc.
pitfalls 94-96	birch forest
pitfalls 97-99	birch forest

Pitfalls 76-78 are located in the “Elleboogsprang” in Rozenbos. It is an open area in forest with some scattered birches and *Calamagrostis epigejos*.

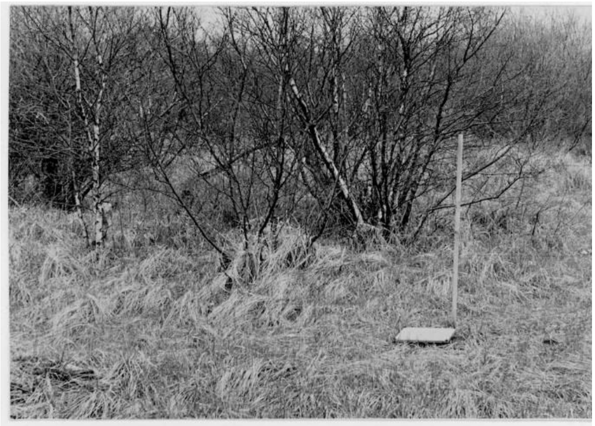
Pitfall 76 in April 1955

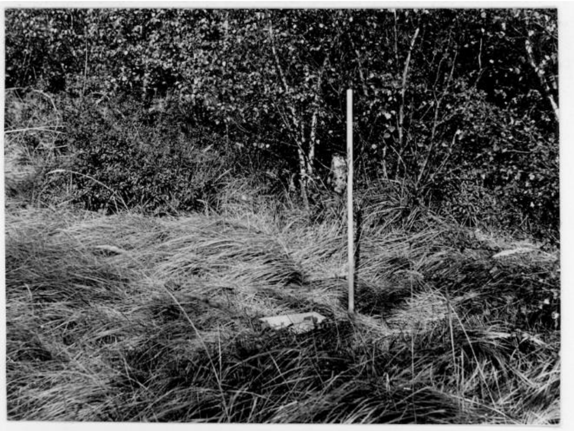
Pitfall 76 in October 1954

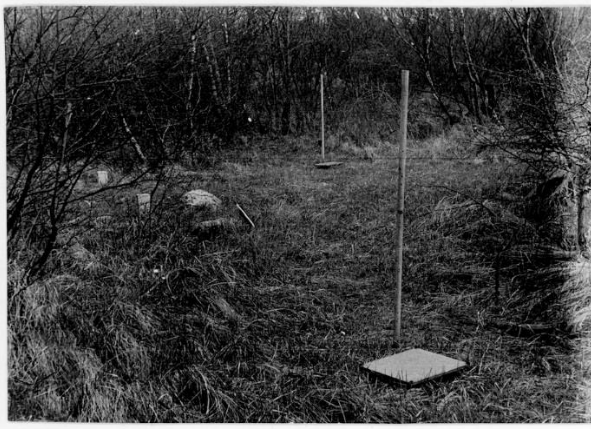
Pitfalls 76 and 78 in April 1955

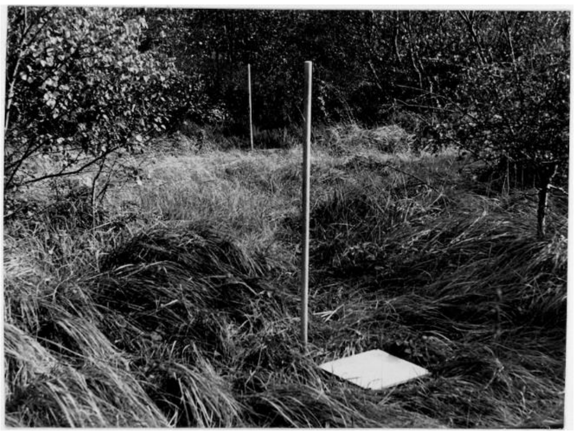
Pitfalls 76 and in October 1954

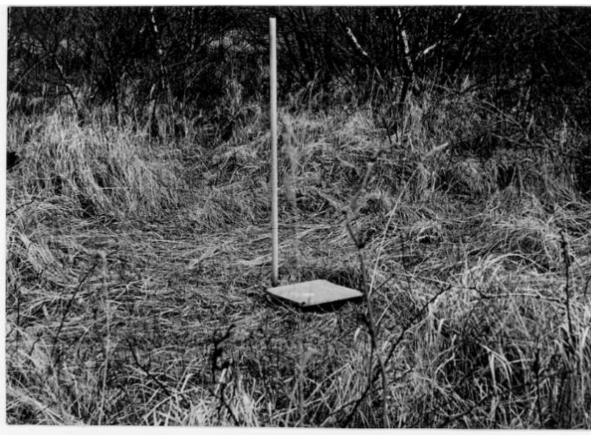
Pitfall 77 in April 1955

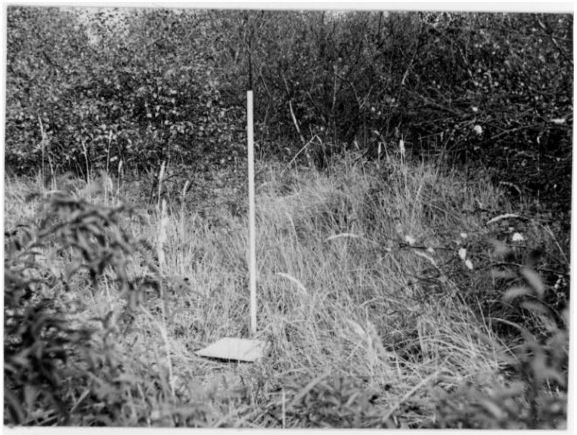
Pitfall 77 in October 1954

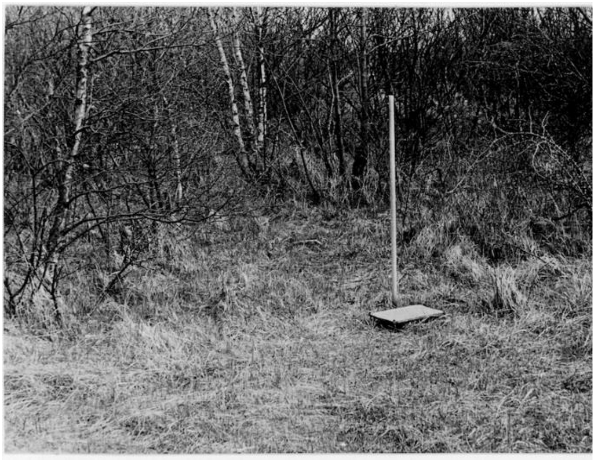
Pitfall 78 in April 1955

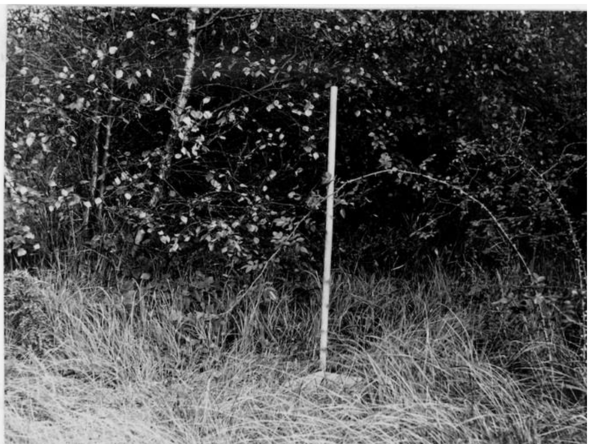
Pitfall 78 in October 1954

Pitfalls 79-81 are located in the “Elleboogsprang” in the Rozenbos, consisting of birches with a moderate amount of litter.

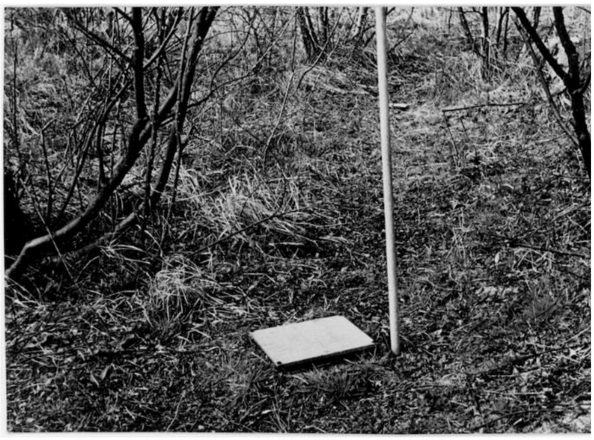

Pitfall 79 in April 1955

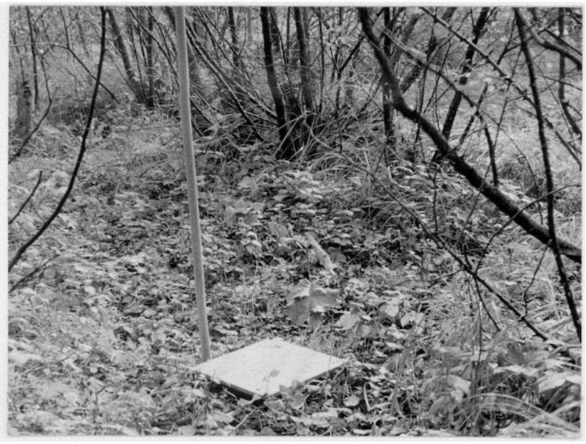

Pitfall 79 in October 1954

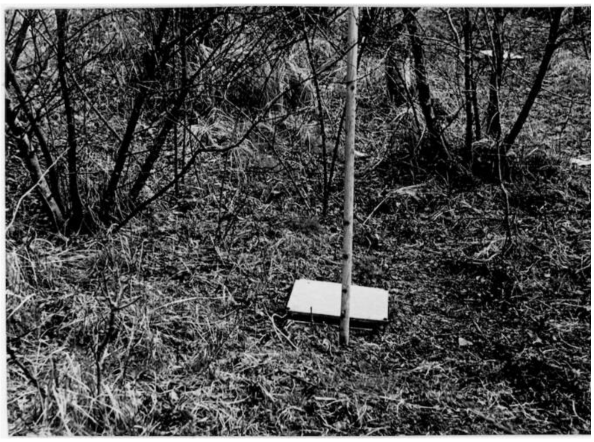

Pitfall 80 in April 1955

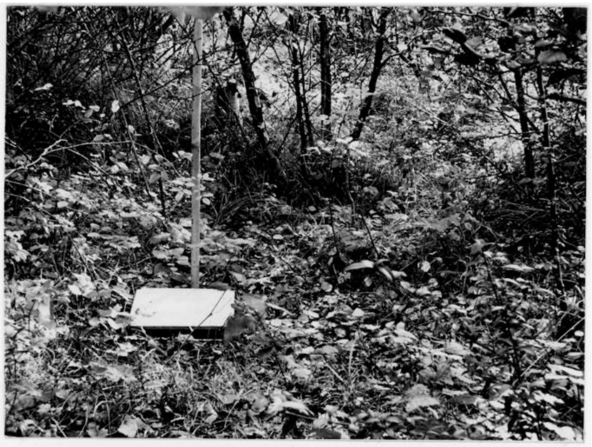

Pitfall 80 in October 1954

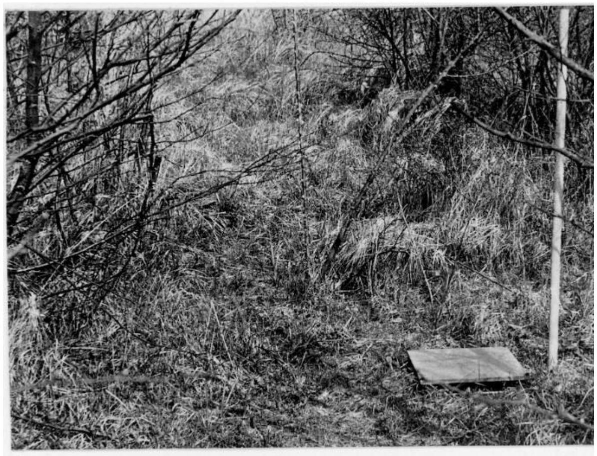

Pitfall 81 in April 1955

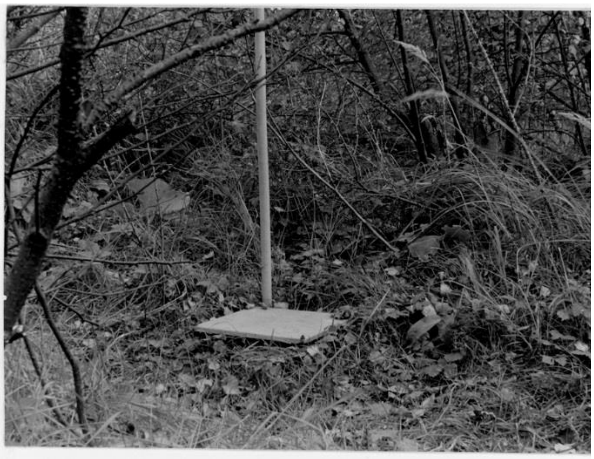

Pitfall 81 in October 1954

Pitfall 79 in [illegible]

Pitfalls 82-84 are located in the “Elleboogsprang” in het Rozenboa in a dense forest of young birches with a lot of litter.

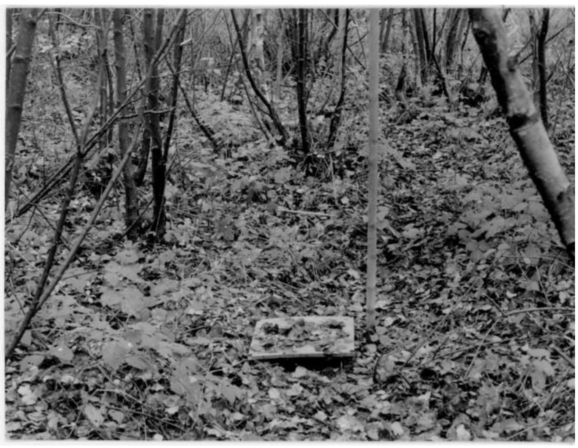
Pitfall 82 in November 1955

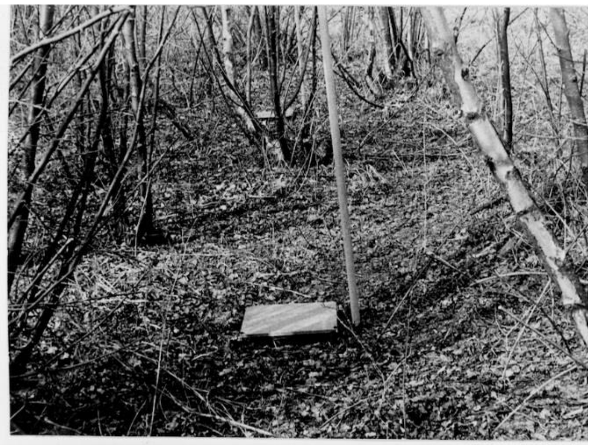
Pitfalls 82 and 83 in April 1955

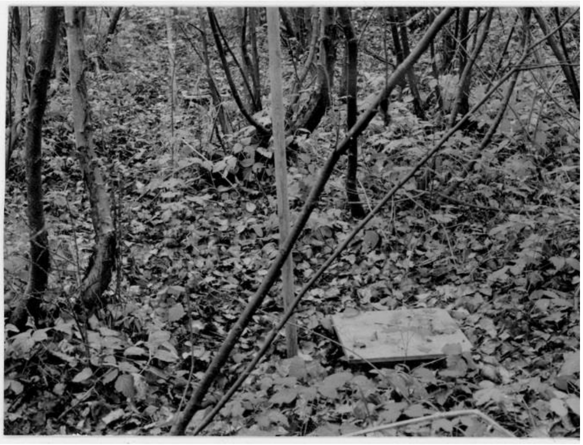
Pitfalls 83 and 82 in November 1955

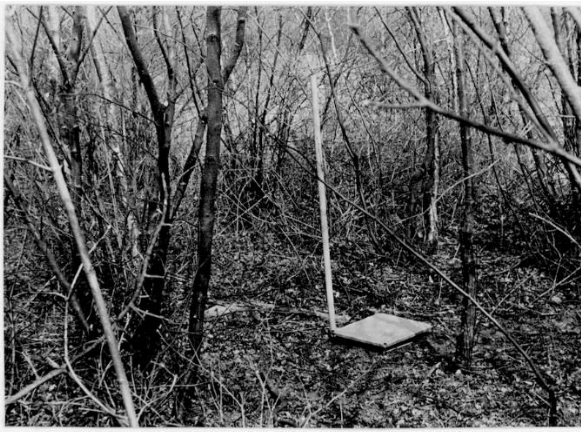
Pitfall 84 in April 1955

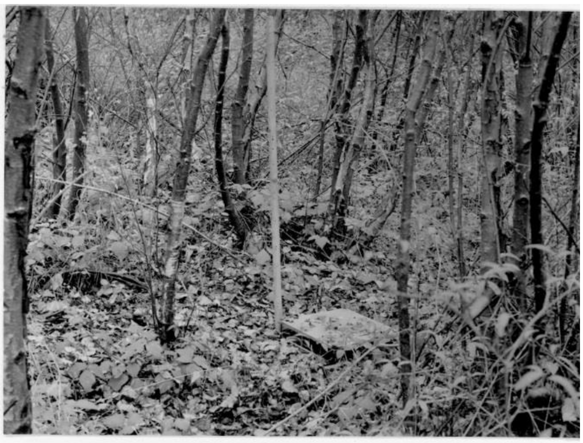
Pitfall 84 in November 1955

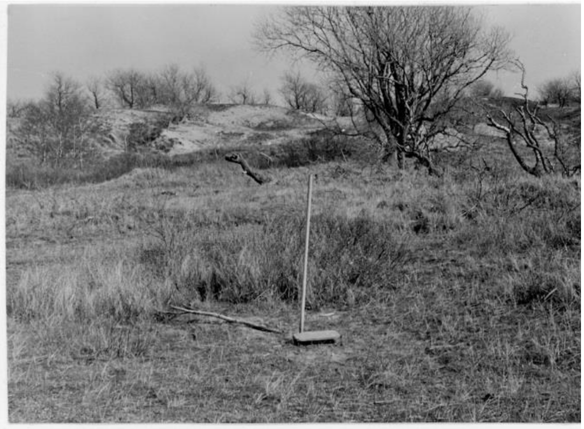
Pitfall 85 in April 1955

Pitfalls 85-87 are located in the “Elleboogsprang” outside the Rozenbos (South) in plain with mosses, lichens and grasses.

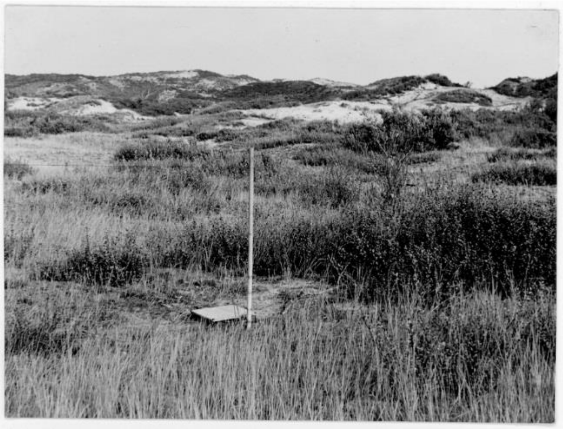

Pitfall 85 in October 1955

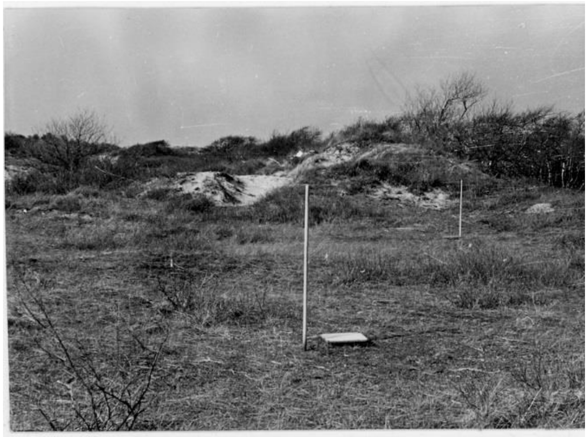

Pitfalls 86 in April 1955 with in the background pitfall 87

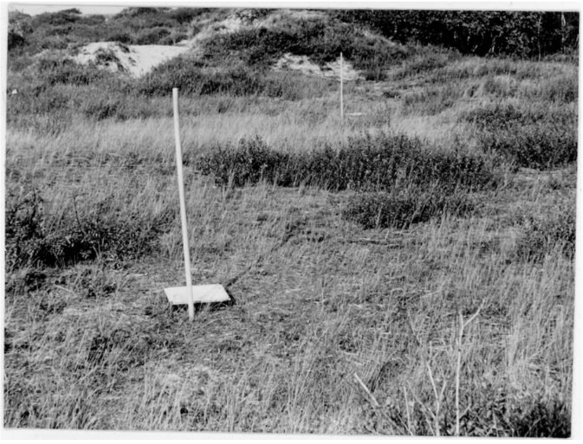

Pitfall 86 in October 1954 with in the background pitfall 87

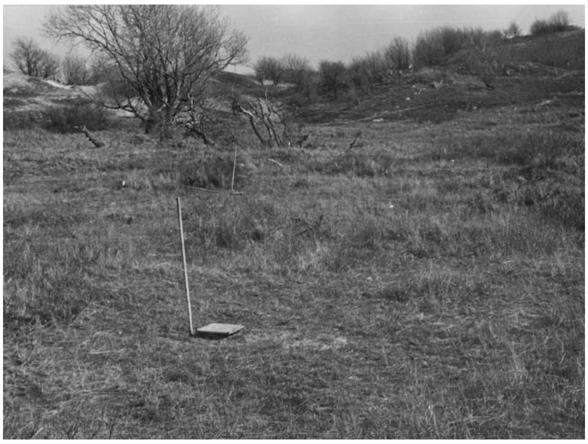

Pitfalls 87 and 85 in April 1955

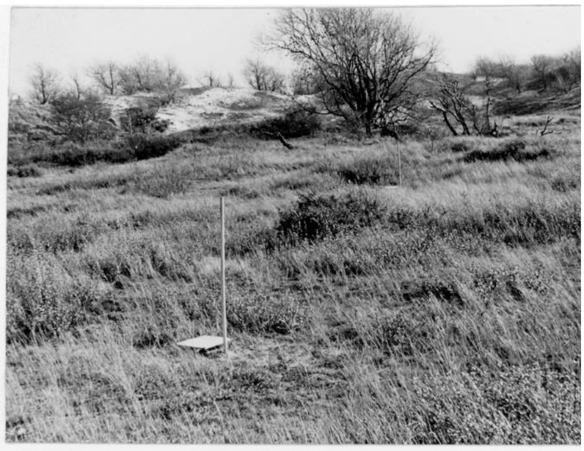

Pitfalls 87 and 85 in October 1954

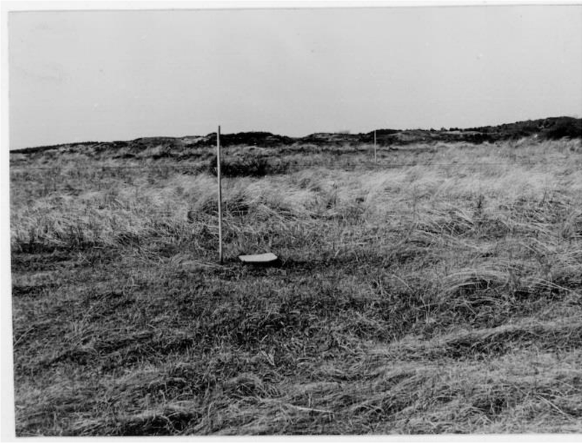

Pitfalls 88 and 89 in April 1955

Pitfalls 88-93 are located in the “Elleboogsprang” on the plain at about 16 meter above N.A.P., overgrown with beach grass (*Ammophila arenaria*) and dune reed (*Calamagrostis epigejos*).

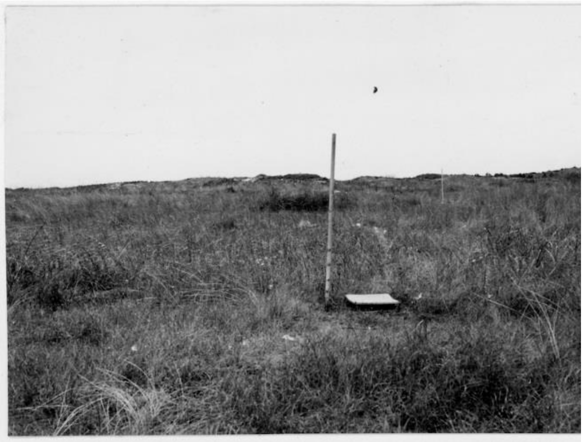

Pitfalls 88 and 89 in November 1955

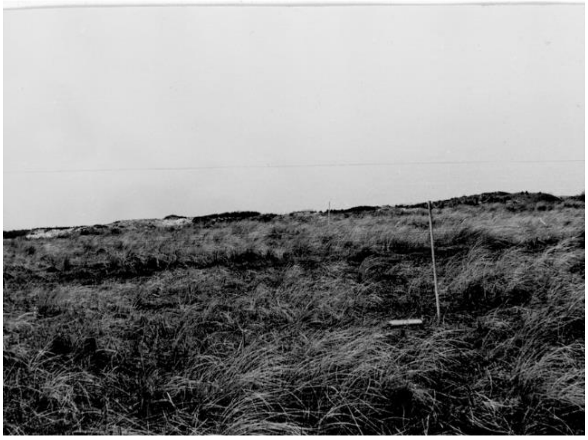

Pitfall 91 in April 1955 with in the background pitfall 90

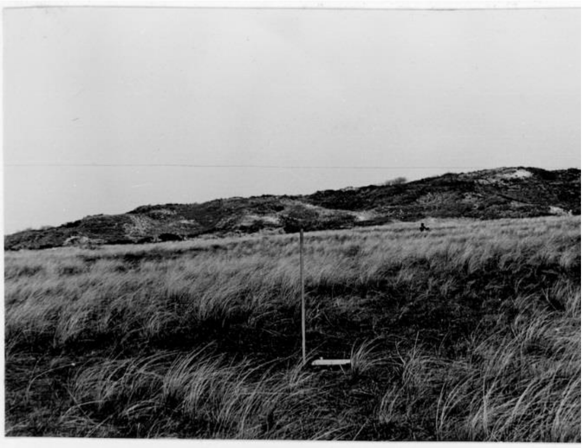

Pitfall 92 in April 1955 with in the background pitfalls 91 and 88

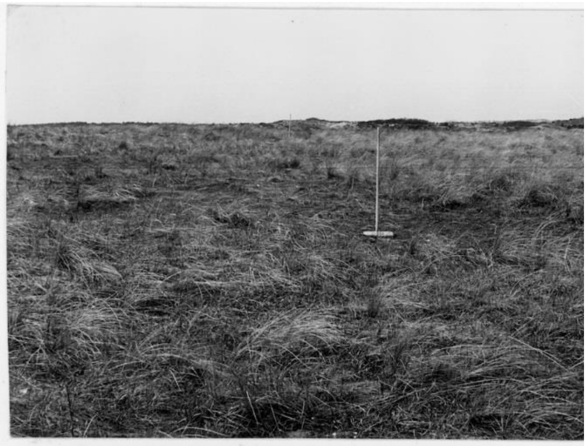

Pitfalls 92 and 93 in April 1955

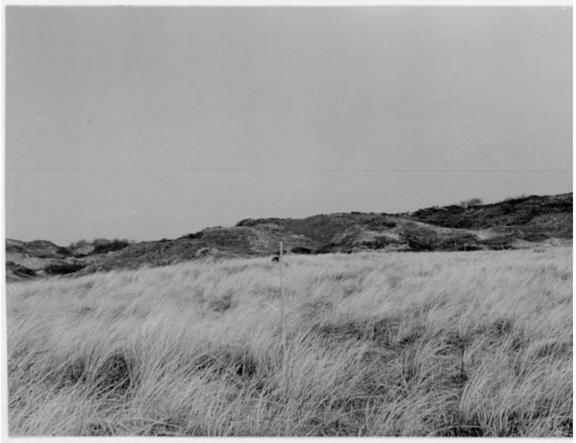

Pitfall 93 in April 1955 with in the background pitfalls 90 and 89

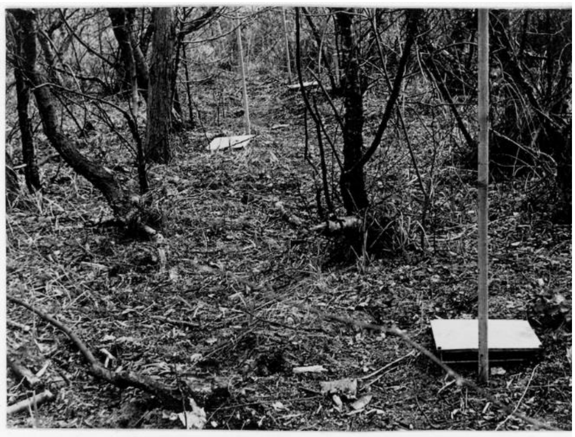

Pitfall 94 in April 1955 with in the background pitfalls 95 and 96

Pitfalls 94-96 are located in the “Elleboogsprang” in the Rozenbos in the middle of a homogeneous birch forest in a bowl-shaped valley with quite a lot of litter.

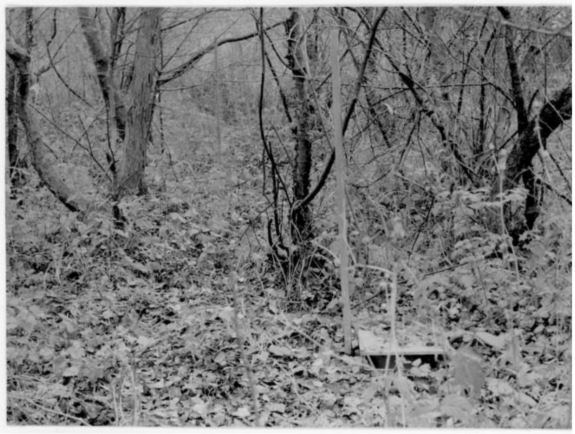

Pitfall 94 in November 1955 with in the background pitfalls 95 and 96

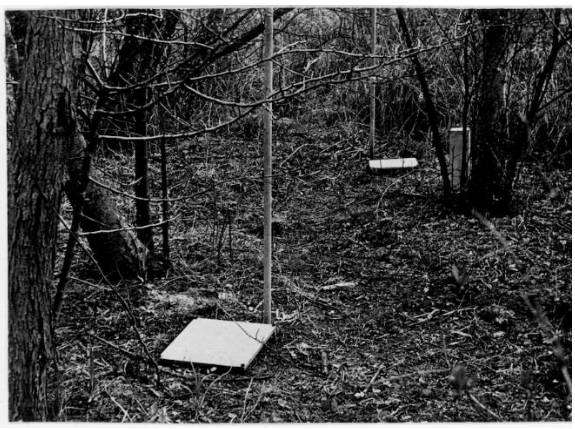

Pitfalls 95 and 96 in April 1955

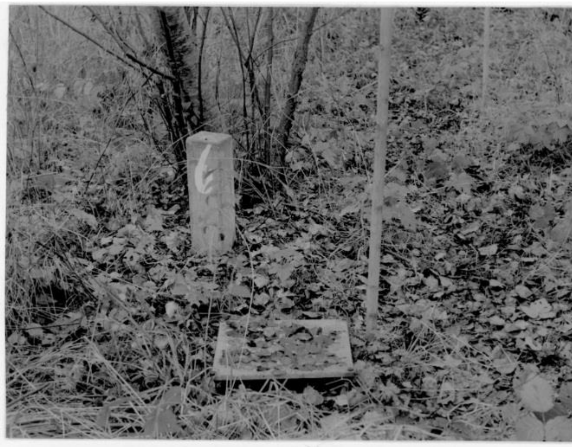

Pitfall 96 in November 1955 with in the background pitfalls 95 and 94

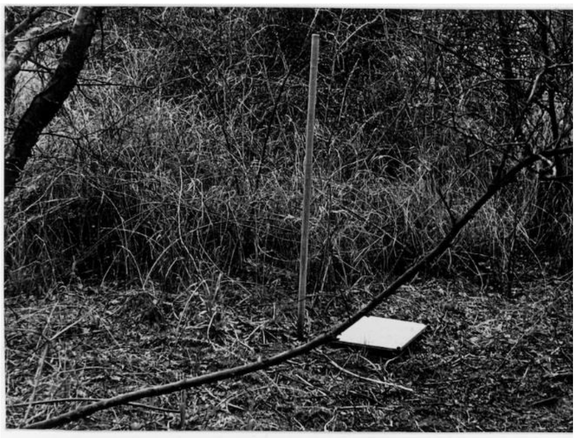

Pitfall 96 in April 1955

Pitfalls 97-99 are located in the “Elleboogsprang” in the Rozenbos in the plain south of the terrain where pitfalls 85-87 are.

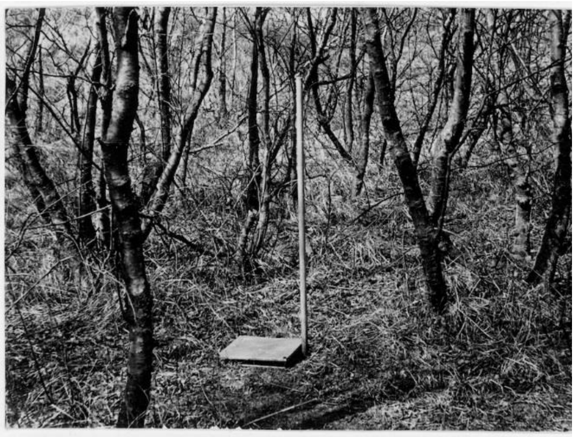

Pitfall 97 in April 1955

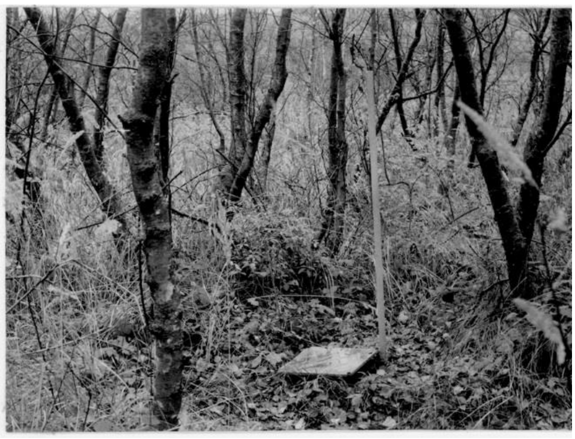

Pitfall 97 in November 1955

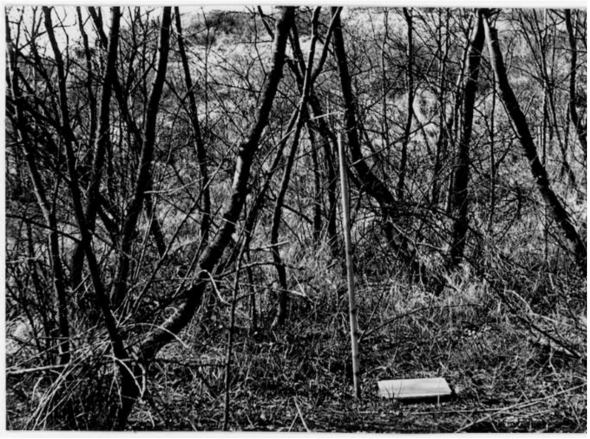

Pitfall 98 in April 1955

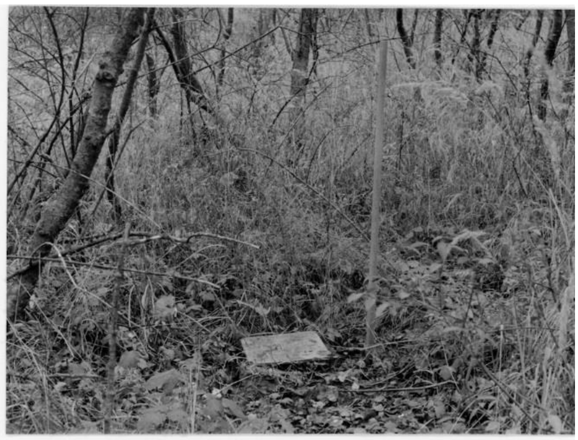

Pitfall 98 in November 1955

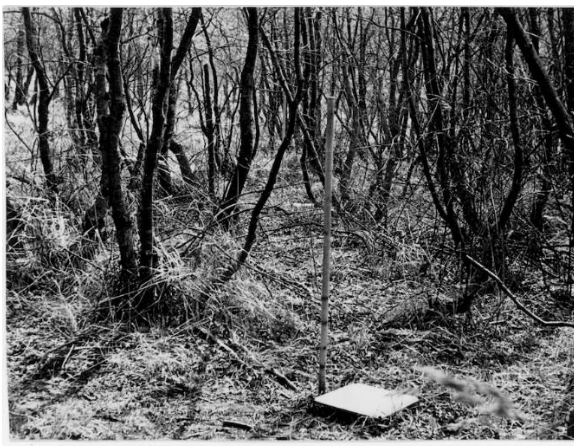

Pitfall 99 in April 1955

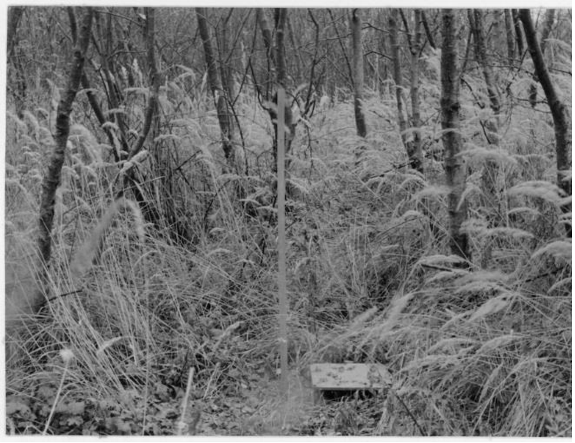

Pitfall 99 in November 1955

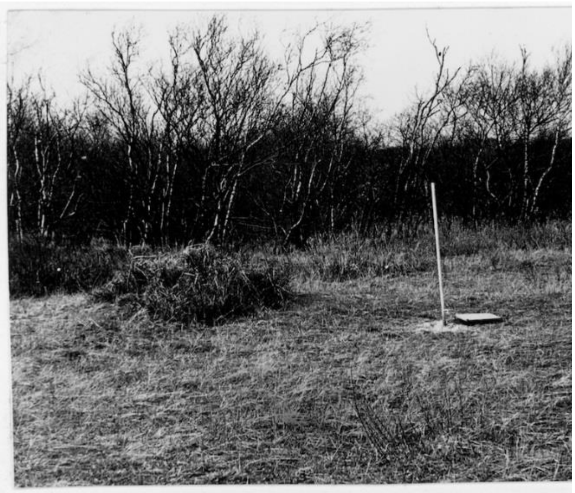

Pitfall 100 in April 1955

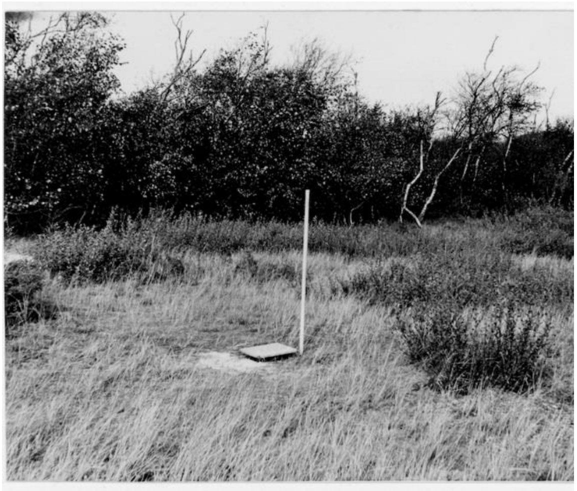

Pitfall 100 in October 1954

3. Natte sprang, pitfalls 61-75

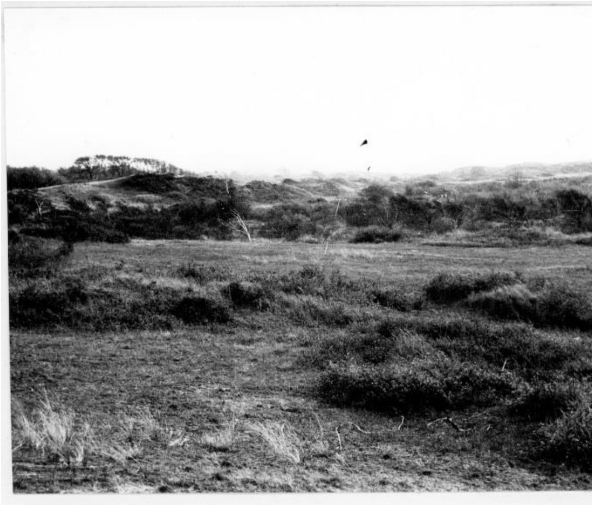

View on the Natte Sprang pitfalls 61-63
October 1955

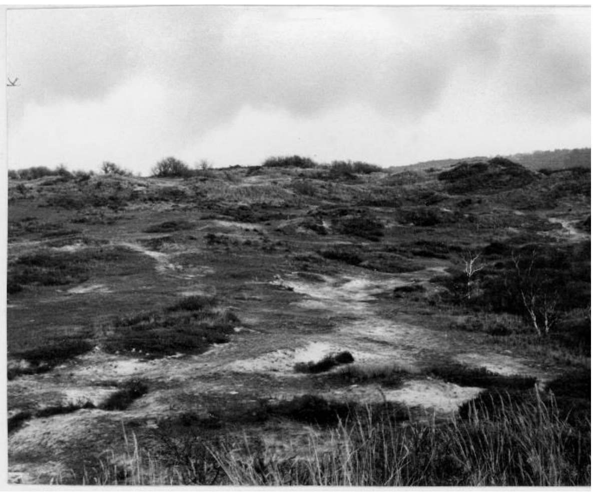

View on the Natte Sprang pitfalls 61-69
October 1955

pitfalls 61-63	low overgrowth of mosses, lichens, etc.
pitfalls 64-66	bare sand with little or no vegetation
pitfalls 67-69	scattered birches with mainly dune reed (*Calamagrostis epigejos*) as undergrowth
pitfalls 70-72	creeping willow (*Salix repens*) bushes
pitfalls 73-75	forest of black poplar (*Populus nigra*)

Pitfalls 61-63 are located in the dunes north east of the “Natte Sprang” in a plain with very low vegetation, almost exclusively mosses and lichens.

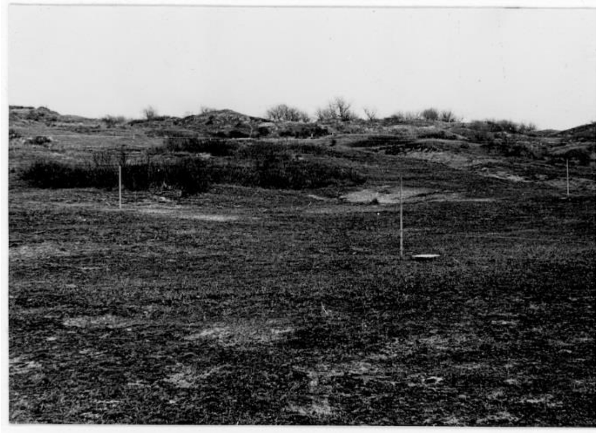

Pitfalls 61, 62 and 63 in April 1955

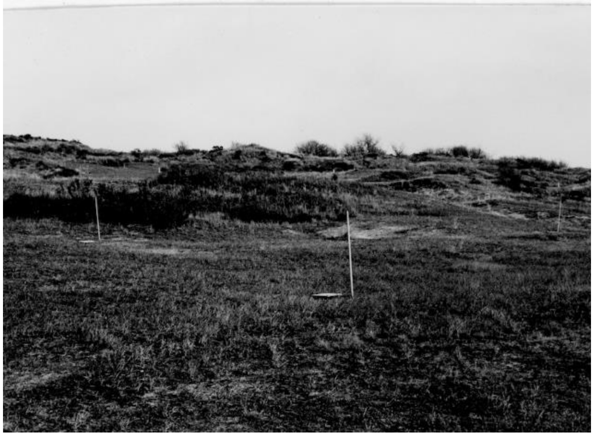

Pitfalls 61, 62 and 63 in October 1955

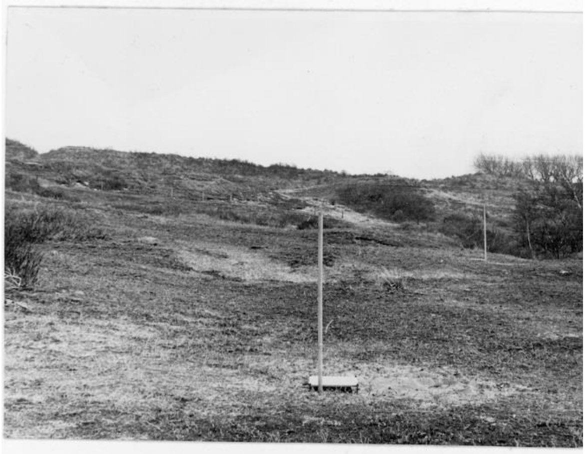

Pitfalls 62 and 63 in April 1955

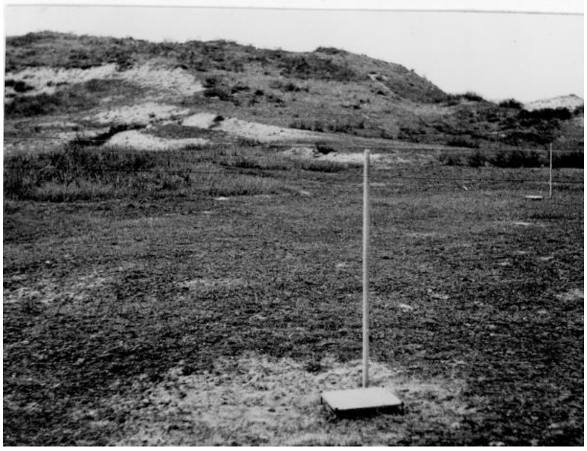

Pitfall 63 in April 1955 with pitfall 61 in the background

Pitfalls 64-66 are located in the dunes north east of the “Natte Sprang” in bare sand with a sparse vegetation of some mosses.

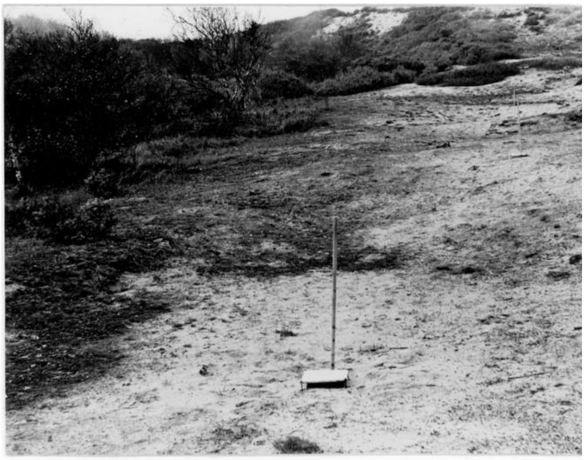

Pitfall 64 in October 1954 with pitfalls 65 and 66 in the background

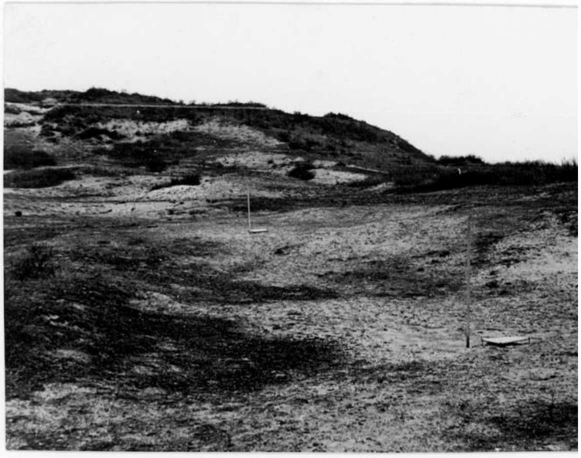

Pitfalls 64, 65 and 66 in April 1955

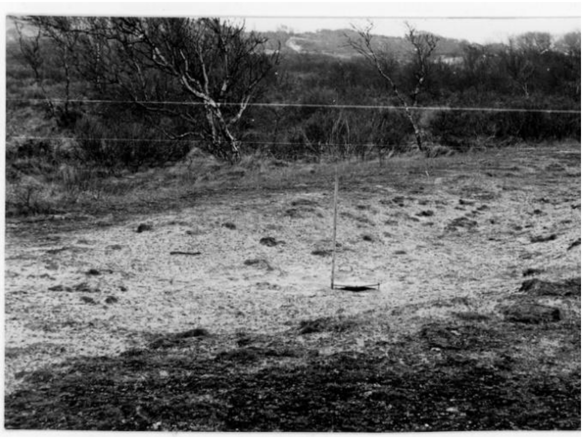

Pitfall 66 in April 1955

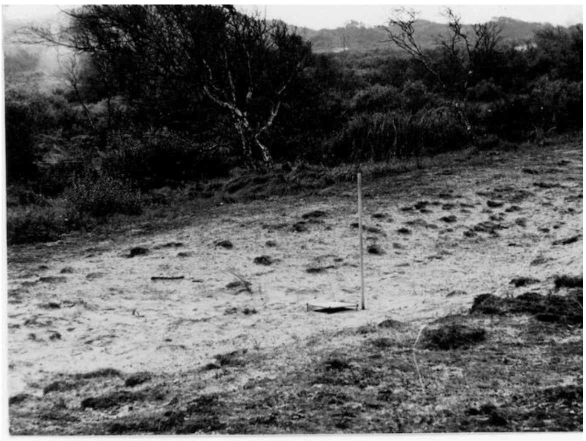

Pitfall 66 in October 1954

Pitfalls 67-69 are located in the dunes north east of the “Natte sprang” in grass, especially dune reed *Calamagrostis epigejos* under scattered birches and hawthorns (*Hippophae rhamnoides*).

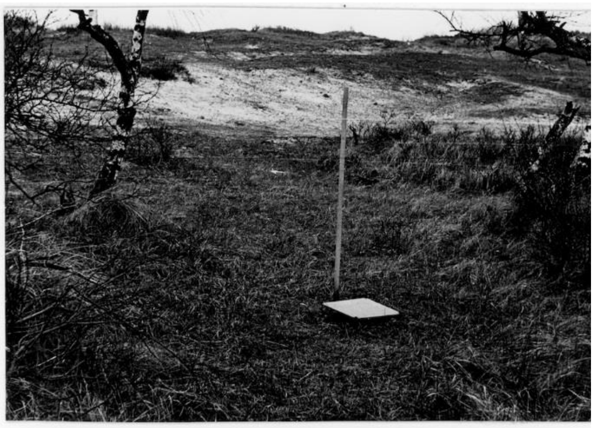
Pitfall 67 in April 1955

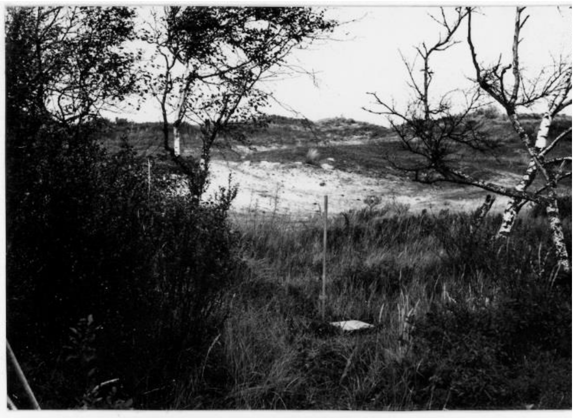
Pitfall 67 in October 1955

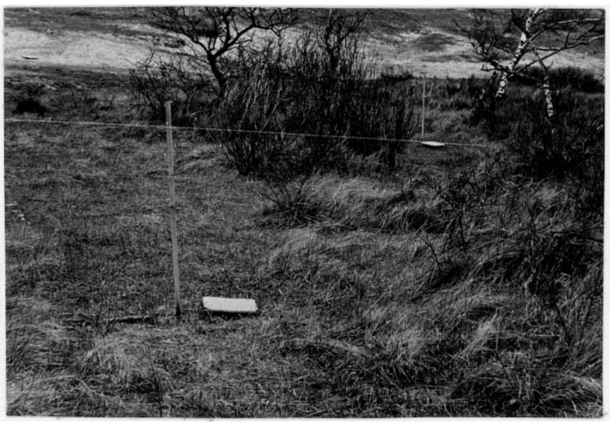
Pitfalls 68 in April 1955 with in the background pitfall 67

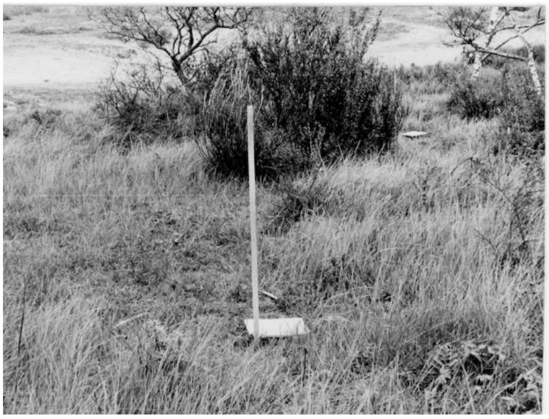
Pitfalls 68 and 67 in October 1955

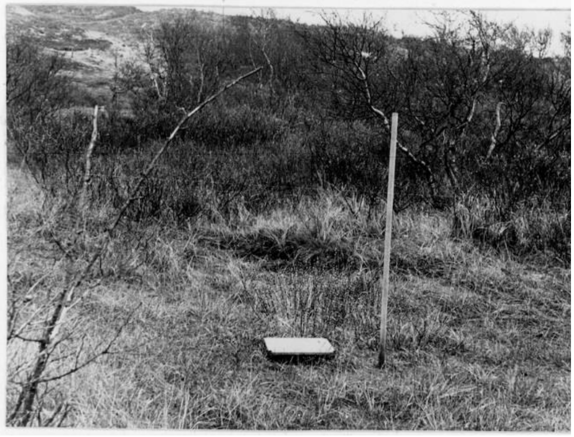
Pitfall 68 in April 1955

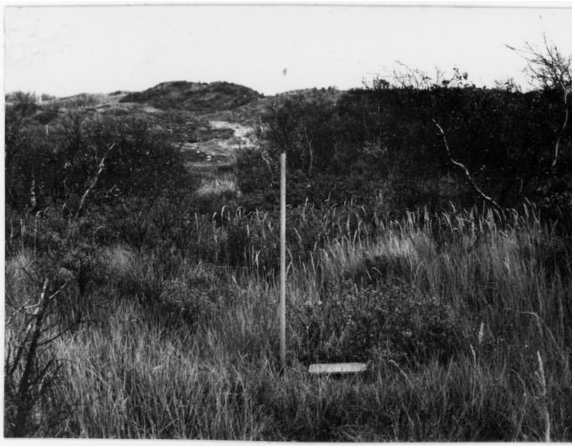
Pitfall 69 in October 1955

Pitfalls 70-72 are located in the dunes north east of the “Natte Sprang” in creeping willow groves (*Salix repens*)

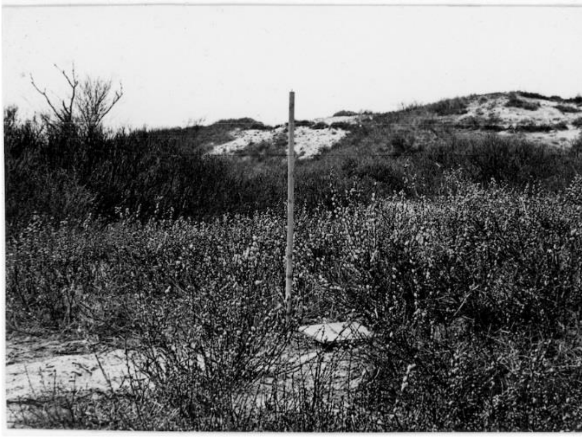

Pitfall 70 in April 1955

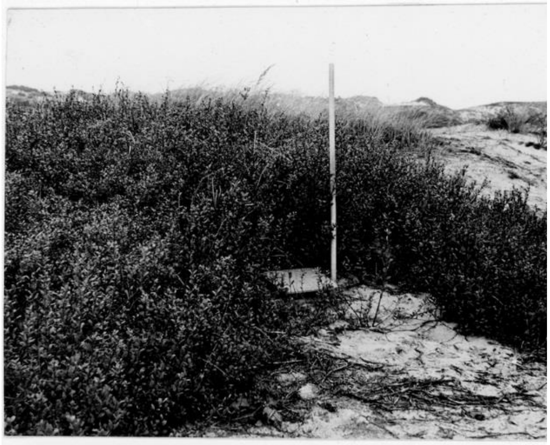

Pitfall 70 in October 1954

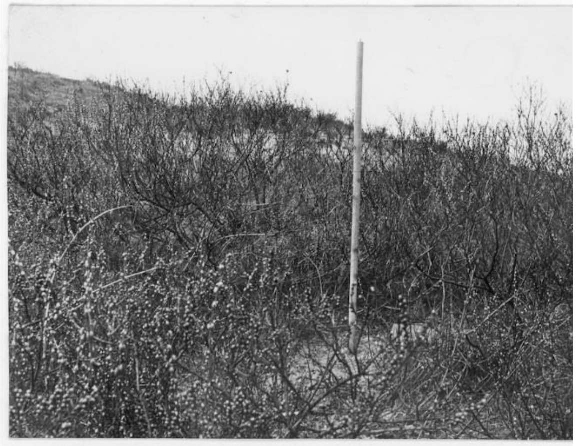

Pitfall 71 in April 1955

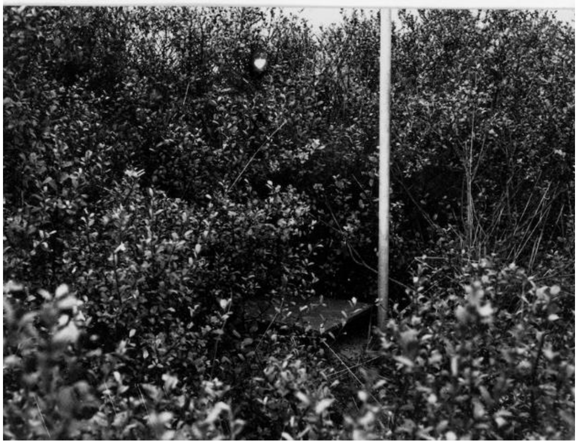

Pitfall 71 in October 1954

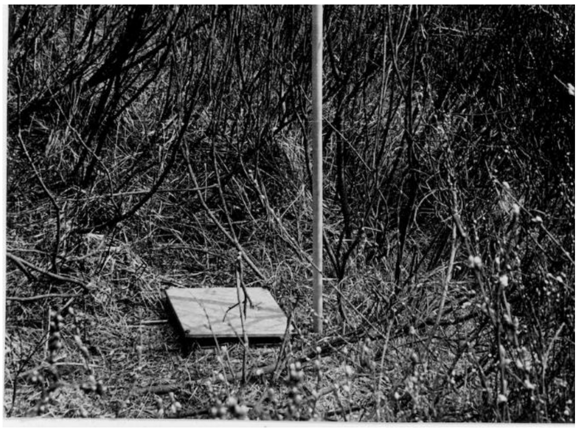

Pitfall 72 in April 1955

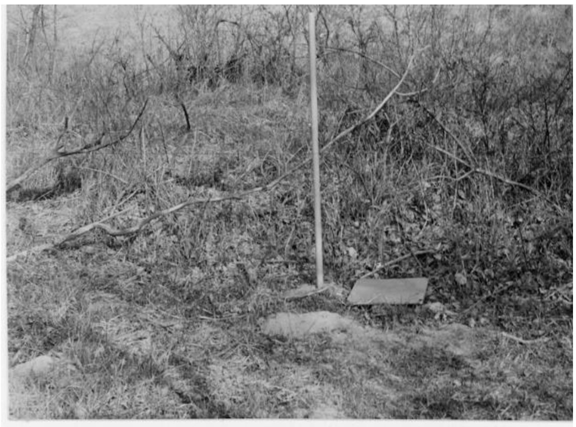

Pitfall 73 in April 1955

Pitfalls 73-75 are located in the dunes north east of the “Natte Sprang” inside a bunch of some old specimens of *Populus nigra* on a south eastern slope, with a lot of litter.

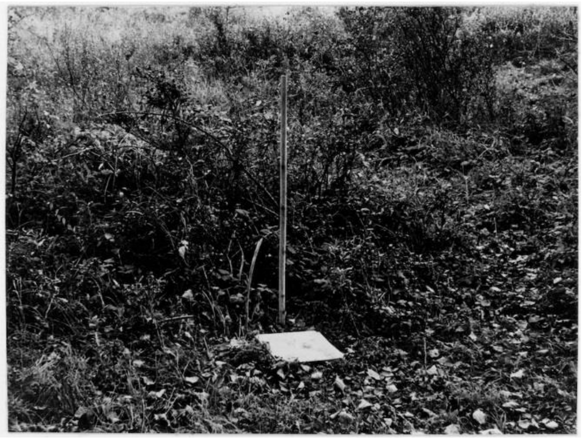

Pitfall 73 in October 1954

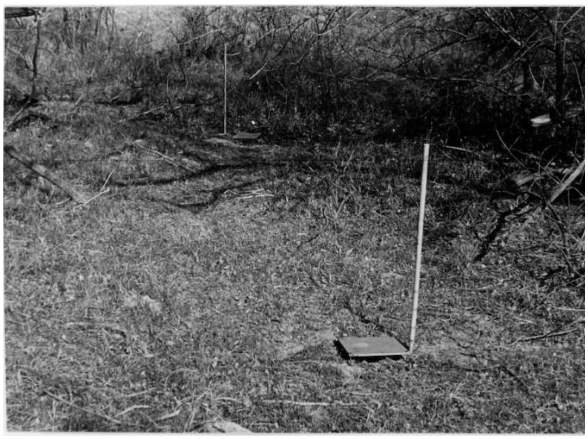

Pitfalls 73 and 74 in April 1955

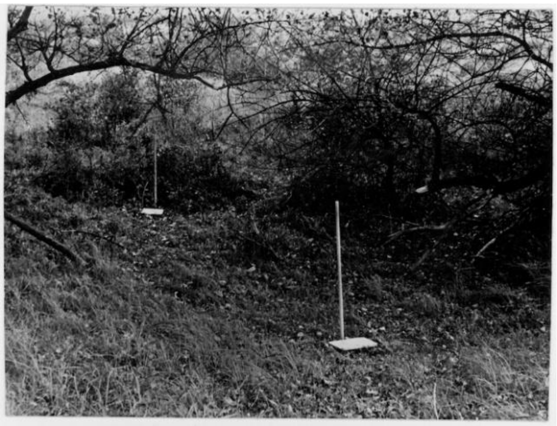

Pitfalls 73 and 74 in October 1954

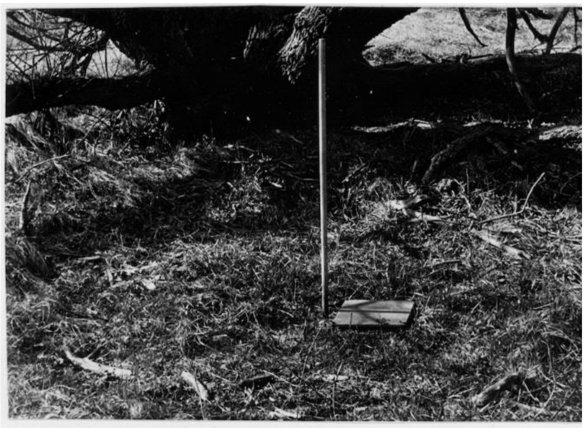

Pitfall 75 in April 1955

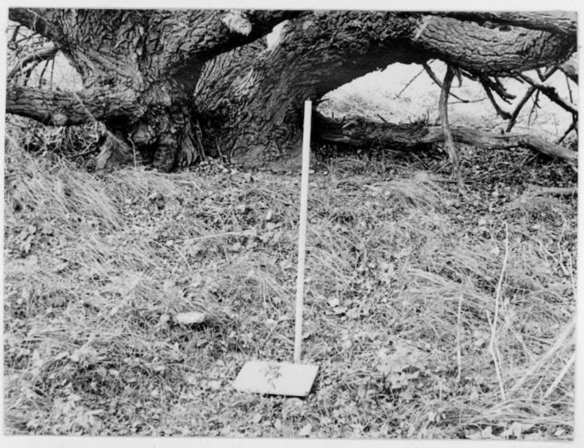

Pitfall 75 in October 1954

4. Bierlap, pitfalls 25-60

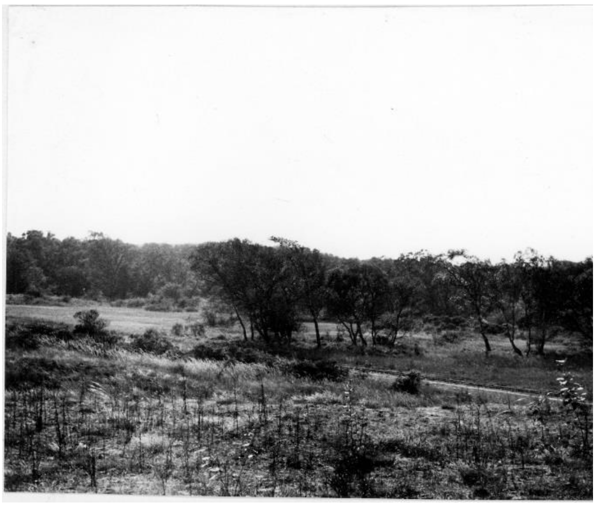

View on the Bierlap from pitfalls 25-30

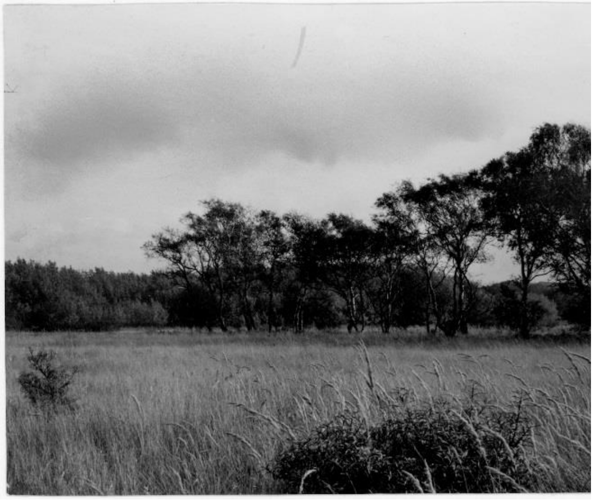

View on the Bierlap from pitfalls 31-36

pitfalls 25-27	open terrain with light beach grass (*Ammophila arenaria*) vegetation
pitfalls 28-30	bare sand with little or no vegetation
pitfalls 31-33	forest of aspen (*Populus tremulus*)
pitfalls 34-36	open terrain with dense vegetation of dune reed (*Calamagrostis epigejos*), etc.
pitfalls 37-39	scattered birches with mainly dune reed (*Calamagrostis epigejos*) as undergrowth
pitfalls 40-42	sea buckthorn (*Hippophae rhamnoides*) thicket
pitfalls 43-45	forest of aspen (*Populus tremulus*)
pitfalls 46-48	scattered birches with mainly dune reed (*Calamagrostis epigejos*) as undergrowth
pitfalls 49-51	open terrain with dense vegetation of dune reed (*Calamagrostis epigejos*), etc.
pitfalls 52-54	forest with a lot of common hop (*Humulus lupulus*)
pitfalls 55-57	forest of aspen (*Populus tremulus*)
pitfalls 58-60	forest of aspen (*Populus tremulus*)

Pitfalls 25-27 are located in the Bierlap (grasshopper plain) open terrain with European beachgrass (*Ammophila arenaria*) and sand sedge *Carex arenaria*,

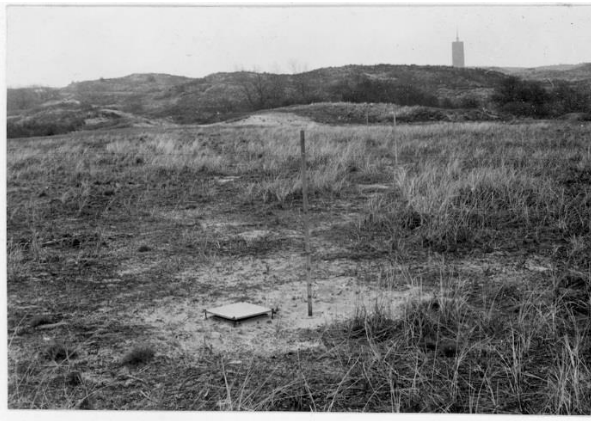

Pitfalls 25, 26 and 27 in April 1955

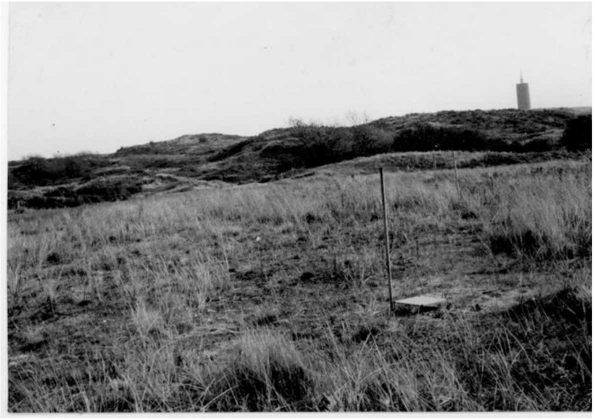

Pitfall 25, 26 and 27 in October1955

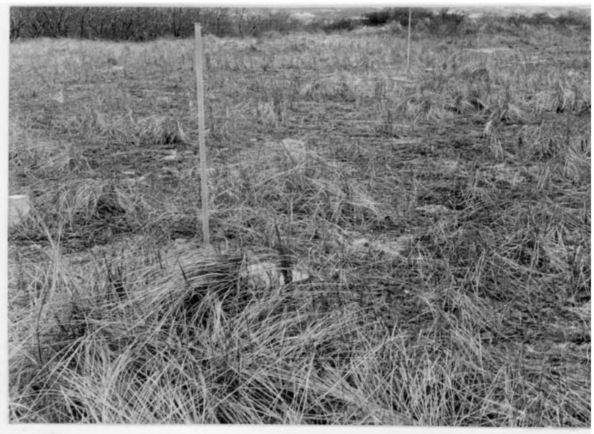

Pitfalls 26 and 27 in April 1955

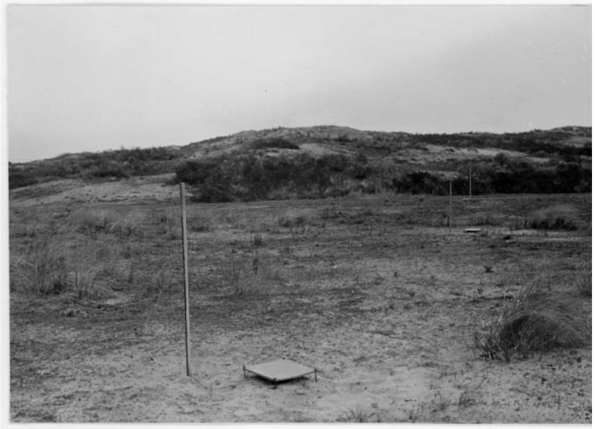

Pitfalls 28, 29 and 30 in September 1954

Pitfalls 28-30 are located in the Bierlap (grasshopper plain) open terrain with little or no vegetation except lichens

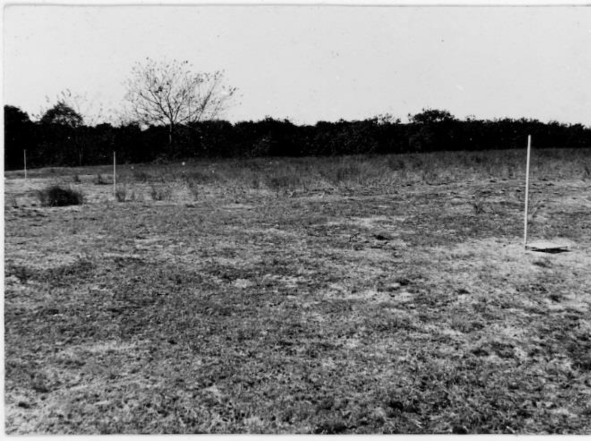

Pitfalls 30, 29 and 28 in September 1954

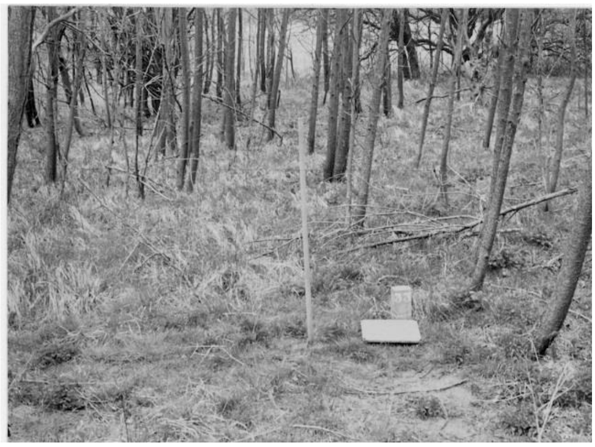

Pitfall 31 in April 1955

Pitfalls 31-33 are located in the Bierlap in a dense poplar forest with lots of litter, east of a concrete path.

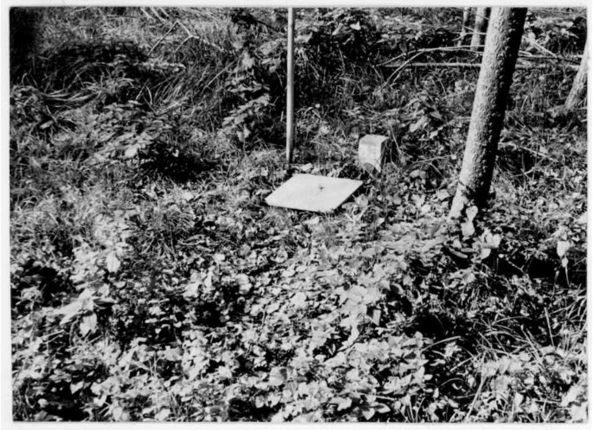
Pitfall 31 in September 1954

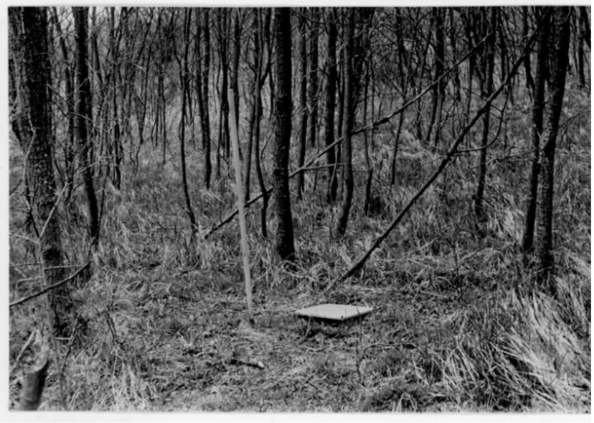
Pitfall 32 in April 1955

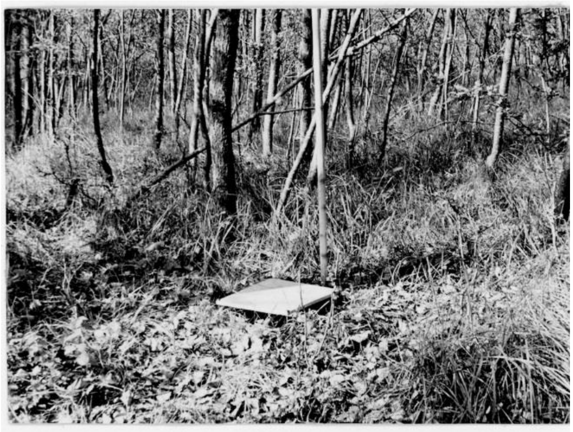
Pitfall 32 in September 1954

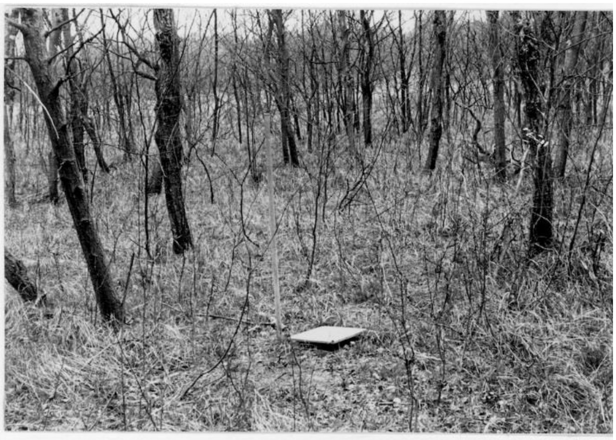
Pitfall 33 in April 1955

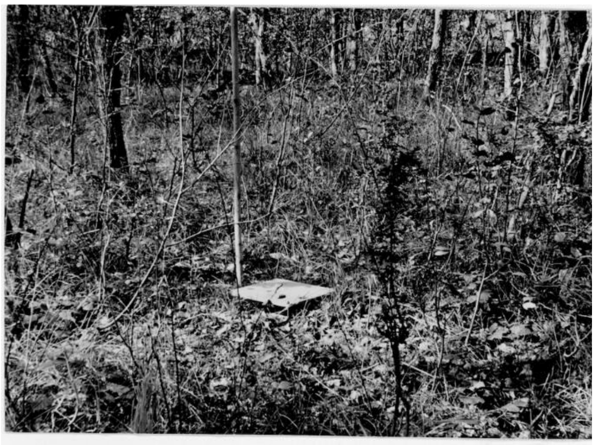
Pitfall 33 in September 1954

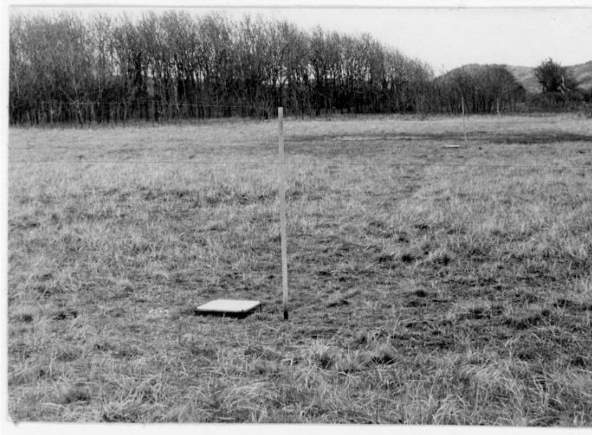
Pitfall 34 in April 1955 with 35 and 36 in the background

Pitfalls 34-36 are located in the Bierlap, in open terrain with dune reed (*Calamagrostis epigejos*), West of a concrete path.

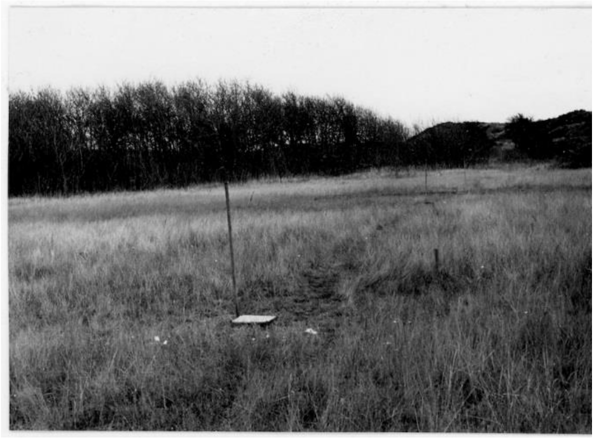

Pitfall 34 in November 1955 with 35 and 36 in the background

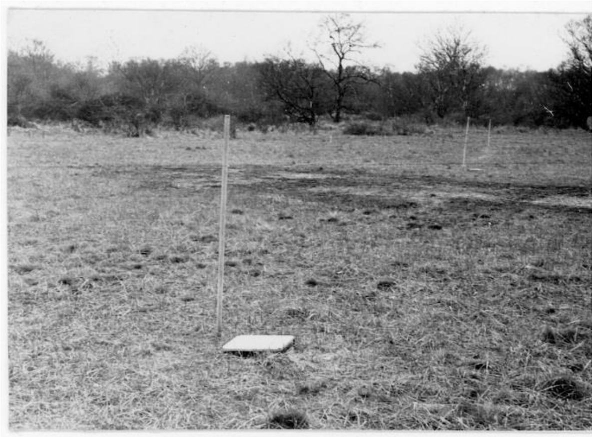

Pitfalls 36 in April 1955 with 35 and 34 in the background

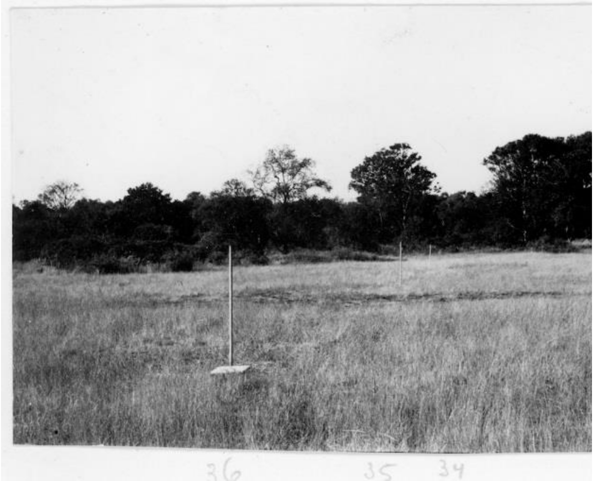

Pitfall 36 in October 1955 with 35 and 34 in the background

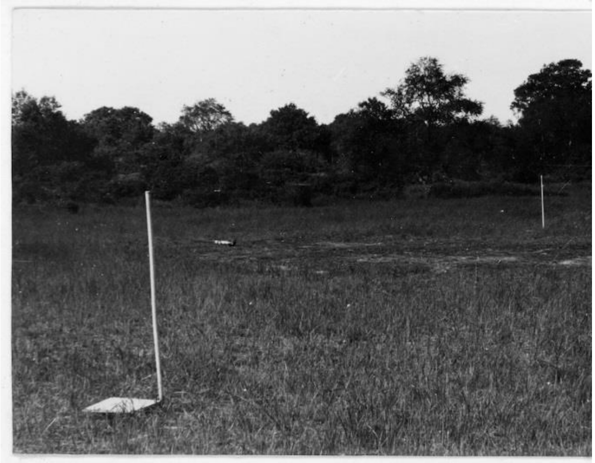

Pitfall 36 in September 1954 with 35 and 34 in the background

Pitfalls 37-39 are located in the Bierlap in open terrain with scattered birch and hawthorn (*Crataegus monogyna*), dune reed (*Calamagrostis epigejos*) and perforate St John's-wort (*Hypericum perforatum*), west of a concrete path and a fence.

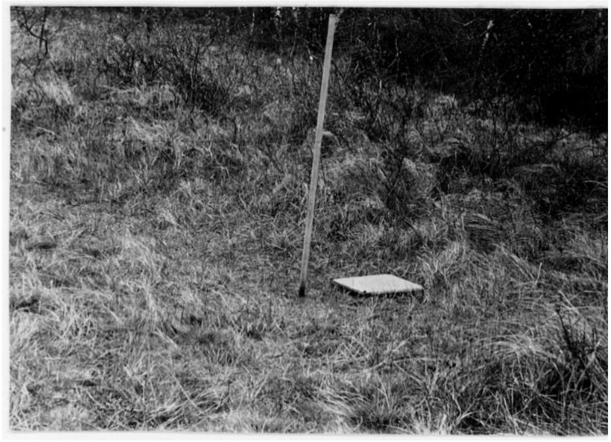

Pitfall 37 in April 1955

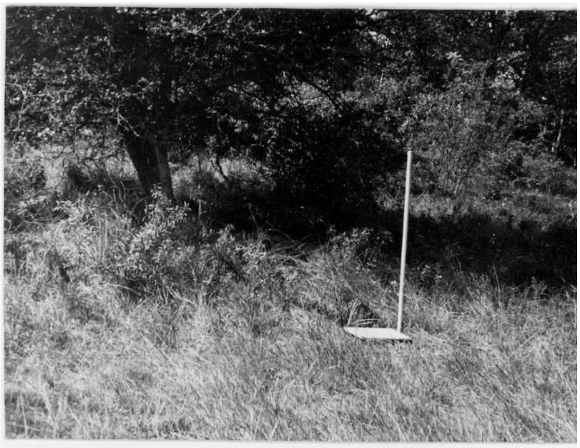

Pitfall 37 in September 1954

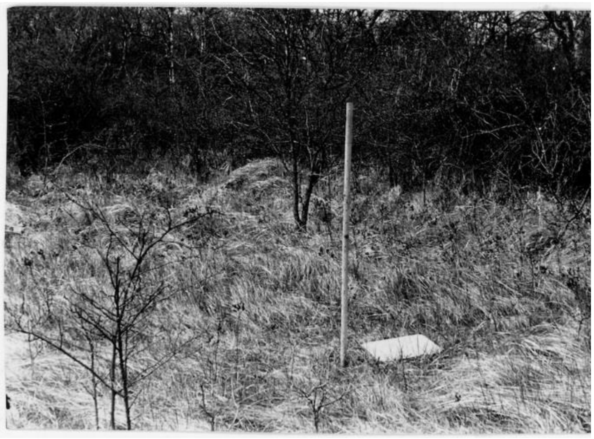

Pitfall 38 in April 1955

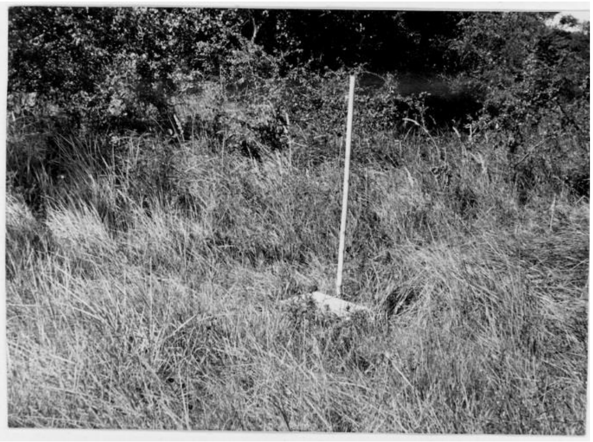

Pitfall 38 in September 1954

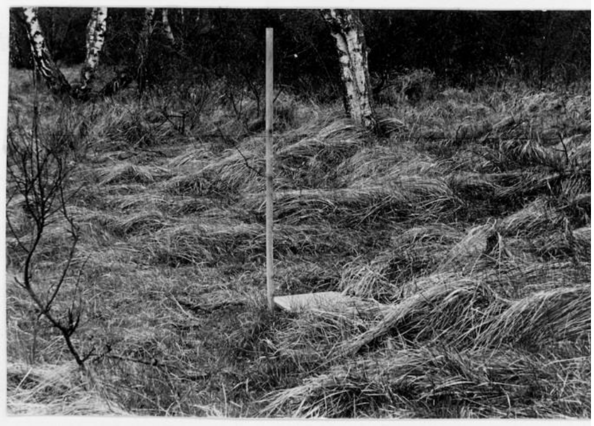

Pitfall 39 in April 1955

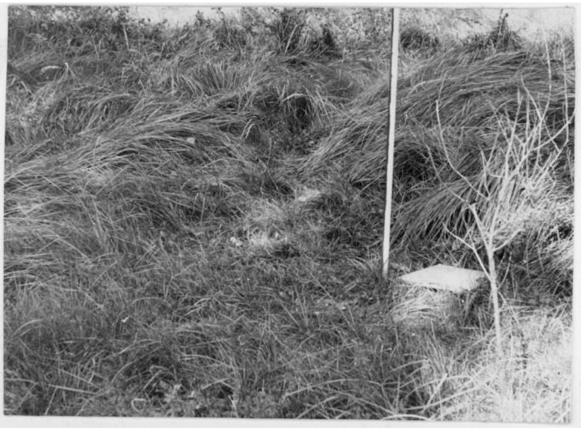

Pitfall 39 in September 1954

Pitfalls 40-42 are located in the Bierlap in an old sea buckthorn forest (*Hippophae rhamnoides*).

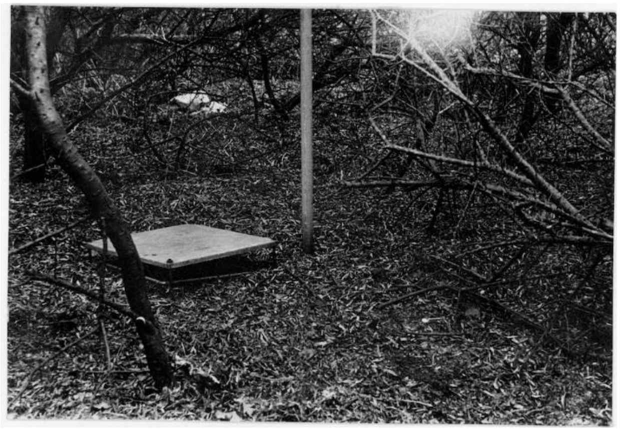
Pitfall 40 in April 1955

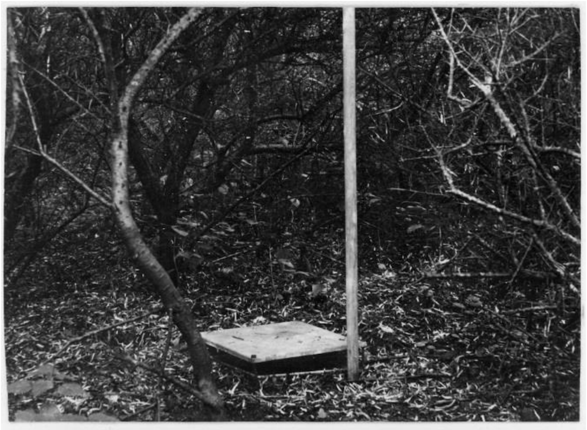
Pitfall 40 in September 1954

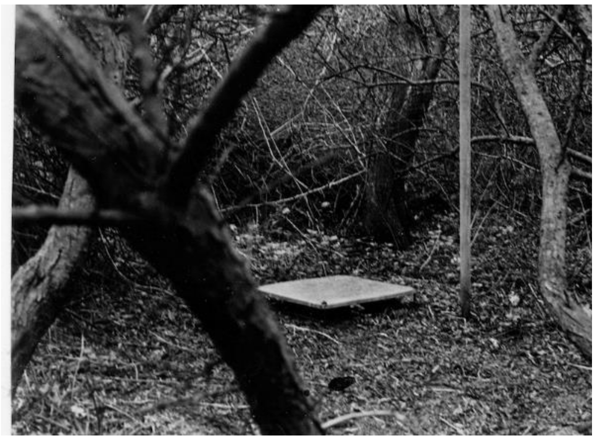
Pitfall 41 in April 1955

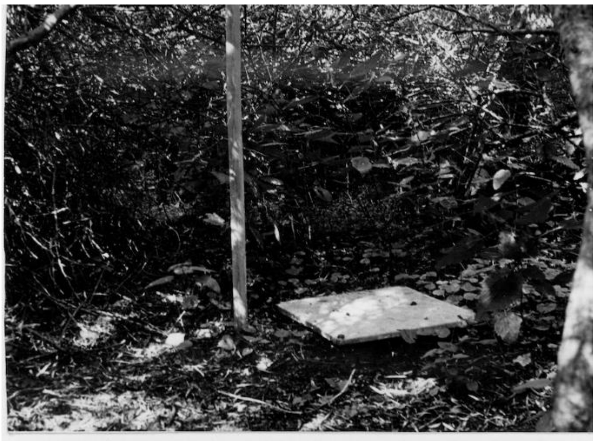
Pitfall 41 in September 1954

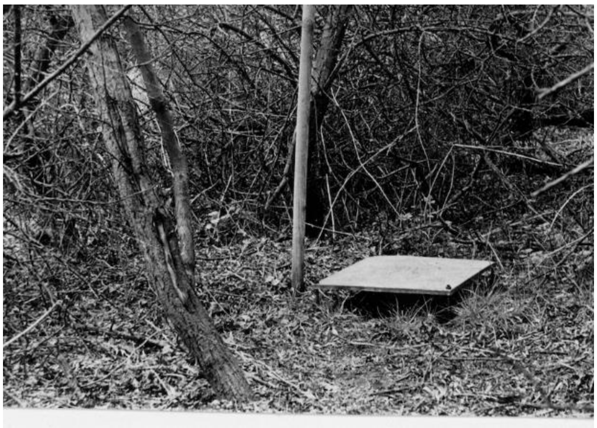
Pitfall 42 in April 1955

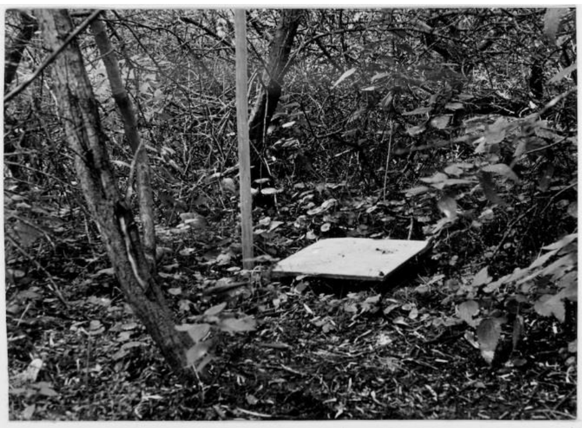
Pitfall 42 in September 1954

Pitfalls 43-45 are located in the Bierlap in a birch forest with old hawthorns (*Crataegus monogyna*), and quite a lot of litter.

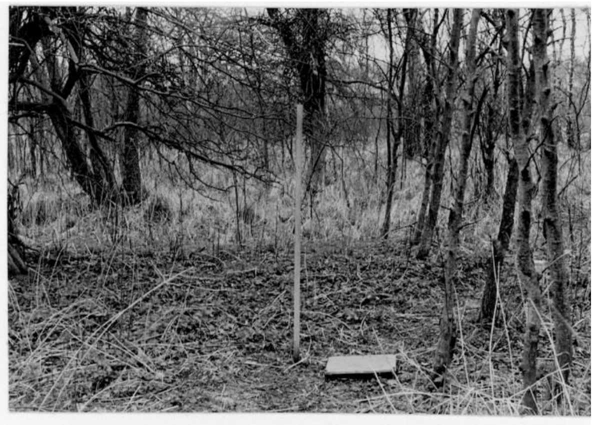

Pitfall 43 in April 1955

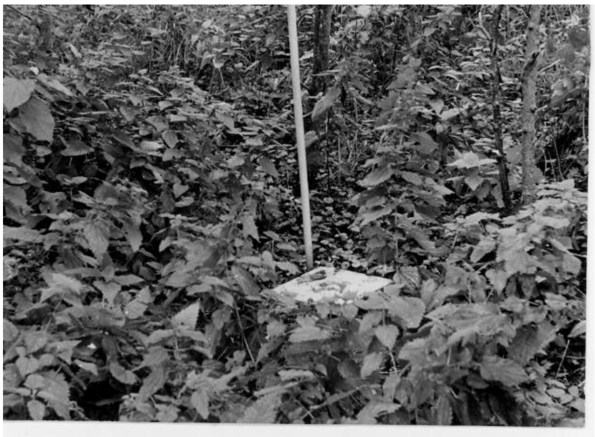

Pitfall 43 in September 1954

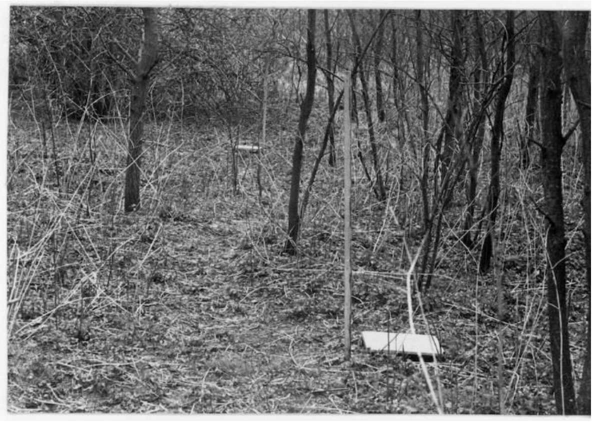

Pitfall 44 in April 1955 with pitfall 45 in the background

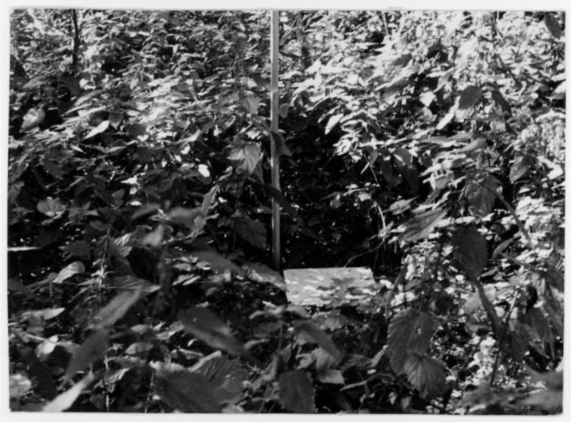

Pitfall 44 in September 1954

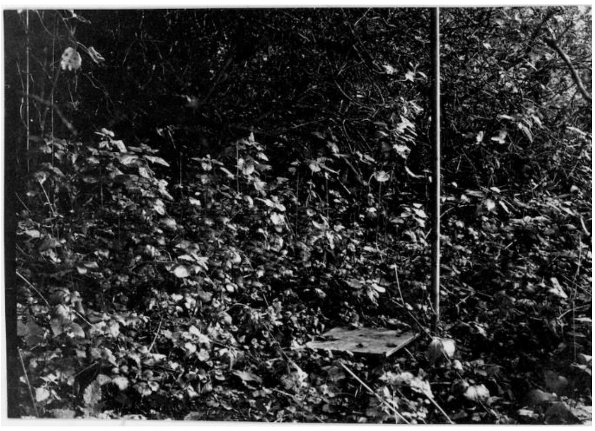

Pitfall 45 in October 1955

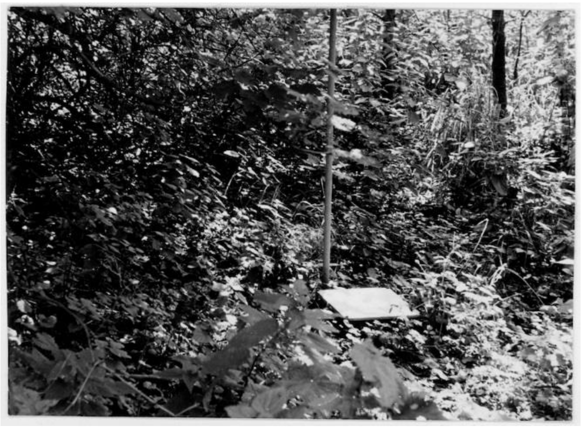

Pitfall 45 in September 1954

Pitfalls 46-48 are located in the Bierlap in open terrain with scattered birch, and dune reed (*Calamagrostis epigejos*).

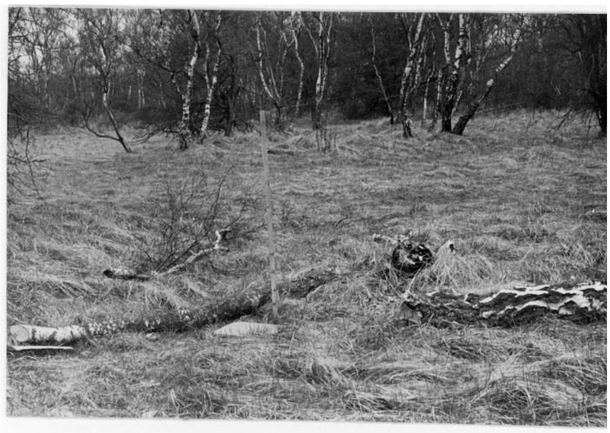

Pitfall 46 in April 1955

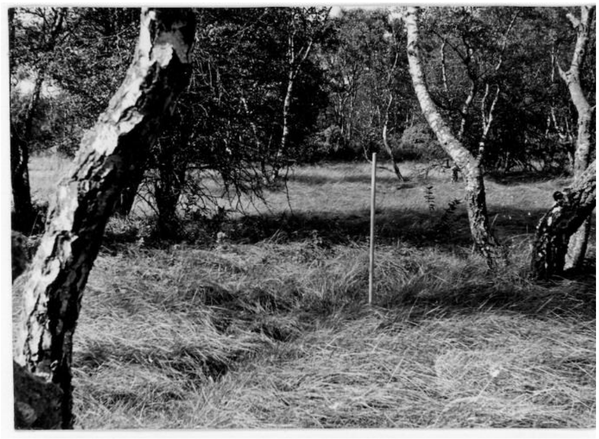

Pitfall 46 in September 1954

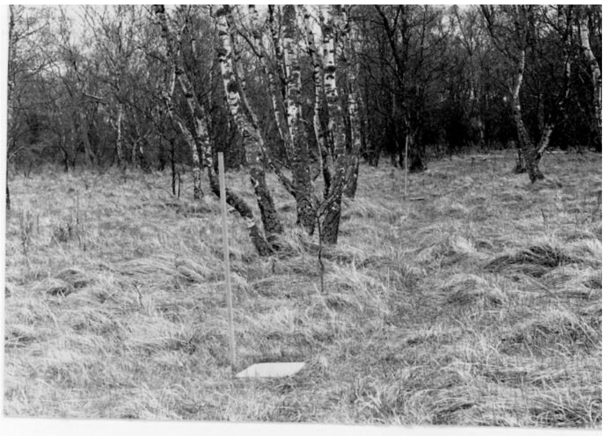

Pitfall 47 in April 1955 with pitfall 48 in the background

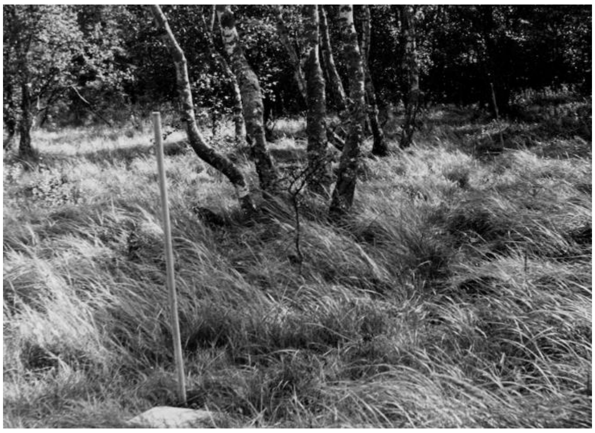

Pitfall 47 in September 1954 with pitfall 48 in the background

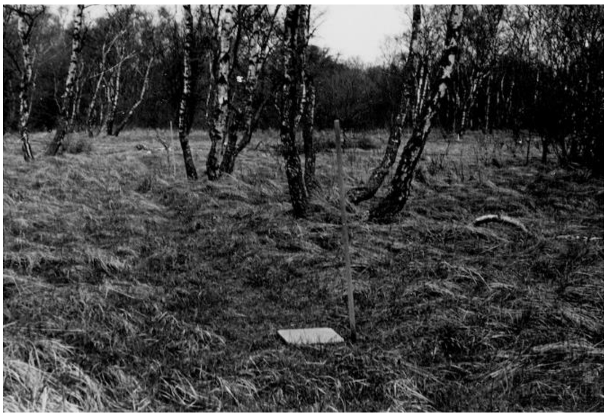

Pitfall 48 in April 1955 with pitfall 47 in the background

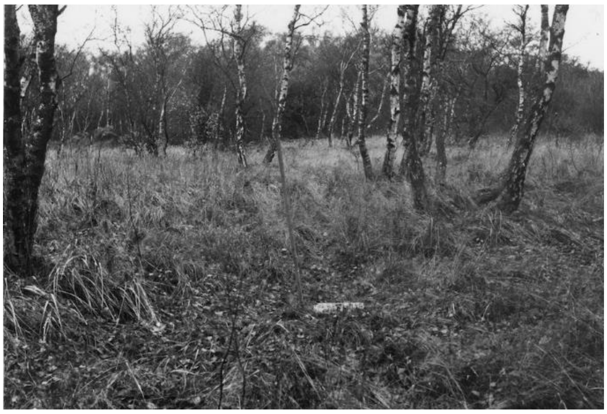

Pitfall 48 in November 1955 with pitfall 47 in the background

Pitfalls 49-51 are located in the Bierlap in a completely open terrain with dune reed (*Calamagrostis epigejos*).

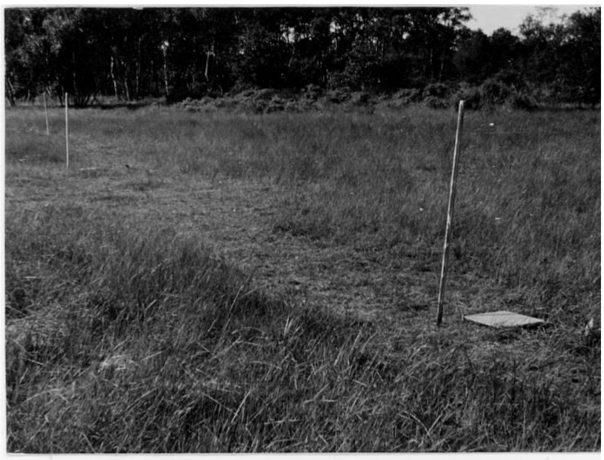

Pitfall 49 in September 1954 with pitfalls 50 and 51 in the background

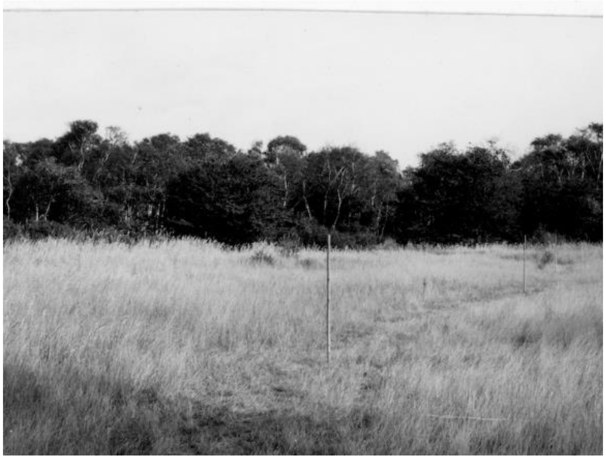

Pitfall 51 in October 1955 with pitfalls 50 and 49 in the background

Pitfalls 52-54 are located in the Bierlap in forest with a lot of hops (*Humulus lupulus*), probably anthropogenic terrain with maple (*Acer pseudoplatanus*).

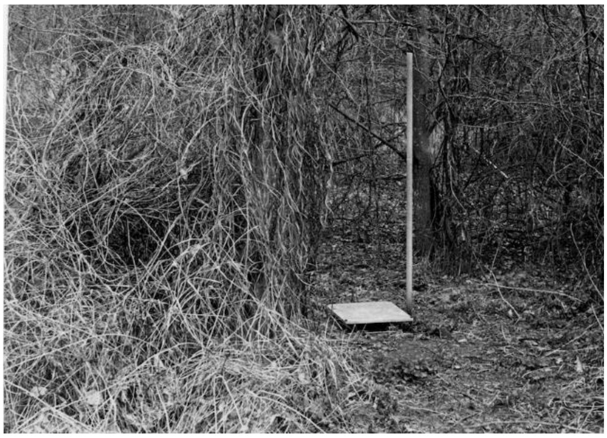

Pitfall 52 in April 1955

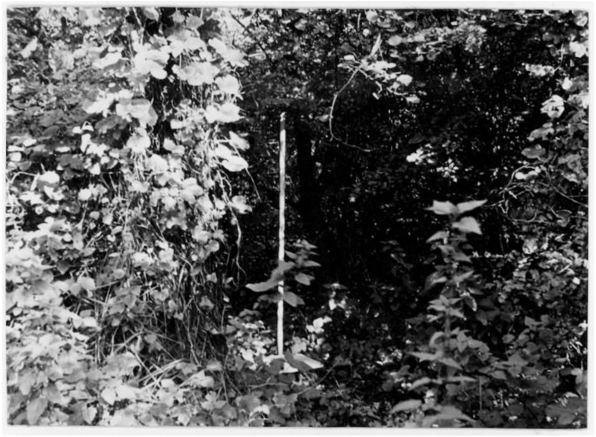

Pitfall 52 in September 1954

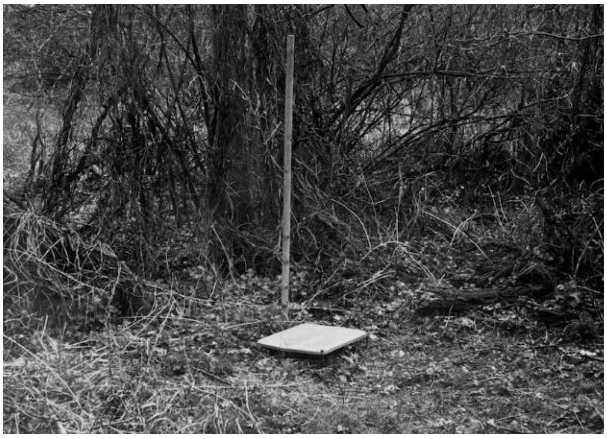

Pitfall 53 in April 1955

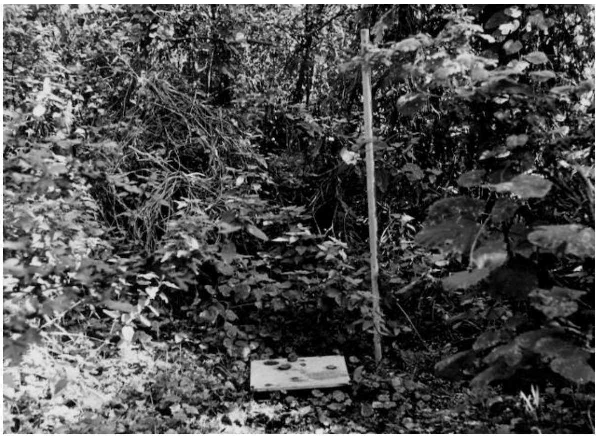

Pitfall 53 in September 1954

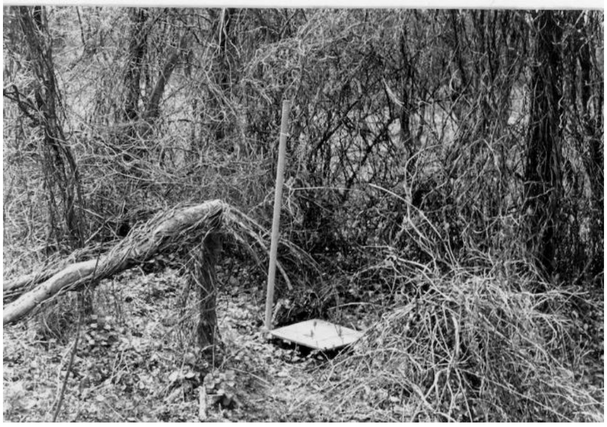
Pitfall 54 in April 1955

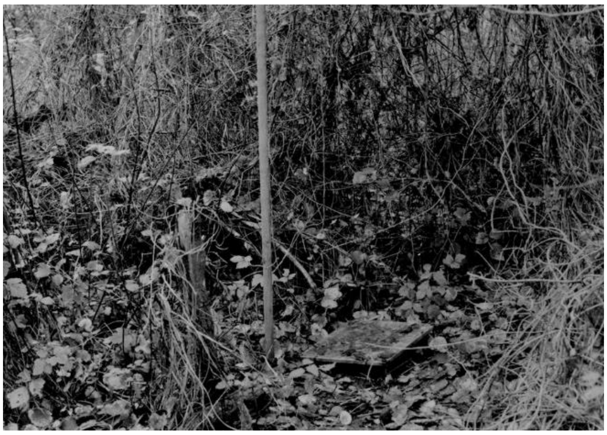
Pitfall 54 in November 1955

Pitfalls 55-57 are located in the Bierlap in poplar forest with hawthorns (*Crataegus monogyna*) and lots of litter, quite moist.

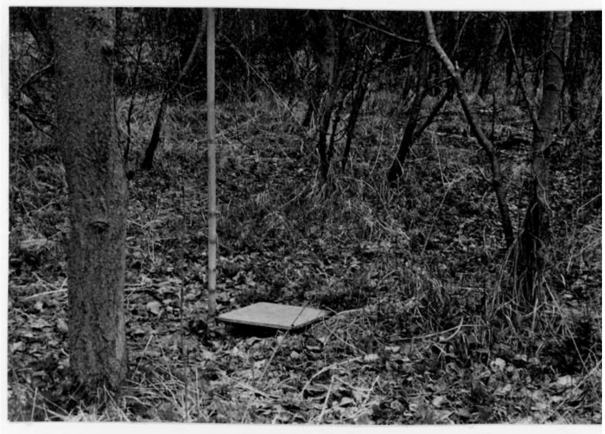
Pitfall 55 in April 1955

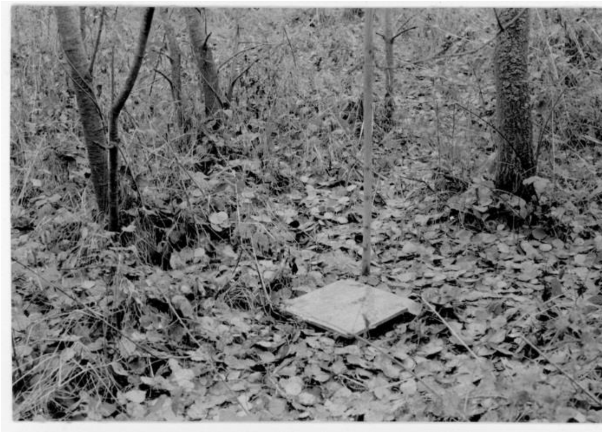
Pitfall 55 in November 1955

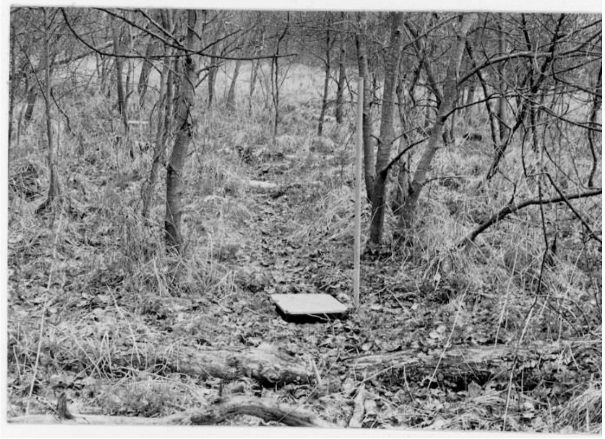
Pitfall 56 in April 1955

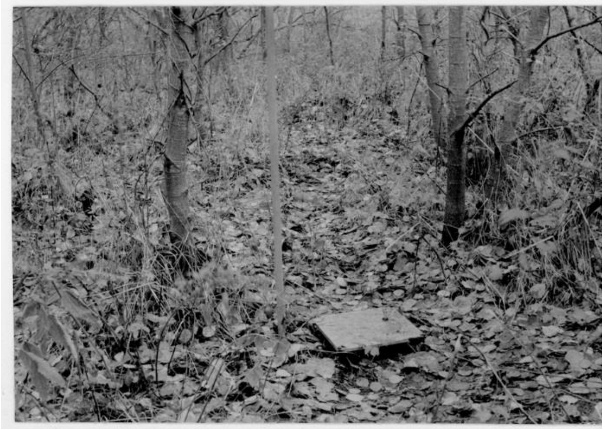
Pitfall 56 in November 1955

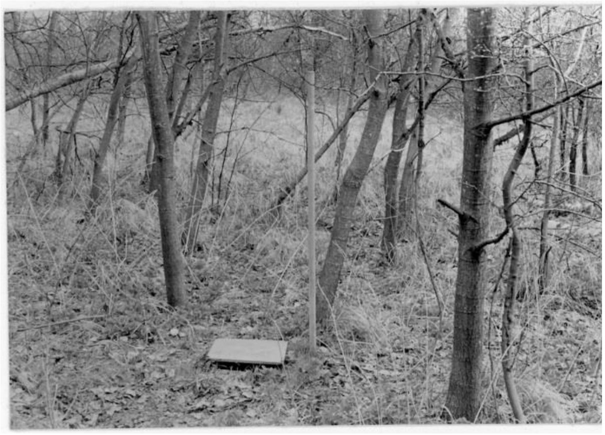
Pitfall 57 in April 1955

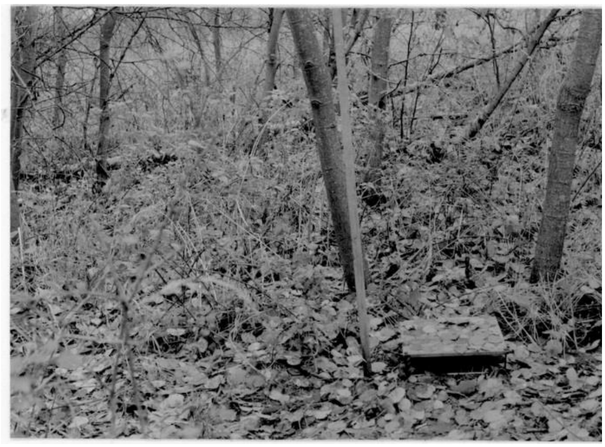
Pitfall 57 in November 1955

Pitfalls 58-60 are located in the Bierlap in a poplar forest with mid-diameter and lots of litter, on a somewhat higher elevation.

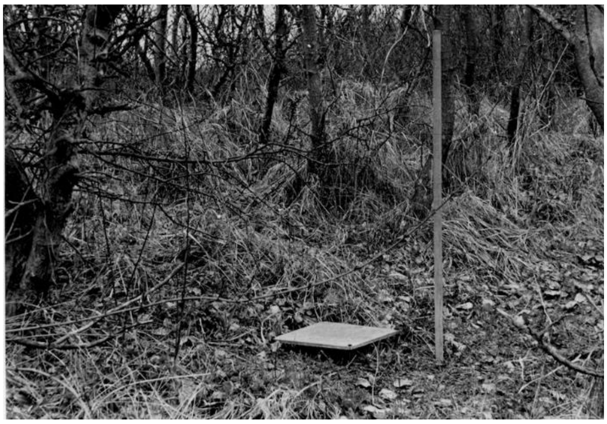

Pitfall 58 in April 1955

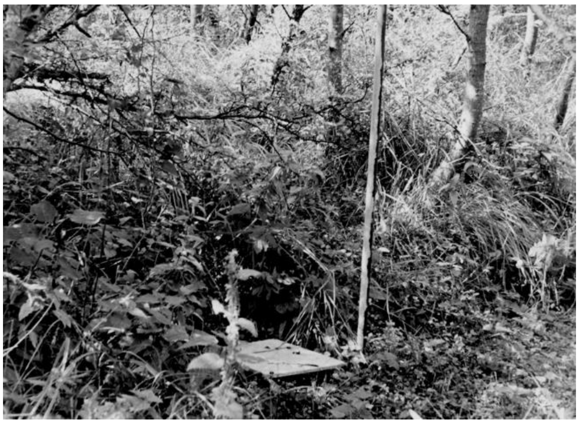

Pitfall 58 in September 1954

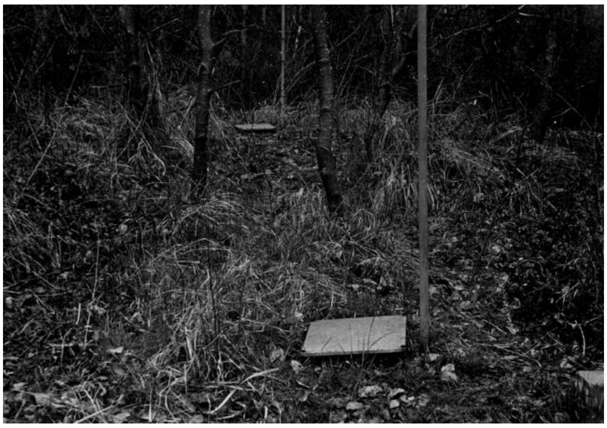

Pitfall 59 in April 1955

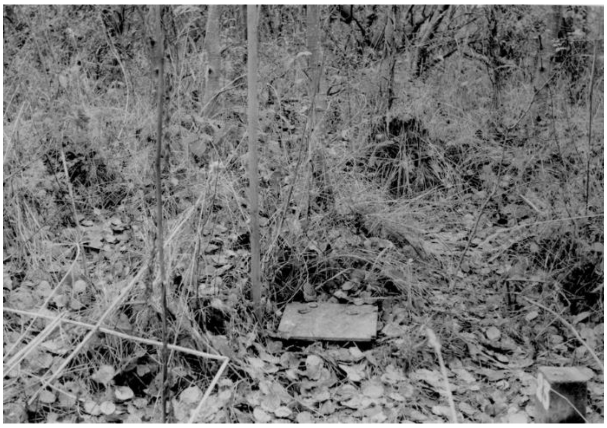

Pitfall 59 in November 1955

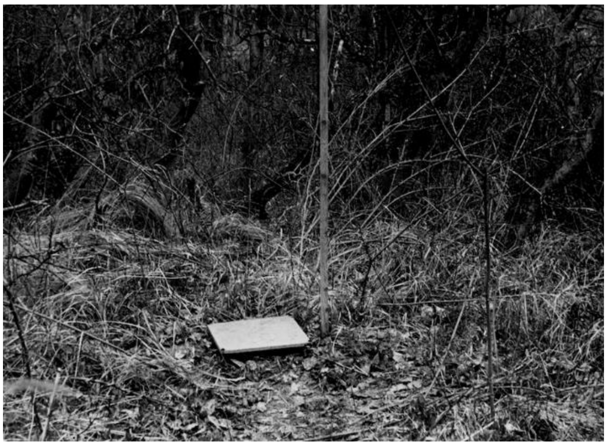

Pitfall 60 in April 1955

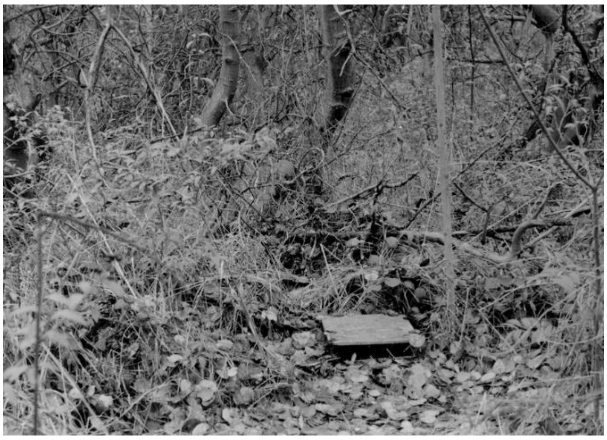

Pitfall 60 in November 1955

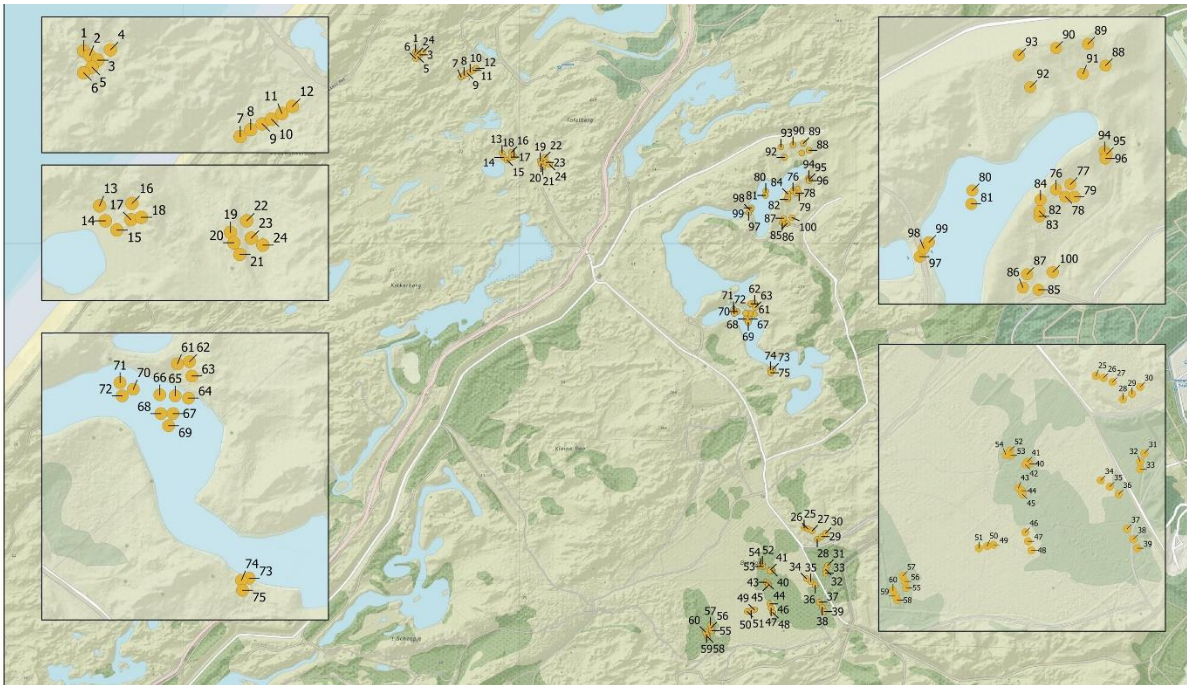

Map with details on location of pitfalls 1-100.
